# Epilepsy-Related Voltage-Gated Sodium Channelopathies: A Review

**DOI:** 10.3389/fphar.2020.01276

**Published:** 2020-08-18

**Authors:** Luis Felipe Santos Menezes, Elias Ferreira Sabiá Júnior, Diogo Vieira Tibery, Lilian dos Anjos Carneiro, Elisabeth Ferroni Schwartz

**Affiliations:** ^1^ Laboratório de Neurofarmacologia, Departamento de Ciências Fisiológicas, Universidade de Brasília, Brasília, Brazil; ^2^ Faculdade de Medicina, Centro Universitário Euro Americano, Brasília, Brazil; ^3^ Faculdade de Medicina, Centro Universitário do Planalto Central, Brasília, Brazil

**Keywords:** channelopathies, epilepsy, ion channel, mutation, sodium channel

## Abstract

Epilepsy is a disease characterized by abnormal brain activity and a predisposition to generate epileptic seizures, leading to neurobiological, cognitive, psychological, social, and economic impacts for the patient. There are several known causes for epilepsy; one of them is the malfunction of ion channels, resulting from mutations. Voltage-gated sodium channels (NaV) play an essential role in the generation and propagation of action potential, and malfunction caused by mutations can induce irregular neuronal activity. That said, several genetic variations in NaV channels have been described and associated with epilepsy. These mutations can affect channel kinetics, modifying channel activation, inactivation, recovery from inactivation, and/or the current window. Among the NaV subtypes related to epilepsy, NaV1.1 is doubtless the most relevant, with more than 1500 mutations described. Truncation and missense mutations are the most observed alterations. In addition, several studies have already related mutated NaV channels with the electrophysiological functioning of the channel, aiming to correlate with the epilepsy phenotype. The present review provides an overview of studies on epilepsy-associated mutated human NaV1.1, NaV1.2, NaV1.3, NaV1.6, and NaV1.7.

## Introduction

Epilepsy is a disease known worldwide, affecting around 70 million people in the world (Thijs et al., 2019). It has been considered a disease and no longer a disorder or a family of disorders since 2014 by International League Against Epilepsy (ILAE) and the International Bureau for Epilepsy (IBE) (Falco-Walter et al., 2018). Epilepsy is conceptually defined as a disease in which an individual has at least two unprovoked or reflex seizures in a period greater than 24 h apart, one unprovoked or reflex seizure and a probability of having another seizure similar to the general recurrence risk after two unprovoked seizures (greater than or equal to 60%) over the next ten years or an epilepsy syndrome (Fisher et al., 2014).

When abnormal brain activity begins in one or more identified regions, epilepsy is called focal, whereas, when it occurs in both hemispheres with a wide distribution, it is called generalized. Finally, when it cannot be classified as either focal or generalized, it is called unknown (Devinsky et al., 2018).

Epilepsy can affect anyone, regardless of gender, age, and income levels (Saxena and Li, 2017). Understanding the etiology of epilepsy is crucial for clinical management of patients and for conducting neurobiological research that will direct future therapies (Thomas and Berkovic, 2014). The ILAE Task Force has defined six etiologic categories; they are not hierarchical and more than one might often apply (structural, genetic, infectious, metabolic, immune, and unknown) (Falco-Walter et al., 2018).

Among those genetically caused, it is possible to identify several epilepsy-related genes (Lindy et al., 2018). For example, voltage-gated potassium channel, voltage-gated calcium channel and voltage-gated chloride channel genes, GABA receptors, nicotinic acetylcholine receptors, polymerase (DNA) Gamma genes and voltage-gated sodium channel genes (Deng et al., 2014).

Voltage-gated sodium channels (NaV) can be found mainly in the central nervous system (CNS), peripheral nervous systems (PNS), skeletal, and cardiac muscles (Huang et al., 2017). NaVs are distributed throughout the body and play an important role in the generation and propagation of action potential (Wang et al., 2017b). Structurally, NaVs are composed by an α subunit organized in four homologous ligated domains (DI-DIV), each domain composed by six transmembrane segments (S1-S6), and one or more β subunits associated by non-covalent interactions or disulfide bond (Abdelsayed and Sokolov, 2013; Gilchrist et al., 2013; Catterall, 2017; Bouza and Isom, 2018; Jiang et al., 2020). The domains of an α subunit present a high degree of conservation with each other, presenting the region known as the voltage sensor domains (VSD) located in transmembranes S1-S4, especially S4 helix, which contains positively charged residues, and the pore-forming (PM) domain located in S5-S6 segments, structuring a four VSD around a central pore (Ahern et al., 2016).

The S4 helix of DI, DII, and DIII domains moves faster than the S4 helix of DIV during membrane depolarization, and this asynchronous movement is an essential feature in the steady activation voltage-dependent process, which provokes movement of S4-S5 intracellular links followed by the displacement of the S6 segments to initiate Na^+^ influx (Goldschen-Ohm et al., 2013; Oelstrom et al., 2014). The movement of the S4 helix of DIV initiates the process of fast inactivation, since the movement of the voltage sensor in domain DIV is associated with the displacement of an intracellular loop between DIII and DIV within an IFM (isoleucine, phenylalanine, and methionine) motif that binds intracellular to PM and terminate Na^+^ influx (Capes et al., 2013; Clairfeuille et al., 2019). A second type of reversible inactivation occurs after repetitive or prolonged stimulation and results in steady-state inactivation whose asymmetric movement of S6 segments collapses the pore (Payandeh et al., 2012; Zhang et al., 2012; Gamal El-Din et al., 2013; Silva and Goldstein, 2013; Ghovanloo et al., 2016). Consequently, electrophysiological changes such as increased current density, shifting steady-state activation, and inactivation to negative and positive values, respectively, enhanced persistent current, accelerated recovery from inactivation, and delayed fast inactivation can cause gain-of-function (GoF) in the channel. Also, decreased current density, positive shift in steady-state activation, negative shift in steady-state inactivation, and slower recovery from inactivation can cause loss-of-function (LoF) (Mantegazza et al., 2005; Liao et al., 2010; Lossin et al., 2012; Catterall, 2014b; Vanoye et al., 2014; Wagnon et al., 2017; Yang et al., 2018; Zaman et al., 2018; Wengert et al., 2019; Zhang S. et al., 2020).

Currently, there are nine different alpha subtypes of NaVs (NaV1.1-NaV1.9), and mutations in these channels can cause diseases known as channelopathies (Catterall et al., 2010). NaV1.1 (*SCN1A*), NaV1.2 (*SCN2A*), NaV1.3 (*SCN3A*), NaV1.6 (*SCN8A*) and NaV1.7 (*SCN9A*) are genes whose mutations are related to epilepsy. So far, there is no correlation of mutations in NaV1.4 (*SCN4A*), NaV1.5 (*SCN5A*), NaV1.8 (*SCN10A*), and NaV1.9 (SCN11A) with epilepsy, which is to be expected, since these channels are mainly expressed in skeletal muscles, cardiac tissues, dorsal root ganglia, trigeminal sensory neurons, nociceptive neurons of the dorsal root and trigeminal ganglia, respectively (Brunklaus et al., 2014). Both α and β subunits (*SCN1B*) have been reported as the cause of epilepsy phenotype (Meisler et al., 2010; Kaplan et al., 2016).

NaV channels rank amongst the 2% most conserved proteins in the human genome, with an extremely low rate of coding variation, accounting for nearly 5% of known epileptic encephalopathies (Petrovski et al., 2013; Mercimek-Mahmutoglu et al., 2015; Lek et al., 2016; Heyne et al., 2019). Pathogenic mutated residues are situated in the highly evolutionarily conserved portions of the channel: transmembrane segments, intracellular inactivation gate loop, and the proximal 2/3 of the C-terminal domain (Blanchard et al., 2015; Wagnon and Meisler, 2015). The final 1/3 portion of the C-terminal and cytoplasmic interdomain loops 1 and 2 are less conserved (Denis et al., 2019). The proximal 2/3 of the C-terminal are involved in the interaction of several binding sites for proteins and accessory molecules, like beta subunits β1 and β3, fibroblast growth factors (molecules implicated in neural development), calmodulin (regulatory protein in neuronal function and hyperexcitability) and G protein (Bähler and Rhoads, 2002; Spampanato, 2004; Wittmack et al., 2004; Laezza et al., 2009; Yang et al., 2010). Moreover, the C-terminal has been shown to interact with the inactivated channel via ionic interaction between its positively charged residues and negatively charged residues at the inactivation gate. A shift in any of the charges can brake electrostatic interaction and affect normal channel inactivation (Nguyen and Goldin, 2010; Shen et al., 2017; Johnson et al., 2018).

The N-terminal region seems to play a more important role on protein trafficking than on channel activity. This domain interacts with the light chain of microtubule-associated protein MAP1B, facilitating the traffic of the NaV channel to the neuronal cell surface (O’brien et al., 2012; Blanchard et al., 2015). In addition, mutation in the N-terminal leads to protein retention in the endoplasmic reticulum (Sharkey et al., 2009).

Newer genomic approaches, especially next generation sequencing (NGS), improve the rate and reduce the costs associated with genetic epilepsy diagnosis, since traditional cytogenetic and microarray-based tests are lengthy, expensive, and diagnostic yield is incredibly low (Veeramah et al., 2013; Allen et al., 2016; Sands and Choi, 2017; Orsini et al., 2018). The use of gene panels and whole-exome sequencing (WES) provides a powerful tool to change the paradigm of genetic epilepsy diagnosis (Ng et al., 2010; Clark et al., 2018). These techniques have been widely used to elucidate suspected inherited neurological diseases in the last years, contributing to dramatically increase the number of patients diagnosed with genetic epilepsy. Both mendelian and *de novo* genetic epilepsy can be detected with these methods, but doubtless, *de novo* mutations are the most prevalent mutations related to epilepsy-related voltage-gated sodium channel mutations.

Gene therapy is promising as an effective approach to treat genetic diseases. Personalized epilepsy therapies are in development and have shown promising results, ranging from antisense oligonucleotides and small peptides to modulation of gene expression through epigenetics (Riban et al., 2009; Tan et al., 2017; Stoke Therapeutics, 2018; Perucca and Perucca, 2019). Even eating habits may be related to an improvement in the patient's clinical condition. Ketogenic diet has been described as an effective treatment in epilepsy (Gardella et al., 2018). Moreover, the combination of traditional antiepileptic drugs with new compounds displayed a synergic and improved efficacy, since these molecules do not compete for the same interaction site (Bialer et al., 2018). Each specific epilepsy-related NaV isoform will be presented and discussed in detail in the following sections.

## NaV Mutations

### NaV1.1

The *SCN1A* gene encodes for the α subunit NaV1.1, and is allocated at the 2q24.3 chromosome between 165,984,641 and 166,149,161 base pairs, same gene cluster of *SCN2A-SCN3A* genes, being the most frequent target of mutation in genetic epilepsy syndromes (OMIM#182389) (Malo et al., 1991; Malo et al., 1994; Catterall et al., 2010). NaV1.1 is widely expressed in the CNS, predominant in inhibitory GABAergic interneurons, regulating neuronal excitability, and the reduction of its activity is one of the factors that cause epileptic diseases due to imbalance between inhibition and excitation (Yu et al., 2006; Verret et al., 2012; Tai et al., 2014; Rubinstein et al., 2015).

Epilepsy syndromes, such as generalized epilepsy with febrile seizures plus (GEFS+; Online Mendelian Inheritance in Man [OMIM] #604233), severe myoclonic epilepsy (SME) and SMEI, also known as Dravet syndrome (OMIM #607208), are associated with mutations in the *SCN1A* gene (Escayg and Goldin, 2010; Meng et al., 2015; Huang et al., 2017).

In the *SCN1A* mutation database (http://www.caae.org.cn/gzneurosci/scn1adatabase/data), among 1727 mutations described for the *SCN1A* gene, 1528 are related to epileptic diseases (**Table 1** and for the full description of mutations in the SCN1A gene, see **Supplementary Table S1**). Among the epilepsy-related mutations, 945 are related to severe myoclonic epilepsy of infancy (SMEI), 263 are related to severe myoclonic epilepsy (SME), 151 are related to severe myoclonic epilepsy borderline (SMEB), 18 are related to partial epilepsy (PE), 31 are related to partial epilepsy and febrile seizures plus (PEFS +), 8 are related to generalized epilepsy (GE), and 55 are related to generalized epilepsy with febrile seizures plus (GEFS +).

**Table 1:** SCN1A-related epilepsies identified in clinical patients through WES and/or NGS.

Variant	Location	Mutation	Disease	Alteration on *biophysical properties* or/and Clinical report	Reference
**Inherited mutation**					
**A27T**	N-terminal	Missense	GEFS+SMEB	Diffuse spikes, prevailing in posterior regions (EEG)	(Nicita et al., 2010)
**L61P**	N-terminal	Missense	DS	Febrile seizures	(Halvorsen et al., 2016)
**F63L**	N-terminal	Missense	DS	Severe developmental delay Spike and Waves in right fronto-temporal region with spreading (EEG)	(Nicita et al., 2010)
**F90S**	N-terminal	Missense	DS	Multifocal spikes, frontal-dominant spike-waves complex (EEG)	(Sun et al., 2008; Wang et al., 2012; Xu et al., 2014; Butler et al., 2017b)
**S103G**	N-terminal	Missense	SME DS	Ataxia Rare-spike wave complex (EEG)	(Fujiwara, 2003; Ebrahimi et al., 2010; Tonekaboni et al., 2013)
**S106F**	N-terminal	Missense	Focal epilepsy	Right temporal parietal occipital slow-wave and generalized spike-wave complex (EEG)	(Barba et al., 2014)
**M145T**	DI (S1)	Missense	Unidentified epilepsy	Decrease current density Shift steady-state inactivation to more positive values	(Mantegazza et al., 2005; Colosimo et al., 2007)
**L193F**	DI (S3)	Missense	GEFS+	Generalized tonic–clonic seizures	(Cui et al., 2011)
**V244L**	DI (S4-S5)	Missense	DS	Myoclonic seizures Generalized spikes or spike-and-wave complexes in the interictal (EEG)	(Morimoto et al., 2006)
**R377Q**	DI (S5-S6)	Missense	GEFS+	Generalized tonic-clonic seizures	(Zucca et al., 2008; Xu et al., 2015; Cetica et al., 2017; Lindy et al., 2018)
**F412I**	DI (S6)	Missense	SMEB GEFS+	Febrile seizure	(Ebrahimi et al., 2010; Tonekaboni et al., 2013)
**K488EfsX6**	DI-DII	FrameShift	DS	NR	(Yang et al., 2017)
**R542Q**	DI-DII	Missense	GEFS+ SME	NR	(Escayg et al., 2001; Weiss et al., 2003; Combi et al., 2009; Orrico et al., 2009; Wang et al., 2012; Lee et al., 2014; Lal et al., 2016)
**R618C**	DI-DII	Missense	PEFS+	Generalized tonic-clonic seizures Multifocal epilepsy and bilateral bursts of 3-4 Hz spike and wave (EEG)	(Brunklaus et al., 2015)
**Y790C**	DII (S1-S2)	Missense	GEFS+	Decreased current density Decreased of cell surface expression	(Annesi et al., 2003; Orrico et al., 2009; Bechi et al., 2015; Bennett et al., 2017)
**R859H**	DII (S4)	Missense	GEFS+	Shift steady state activation and inactivation to more negative values Enhanced Persistent current	(Volkers et al., 2011; Myers et al., 2017a; Lindy et al., 2018)
**S1084C**	DII-DIII	Missense	Juvenile myoclonic epilepsy DS	Paroxysmal generalised polyspike-and- wave complexes with myoclonic seizures (EEG)	(Jingami et al., 2014)
**T1174S**	DII-DIII	Missense	FHM FS	Shift steady state activation to more positive values Deceleration of recovery from fast inactivation Increase of persistent current	(Escayg et al., 2001; Gargus and Tournay, 2007; Yordanova et al., 2011; Rilstone et al., 2012; Cestèle et al., 2013; Lal et al., 2016)
**V1353L**	DIII (S5)	Missense	PEFS+ GEFS+	Non-functional channel	(Wallace et al., 2001; Lossin et al., 2003; Bennett et al., 2017)
**A1429S**	DIII (S5-S6)	Missense	Autossomal dominant nocturnal frontal lobe epilepsy	No definitive epileptic spikes (EEG)	(Sone et al., 2012)
**R1596H**	DIV (S2-S3)	Missense	GEFS+	Generalized spike-wave complexes (EEG) Normal imaging (MRI)	(Hoffman-Zacharska et al., 2015)
**I1656M**	DIV (S4)	Missense	GEFS+	Shift steady state activation to more positive values	(Lossin et al., 2003)
**G1674S**	DIV (S5)	Missense	FS+	Febrile seizure Hemiconvulsion	(Saitoh et al., 2015a)
***De novo* mutation**					
**Q3X**	N-terminal	Nonsense	DS	Generalized tonic clonic seizures	(Claes et al., 2003; Lim et al., 2011)
**G58X**	N-terminal	Nonsense	DS Focal Epilepsy	Autistic characteristics; Hyperactivity Periventricular nodular heterotopia (MRI)	(Barba et al., 2014)
**Y65X**	N-terminal	Nonsense	DS	Generalized tonic-clonic seizures	(Zucca et al., 2008)
**E75D**	N-terminal	Missense	DS	Slow-spike-wave complexes (EEG)	(Arafat et al., 2017)
**L80_D81del**	N-terminal	Inframe deletion	DS	Pharmacoresistant	(Usluer et al., 2016)
**D81N**	N-terminal	Missense	DS	Severe Motor and mental delay Multi-focal spike-waves (EEG)	(Usluer et al., 2016)
**I91T**	N-terminal	Missense	DS	Frontal-dominant spike-waves complex (EEG)	(Sun et al., 2008; Xu et al., 2014)
**G96EfsX24**	N-terminal	FrameShift	NR	Genetic generalized epilepsy with intellectual disability	(Fry et al., 2016)
**R101Q**	N-terminal	Missense	DS SMEB GEFS+ PEFS+	Psychomotor retardation	(Fukuma et al., 2004; Harkin et al., 2007; Marini et al., 2007; Depienne et al., 2008; Sun et al., 2010; Zuberi et al., 2011; Wang et al., 2012; Tonekaboni et al., 2013; Lee et al., 2014; Djémié et al., 2016)
**A104V**	N-terminal	Missense	DS	Epileptic discharges, slow spike and weave; sharp wave, sharp and slow wave complex (EEG)	(Kwong et al., 2012; Myers et al., 2017a)
**R118S**	N-terminal	Missense	DS	Generalized tonic-clonic seizures Severe mental retardation	(Zucca et al., 2008)
**F144YfsX5**	DI (S1)	Frameshift	SME DS	Moderate psychomotor retardation	(Fukuma et al., 2004; Zuberi et al., 2011; Wang et al., 2012; Villeneuve et al., 2014)
**M145DfsX4**	DI (S1)	Frameshift	PEFS+	Generalized tonic-clonic seizures without any provoked factors	(Yu et al., 2010)
**G177E**	DI (S2-S3)	Missense	SME DS	Non-functional channel	(Nabbout et al., 2003; Ohmori et al., 2006; Usluer et al., 2016)
**L180X**	DI (S2-S3)	Nonsense	DS	Focal spike wave (EEG)	(Liu et al., 2018)
**W190X**	DI (S3)	Nonsense	DS	Febrile, partial, generalized tonic-clonic and myo-clonic seizures Severe intellectual disability	(Marini et al., 2007; Kwong et al., 2012)
**S213W**	DI (S3-S4)	Missense	Epilepsy	Febrile and afebrile seizures Developmental delay	(Butler et al., 2017a)
**R219SfsX57**	DI (S4)	FrameShift	DS	Generalized tonic-clonic seizures	(Claes et al., 2001)
**R222X**	DI (S4)	Nonsense	DS SMEB	No measurable current	(Claes et al., 2001; Nabbout et al., 2003; Fukuma et al., 2004; Harkin et al., 2007; Depienne et al., 2008; Orrico et al., 2009; Zuberi et al., 2011; Wang et al., 2012; Xu et al., 2014; Esterhuizen et al., 2018)
**I227S**	DI (S4)	Missense	SME SMEB	Epileptiform discharges on both sides and spikes/polyspikes during photic stimulation (EEG) Low current density (no detectable)	(Nabbout et al., 2003; Ohmori et al., 2006; Depienne et al., 2008; Mak et al., 2011; Wang et al., 2012; Lindy et al., 2018)
**A239V**	DI (S4-S5)	Missense	SME DS	Focal right fronto-temporal spikes with spreading (EEG) Severe developmental delay	(Iannetti et al., 2009; Nicita et al., 2010; Xu et al., 2014)
**W280R**	DI (S5-S6)	Missense	DS	Febrile seizures Status epilepticus Myoclonic Multifocal discharges (EEG)	(Nabbout et al., 2003; Wang et al., 2012; Liu et al., 2018)
**P281L**	DI (S5-S6)	Missense	DS	Moderate mental retardation	(Depienne et al., 2008; Gokben et al., 2017; Lindy et al., 2018)
**E311X**	DI (S5-S6)	Nonsense	DS	Haploinsufficiency	(Orrico et al., 2009)
**G329A**	DI (S5-S6)	Missense	GEFS+	Generalized tonic–clonic seizures	(Myers et al., 2017a)
**G343E**	DI (S5-S6)	Missense	SMEB SME DS	Spike-wave complex, Multifocal spikes (EEG)	(Fujiwara, 2003; Depienne et al., 2008; Zuberi et al., 2011)
**D366E**	DI (S5-S6)	Missense	DS	Generalized tonic-clonic seizures	(Zucca et al., 2008)
**W384R**	DI (S5-S6)	Missense	DS SMEB SME	Generalized tonic-clonic seizures Partial seizures	(Zuberi et al., 2011; Wang et al., 2012; Verbeek et al., 2013)
**T391P**	DI (S5-S6)	Missense	DS	Generalized tonic-conic seizures Partial Seizures	(Reyes et al., 2011)
**R393H**	DI (S5-S6)	Missense	DS SMEB	Generalized tonic-clonic seizures Myoclonus, Febrile seizures Developmental delay	(Claes et al., 2003; Marini et al., 2007; Sun et al., 2010; Zuberi et al., 2011; Lemke et al., 2012; Rilstone et al., 2012; Wang et al., 2012; Xu et al., 2014; Djémié et al., 2016; Haginoya et al., 2018)
**V422L**	DI (S6)	Missense	EE	Psychomotor developmental delay Theta activities with right predominance (EEG)	(Ohashi et al., 2014)
**Y426N**	DI-DII	Missense	DS	Decreased current density shift stead-state inactivation to more negative values Delayed recovery from inactivation	(Nabbout et al., 2003; Ohmori et al., 2006; Allen et al., 2016)
**L433fsX16**	DI-DII	FrameShift	Myoclonic astatic epilepsy	Generalized tonic-clonic seizures	(Ebach et al., 2005)
**E435X**	DI-DII	Nonsense	DS	Myoclonic seizures Atypical absence	(Fukuma et al., 2004; Wang et al., 2012)
**Q554H**	DI-DII	Missense	DS	Generalized tonic-clonic seizure Atonic and myoclonic seizures	(Skjei et al., 2015)
**S662X**	DI-DII	Nonsense	PEFS+	Generalized tonic-clonic seizures	(Yu et al., 2010)
**W738X**	DI-DII	Nonsense	SME	Febrile seizures Generalized tonic-clonic Severe intellectual disability	(Kwong et al., 2012; Xu et al., 2014)
**T808S**	DII (S2)	Missense	ICEGTC	Rare sharp waves in left temporal (EEG) Increase current density Delay recovery from inactivation	(Fujiwara, 2003; Rhodes et al., 2005)
**S843X**	DII (S3)	Nonsense	DS	Focal spike activity (EEG)	(Buoni et al., 2006)
**R862G**	DII (S4)	Missense	MMPSI	Multifocal epilepsy Hemiclonic Cardiac arrest Severe intellectual disability	(Carranza Rojo et al., 2011; Barba et al., 2014)
**T932X**	DII (S5-S6)	Nonsense	SME DS	Generalized tonic-clonic seizures Severe mental retardation	(Claes et al., 2003; Dhamija et al., 2014)
**M934I**	DII (S5-S6)	Missense	DS	Moderate psychomotor retardation	(Fukuma et al., 2004; Depienne et al., 2008; Wang et al., 2012)
**H939Q**	DII (S5-S6)	Missense	DS	Status epilepticus Generalized tonic-clonic seizures Complex partial seizures No measurable current	(Claes et al., 2003; Ohmori et al., 2006)
**R946C**	DII (S5-S6)	Missense	SME DS SMEB	Non- functional Channel	(Fukuma et al., 2004; Volkers et al., 2011; Zuberi et al., 2011; Wang et al., 2012; Lee et al., 2014; Xu et al., 2014; Lindy et al., 2018)
**R946S**	DII (S5-S6)	Missense	Severe idiopathic generalized epilepsy of infancy	Short generalized tonic-clonic seizures at night Seizure onset left temporo-parietal (EEG) Seizure onset left frontal Seizure onset right frontocentral,	(Ebach et al., 2005; Tiefes et al., 2019)
**R946H**	DII (S5-S6)	Missense	PEFS+ SMEB DS	Non-functional Channel	(Fukuma et al., 2004; Harkin et al., 2007; Depienne et al., 2008; Liao et al., 2010a; Verbeek et al., 2011; Volkers et al., 2011; Zuberi et al., 2011; Wang et al., 2012; Verbeek et al., 2013)
**C959R**	DII (S5-S6)	Missense	DS	Post trauma epilepsy Lateralized tonic-clonic seizures Severe mental retardation Non-functional Channel	(Claes et al., 2003; Ohmori et al., 2006)
**V971L**	DII (S6)	Missense	DS	Generalized and unilateral tonic-clonic seizures Myoclonic seizures Apneic spells	(Poryo et al., 2017)
**V982L**	DII (S6)	Missense	SMEB	Focal epilepsy	(Singh et al., 2009; Saitoh et al., 2012; Saitoh et al., 2015a; Saitoh et al., 2015b)
**V983A**	DII (S6)	Missense	ICEGTC	Multifocal spikes, high voltage slow-waves (EEG) Reduced current density Shift steady-state inactivation to more positive values Accelerated recovery from inactivation	(Fujiwara, 2003; Rhodes et al., 2005)
**V983AfsX2**	DII (S6)	FrameShift	DS	Enlarged extracerebral gap (MRI)	(Wang et al., 2017b)
**L986F**	DII (S6)	Missense	DS	Generalized tonic-clonic seizures Non-functional channel	(Claes et al., 2001; Lossin et al., 2003)
**L991VfsX2**	DII (S6)	FrameShift	DS	Febrile, partial, generalized tonic-clonic, myo-clonic seizures Moderate intellectual disability.	(Kwong et al., 2012)
**N1011I**	DII-DIII	Missense	ICEGTC	Rare sharp waves in lateral-temporal (EEG) Reduced current density Shift steady state inactivation to more negative values	(Fujiwara, 2003; Rhodes et al., 2005)
**D1046MfsX9**	DII-DIII	FrameShift	DS	Diffuse cerebral edema (Computed tomography)	(Myers et al., 2017b)
**S1100KfsX8**	DII-DIII	FrameShift	DS	Generalized clonic seizures Severe mental retardation	(Claes et al., 2001)
**S1104X**	DII-DIII	Missense	DS	Febrile seizures	(Depienne et al., 2008; Hernández Chávez et al., 2014)
**E1153X**	DII-DIII	Nonsense	DS	Focal epilepsy with frontal-lateral activity (EEG)	(Hernández Chávez et al., 2014)
**E1176NfsX32**	DII-DIII	FrameShift	DS	Severe intellectual disability Intractable seizures despite multiple anti-epileptic drugs	(Willemsen et al., 2012)
**R1213X**	DII-DIII	Nonsense	SME DS LGS	Rare spikes, multifocal spikes and spike-wave complex (EEG) Severe mental delay	(Fujiwara, 2003; Depienne et al., 2008; Zuberi et al., 2011; Wang et al., 2012; Allen et al., 2013; Xu et al., 2014; Lindy et al., 2018)
**L1230P**	DIII (S1)	Missense	DS	Focal spike-wave complex (EEG) Febrile seizures Myoclonic seizures	(Liu et al., 2018)
**F1263L**	DIII (S2)	Missense	SMEB	Rare spike-wave complex and poly spike-waves complex (EEG)	(Fujiwara, 2003)
**R1636Q**	DIV (S4)	Missense	DS LGS	Epileptic encephalopathy Myoclonic seizures	(Harkin et al., 2007; Butler et al., 2017b)
**V1637E**	DIV (S4)	Missense	DS	Episodes of status epilepticus triggered by fever	(Nishri et al., 2010; Zuberi et al., 2011)
**F1671fsX8**	DIV (S4-S5)	FrameShift	DS	Generalized tonic-clonic seizures Severe mental retardation	(Claes et al., 2001; Sugawara et al., 2002; Depienne et al., 2008; Riva et al., 2009)
**A1685D**	DIV (S5)	Missense	DS	Spike-wave complex (EEG) Non-functional channel	(Fujiwara, 2003) (Sugiura et al., 2012)
**Y1694C**	DIV (S5)	Missense	DS	Myoclonic seizures Atypical absence Severe psychomotor retardation	(Fukuma et al., 2004; Wang et al., 2012; Cetica et al., 2017)
**L1717P**	DIV (S5-S6)	Missense	SME	Generalized tonic clonic seizure	(Verbeek et al., 2013)
**T1722A**	DIV (S5-S6)	Missense	DS	Myoclonic, hemiclonic, focal seizures	(Wu et al., 2015)
**C1741S**	DIV (S5-S6)	Missense	TLE-MTS	Febrile status epilepticus	(Tiefes et al., 2019)
**G1754R**	DIV (S5-S6)	Missense	DS	Focal seizures Hemiconvulsions	(Petrelli et al., 2012)
**S1768R**	DIV (S6)	Missense	DS	Absences and tonic-clonic seizures	(Willemsen et al., 2012)
**E1881X**	C-terminal	Nonsense	DS SMEB	Febrile and generalized seizures	(Villeneuve et al., 2014)
Non genetic origin mutations reported*					
**G177DfsX4**	DI (S2-S3)	FrameShift	DS	Generalized tonic-clonic seizures	(Fujiwara, 2003)
**V207G**	DI (S3)	Missense	EE	Early-onset multifocal seizures	(Daoud et al., 2016)
**D249E**	DI (S4-S5)	Missense	DS	Generalized tonic seizures Absences; Mental retardation	(Le Gal et al., 2014)
**N275K**	DI (S5)	Missense	PEFS+	Hippocampal volume loss (MRI)	(Kim et al., 2014)
**T363R**	DI (S5-S6)	Missense	DS	Generalized tonic-clonic seizures	(Zuberi et al., 2011; Le Gal et al., 2014)
**N416I**	DI (S6)	Missense	DS	Focal spike-wave (EEG)	(Zhou et al., 2018)
**S1631C**	DIV (S3-S4)	Missense	DS	Multifocal spikes (EEG)	(Haginoya et al., 2018)

*Non genetic origin mutations reported: Mutations described through clinical diagnosis, but the mutation type (Mendelian or de novo) were not reported, mainly due to the lack of parents to perform genotyping and difficulty in contacting the family. Generalized epilepsy with febrile seizures plus (GEFS+); Febrile seizures (FS); Febrile seizures plus (FS+); Lennox-Gastaut syndrome (LGS); Dravet syndrome (DS); Borderline severe myoclonic epilepsy (SMEB); Severe myoclonic epilepsy (SME); Familial hemiplegic migraine (FHM); Partial epilepsy with antecedent FS (PEFS+); Intractable childhood epilepsy with generalized tonic–clonic seizures (ICEGTC); Intractable childhood epilepsy with generalized tonic-clonic seizures (ICE-GTC); Epileptic encephalopathy (EE); Malignant migrating partial seizures of infancy (MMPSI); Temporal lobe epilepsy (TLE); Mesial temporal sclerosis (MTS); Not Reported (NR); Domain (D); Segment (S); Electroencephalography (EEG); Magnetic resonance imaging (MRI).

Mutations in the NaV1.1 channel are described in almost all regions of the protein and may cause GoF or LoF (Goldin and Escayg, 2010; Meng et al., 2015). Among the 52 mutations in *SCN1A* related to epilepsy with functional studies, 35 mutations (67.30%) exclusively display characteristics of LoF, 6 mutations (11.53%) display characteristics unique to GoF, and 11 mutations (21,15%) display characteristics of GoF+LoF, whereas, in GoF+LoF mutations, the main characteristic that gives GoF features is enhanced persistent current, present in 10 out of the 11 GoF+LoF mutations listed (**Tables 1** and **S1**).

Due to the role of the NaV1.1 channels in the regulation of electrical excitability by the inhibitory interneurons, prescription of AEDs non-selective sodium channel blockers (SCB) for SMEI or GEFS + syndromes is contraindicated, for it may aggravate crises due to the enhanced suppress status of the NaV1.1 channels (Catterall, 2014a; Shi et al., 2016; Knupp and Wirrell, 2018; Ziobro et al., 2018). The first-line drug-based therapy for *SCN1A* epilepsy diseases is the enhancement of postsynaptic GABAergic transmission with allosteric activation of GABA_A_ receptors as target by Clobazam and/or an increase in GABA concentration in synaptic cleft resulting from increased GABA production and decreased GABA degradation as target by Valproic acid (Catterall, 2014a; Hammer et al., 2016; Knupp and Wirrell, 2018; Musto et al., 2020). Antisense nucleotides (ASO) therapy to increase mRNA of *SCN1A* for NaV1.1 channel expression in normal levels is a promising strategy for genetic disorders involving haploinsufficiency (Hsiao et al., 2016; Stoke Therapeutics, 2018). Drug-resistant Dravet syndrome cases may thrive on alternative therapeutic strategies based on ketogenic diets (Nabbout et al., 2011; Wu et al., 2018). A recent study with 20 patients with medically intractable Dravet syndrome caused by missense, non-sense, insertion, deletions and splicing mutations presents efficacy during three months of treatment in 17 patients, decreasing seizure frequency in more than 50% (Yan et al., 2018). Besisdes that, Epidiolex is an FDA approved CBD-based drug approved in June 2018 for the treatment of severe forms of epilepsy, as Dravet and Lennox-Gastaut syndromes (U.S. Food and Drug Administration [website]., 2018). Clinical trials using CBD in DS and LGS shown reduced frequency of seizures in monthly average (Lattanzi et al., 2020; Morano et al., 2020). Voltage-gated sodium channel are inhibit by CBD in low micromolar concentrations, IC_50_ between 1.9 and 3.8 μM, NaV1.4 and NaV1.1 being the most sensitive channels to CBD, 1.9 and 2.0 μM respectively, probably the mechanism of action is reducing channel availability due shift to more hyperpolarized potential in steady-state inactivation (Ghovanloo et al., 2019).

### NaV1.2

NaV1.2 is encoded by the *SCN2A* gene (Wolff et al., 2017). It is located on chromosome 2q24.3 (Shi et al., 2009) and expressed in the CNS (Catterall, 2014a), especially in excitatory neurons (Syrbe et al., 2016) and glutamatergic neurons (Sanders et al., 2018), unlike the NaV1.1 channel, which is highly expressed in the GABAergic interneurons (Catterall, 2014a).

More than 100 mutations have already been described for this gene, with approximately 300 patients studied yet (Reynolds et al., 2020) (**Table 2**). The most common diseases related with *SCN2A* mutation are West syndrome (WS; OMIM #308350), epilepsy of infancy with migrating focal seizures (EIMFS; OMIM #616645), and benign familial neonatal-infantile seizures (BFNIS; OMIM #607745) (Perucca and Perucca, 2019). Although epilepsy-related mutations are present throughout the channel, several hotspots such as the ion selectivity filter, the voltage-sensing domain, the intracellular N-terminal, and the C-terminal domain can be highlighted (Sanders et al., 2018).

**Table 2:** SCN2A-related epilepsies identified in clinical patients through WES and/or NGS.

Variant	Location	Mutation	Disease	Alteration on *biophysical properties* or/and Clinical report	Reference
**Inherited mutation**					
**R19K**	N-terminal	Missense	FS+	Febrile seizures Partial seizure with eye deviation	(Ito et al., 2004)
**R36G**	N-terminal	Missense	BFIS	Focal seizures Clonic seizures	(Wolff et al., 2017)
**I172V**	DI (S2)	Missense	FS	Fever-induced seizure susceptibility	(Saitoh et al., 2015a)
**R188W**	DI	Missense	FS+	Generalized tonic or tonic clonic seizures Partial seizures	(Ito et al., 2004)
**A202V**	DI	Missense	BFNS	Focal seizures Generalized tonic-clonic seizures	(Wolff et al., 2017)
**V208E**	DI	Missense	BFIS	NR	(Lemke et al., 2012)
**R223Q**	DI (S4)	Missense	BFNIS	Positive shifts of both activation and inactivation curves	(Berkovic et al., 2004; Scalmani et al., 2006; Zara et al., 2013)
**D322N**	DI (S5-S6)	Missense	DS	NR	(Shi et al., 2009)
**F328V**	DI (S5-S6)	Missense	SMEB	Status epilepticus Focal seizures Lesions in the right parietal, temporal and occipital lobes (MRI)	(Shi et al., 2009; Saitoh et al., 2015a)
**Q383E**	DI	Missense	BFNIS	Seizures in early infancy	(Syrbe et al., 2016)
**E430Q**	DI-DII	Missense	BFNIS	Focal spikes and bifrontal slow wave activity (EEG)	(Herlenius et al., 2007)
**A467T**	DI-DII	Missense	GEFS+	Loss of consciousness Clonic movements of all extremities High body temperature up to 40 ° Celsiu**s**	(Liu et al., 2018)
**R524Q**	DI-DII	Missense	FS	Febrile seizures	(Ito et al., 2004)
**V892I**	DII (S5)	Missense	BFNIS	NR	(Berkovic et al., 2004)
**N1001K**	DII-DIII	Missense	BFIS	Afebrile seizures Tonic body extension Right parietal–occipital sharp waves (EEG)	(Striano et al., 2006)
**L1003I**	DII-DIII	Missense	BFNIS	Generalized tonic-clonic seizures	(Berkovic et al., 2004)
**R1319Q**	DIII (S4)	Missense	BFNIS	Shift steady state activation and inactivation to more positive values	(Berkovic et al., 2004; Scalmani et al., 2006; Misra et al., 2008; Zara et al., 2013)
**E1321K**	DIII	Missense	BFNS	NR	(Grinton et al., 2015)
**L1330F**	DIII (S4–S5)	Missense	BFNIS	Shift steady state inactivation to more positive values	(Heron et al., 2002; Scalmani et al., 2006; Misra et al., 2008)
**L1563V**	DIV	Missense	BFNIS	Increase in neuronal excitability Accelerated recovery from fast inactivation	(Heron et al., 2002; Scalmani et al., 2006; Xu et al., 2007; Misra et al., 2008; Berecki et al., 2018)
**Y1589C**	DIV (S2-S3)	Missense	BFNIS	Increased persistent Na^+^ current Delayed fast inactivation Acceleration of recovery	(Lauxmann et al., 2013)
**I1596S**	DIV (S3)	Missense	BFNIS	Central and posterior focal spikes (EEG)	(Herlenius et al., 2007)
**K1641N**	DIV	Missense	BFIS	Focal seizures with secondary generalization	(Zara et al., 2013)
***De novo* mutation**					
**R102X** **(Mutation expressed with wild type channel)**	N-terminal	Nonsense	EE	Shift steady state inactivation to more negative values Decrease of available channel	(Kamiya, 2004; Ogiwara et al., 2009)
**N132K**	DI	Missense	EOEE	Tonic-clonic seizures	(Matalon et al., 2014)
**M136I**	DI	Missense	EIMFS	Focal seizures Spasms	(Carvill et al., 2013; Howell et al., 2015)
**E169G**	DI (S2)	Missense	EOEE	Multifocal spikes (EEG) Febrile seizure Myoclonic seizure Focal seizure	(Nakamura et al., 2013)
**W191C**	DI	Missense	EIMFS	Frequent multifocal spikes (EEG)	(Su et al., 2018)
**F207S**	DI	Missense	BNS	Tonic-clonic seizures Clonic seizures	(Wolff et al., 2017)
**G211D**	DI	Missense	WS	NR	(Kodera et al., 2013)
**N212D**	DI (S3-S4)	Missense	OS and WS	Eyelid myoclonic Spasms Hypsarrhythmia	(Nakamura et al., 2013)
**R220G**	DI	Missense	EE	Generalized tonic-clonic seizures Generalized spike and slow wave (EEG)	(Mercimek-Mahmutoglu et al., 2015)
**T227I**	DI	Missense	WS	Tonic seizures Apneic seizures Spasms	(Wolff et al., 2017)
**T236S**	DI (S4-S5)	Missense	OS	Focal seizure	(Nakamura et al., 2013)
**A240S**	DI	Missense	EIMFS	Focal seizures	(Howell et al., 2015)
**M252V**	DI (S5)	Missense	BFNIS	Increased persistent current Accelerated of recovery from fast inactivation Accelerated of recovery from slow inactivation	(Liao et al., 2010b)
**V261M**	DI (S5)	Missense	BFNIS	Enhanced persistent current Faster recovery from inactivation	(Liao et al., 2010b)
**A263T**	DI (S5)	Missense	EOEE	Multifocal spikes (EEG)	(Nakamura et al., 2013)
**V423L**	DI (S6)	Missense	OS	Change in slope of steady-state activation curve Enhanced persistent current	(Wolff et al., 2017)
**E430G**	DI-DII	Missense	OS	Generalized tonic-clonic seizures	(Matalon et al., 2014)
E717G.fs*30	DI-DII	Splice site	EE Cerebral and cerebellar atrophy	High amplitude sharp waves (EEG)	(Horvath et al., 2016)
**G828V**	DII	Missense	BNS	Focal seizures Clonic seizures Autonomic seizures Tonic-clonic seizures Multifocal spikes (EEG)	(Wolff et al., 2017)
**R853Q**	DII (S4)	Missense	WS	Reduced transient current amplitude and densityShift steady state inactivation to more negative values Decreased persistent current	(Samanta and Ramakrishnaiah, 2015; Wolff et al., 2017; Berecki et al., 2018; Mason et al., 2019)
**R856L**	DII	Missense (During embryogenesis)	EIMFS	Focal seizures	(Howell et al., 2015)
**R856Q**	DII	Missense	OS	Tonic seizures	(Wolff et al., 2017)
**S863F**	DII	Missense	BNS and Focal epilepsy	Generalized tonic-clonic seizures	(Wolff et al., 2017)
**I873M**	DII	Missense	EIEE	Abnormal electroretinogram	(Trump et al., 2016)
**N876T**	DII (S4-S5)	Missense	OS and WS	Spasms Focal seizure	(Nakamura et al., 2013)
**L881P**	DII	Missense	WS and LGS	Tonic seizures Tonic-clonic seizures Atypical absences	(Wolff et al., 2017)
**G882R**	DII	Missense	EIMFS	Unilateral tonic-clonic	(Wolff et al., 2017)
**G882E**	DII	Missense	EIMFS	Focal seizures Autonomic seizures Hemiclonic seizures Myoclonic seizures Clonic seizures	(Wolff et al., 2017)
**V887A**	DII	Missense	OS	Spasms	(Wolff et al., 2017)
**G899S**	DII (S5)	Missense	Intractable infantile Childhood epilepsy	Tonic-clonic seizures and absences Shift steady-state activation to more positive values Increased slop factor	(Wolff et al., 2017)
**K905N**	DII	Missense	EIMFS	Focal seizures	(Carvill et al., 2013; Howell et al., 2015)
**F928C**	DII	Missense	EIMFS	Focal seizures	(Carvill et al., 2013; Howell et al., 2015)
**H930Q**	DII	Missense	MAE	Tonic-clonic seizures Atonic seizures Myoclonic-atonic seizures Tonic seizures Atypical absences	(Wolff et al., 2017)
**N976K**	DII	Missense	EE	Focal seizures	(Howell et al., 2015)
**S987I**	DII	Missense	EIEE	Focal and tonic seizures	(Trump et al., 2016)
**G999L**	DII-DIII	Missense	Infantile epilepsy	Diffuse slowing with high-amplitude bursts of activity (EEG) Generalized seizures with burst suppression	(Foster et al., 2017)
**E999K**	DII-DIII	Missense	EIEE	NR	(Trump et al., 2016)
**E999V**	DII-DIII	Missense	EIEE OS	NR	(Allen et al., 2016; Trump et al., 2016)
I1021Y.fs*16	DII-DIII	Frameshift	LGS	NR	(Carvill et al., 2013)
**E1211K**	DIII (S1)	Missense	WS	Shift steady-state activation and inactivation to more negative values Slower recovery from inactivation	(Ogiwara et al., 2009; Wong et al., 2015)
**K1260E and K1260Q (Mosaic)**	DIII	Missense	EIEE	NR	(Trump et al., 2016)
**R1312T**	DIII (S4)	Missense	DS	Reduced current density Shift steady-state activation and inactivation to more negative values Enhanced closed-state inactivation Slowed recovery from inactivation	(Shi et al., 2009; Lossin et al., 2012)
**M1323V**	DIII (S4-S5)	Missense	OS and WS	Multifocal spikes (EEG)	(Nakamura et al., 2013)
**V1326D**	DIII	Missense	EIMFS	Focal seizures	(Dhamija et al., 2013)
**S1336Y**	DIII (S4-S5)	Missense	OS and WS	Modified hypsarrhythmia	(Nakamura et al., 2013)
**M1338T**	DIII (S4-S5)	Missense	OS	Spasms Focal seizure Multifocal spikes (EEG)	(Nakamura et al., 2013)
**L1342P**	DIII	Missense	IOEE	Progressive brain atrophy Short tonic seizures Multifocal sharp wave activity (EEG)	(Hackenberg et al., 2014)
**I1473M**	DIII (S6)	Missense	SNEE	Shift steady-state inactivation to more negative values	(Ogiwara et al., 2009)
**Q1479P**	DIII	Missense	EIEE	NR	(Trump et al., 2016)
V1528Cfs*7	DIII-DIV	Frameshift	LGS	Tonic-clonic seizures Tonic seizures Status epilepticus	(Wolff et al., 2017)
**Q1531K**	DIII-DIV	Missense	BNS	Clonic seizures Generalized tonic-clonic seizures	(Wolff et al., 2017)
**I1537S and M1538I**	DIV	Missense	OS and WS	Clonic seizures Frequent seizure activity (EEG)	(Foster et al., 2017)
**M1548V**	DIV	Missense	OS and WS	Generalized tonic-clonic seizures	(Wolff et al., 2017)
**G1593R**	DIV	Missense	EIMFS	Focal seizures	(Howell et al., 2015)
**F1597L**	DIV (S3)	Missense	EIMFS	Shift steady-state activation to more negative values accelerated recovery from fast inactivation	(Wolff et al., 2017)
**D1598G**	DIV (S3)	Missense	SME	Severe intellectual disability Developmental delay Seizures/ infantile spasms	(Need et al., 2012)
**P1622S**	DIV (S3-S4)	Missense	MAE	Shift steady-state inactivation to more negative values	(Wolff et al., 2017)
**T1623N**	DIV (S3-S4)	Missense	OS and WS	Multifocal spikes (EEG) Spasms Hypsarrhythmia	(Nakamura et al., 2013)
**V1627M**	DIV	Missense	EIMFS	Focal seizures Apnoeic seizures	(Wolff et al., 2017)
**G1634V**	DIV	Missense	OS	Focal seizures Spasms	(Howell et al., 2015)
**I1640S**	DIV	Missense	EE	Tonic seizures Focal seizues	(Wolff et al., 2017)
**L1650P**	DIV	Missense	EIEE	NR	(Trump et al., 2016)
**A1652P**	DIV	Missense	WS	Spasms	(Wolff et al., 2017)
**S1656F**	DIV	Missense	LGS	Generalized tonic-clonic seizures	(Wolff et al., 2017)
**L1660T**	DIV (S4-S5)	Missense	EE	Generalized tonic-clonic seizures	(Fukasawa et al., 2015)
**L1660W**	DIV	Missense	Acute encephalopathy	Tonic-clonic convulsions Frequent spikes and sharp waves in the right fronto-temporal regions (EEG) Cerebellar atrophy (MRI)	(Fukasawa et al., 2015)
**Q1811E**	C-terminal	Missense	OS	Generalized tonic-clonic seizures Focal seizures	(Wolff et al., 2017)
**L1829F**	C-terminal	Missense	EIEE	NR	(Trump et al., 2016)
**H1853R**	C-terminal	Missense	OS	Generalized tonic-clonic seizures Absence seizures	(Martin et al., 2014)
**R1882L**	C-terminal	Missense	Epilepsy	Generalized and irregular spike wave and polyspike wave activity (EEG) Focal and generalized tonic–clonic seizures with opisthotonus, bradycardia, and cyanosis	(Baasch et al., 2014)
**R1882G**	C-terminal	Missense	BIS	Shift steady-state inactivation to more positive values Increase current density and protein production	(Carvill et al., 2013; Schwarz et al., 2016; Wolff et al., 2017)
**R1882Q**	C-terminal	Missense	EIEE	Increased current density Enhanced persistent current	(Trump et al., 2016; Berecki et al., 2018; Mason et al., 2019)
**D25Nβ1** β1 subunit mutation*	β subunit	Substitution * human embryonic kidney 293 (HEK) cells co-expressing human Nav1.2 sodium channels and D25Nβ1	GEFS+	Inhibits the increment of functional expression of NaCh currents Abolishes the shift of the voltage dependence of activation and inactivation	(Baroni et al., 2018)
**Chromosome 2q24.3** **Portions of the *SCN2A* and *SCN3A* genes**	Chromosome	Deletion (112-kb)	Mental retardation Infantile seizures	Anxiety disorders ‘shiver-like’ episodes	(Bartnik et al., 2011)
**Chromosome q24.3q31.1** **58 known genes including *SCN2A, SCN1A, SCN3A, SCN9A* and *SCN7A***	Chromosome	Deletion (10.29 - 10.58 Mb)	Severe epilepsy	Focal and generalized seizures Stereotypic and repetitive hand movements Slow background with high amplitude delta waves mixed with spikes and sharp waves on the temporo-occipital areas (EEG)	(Pescucci et al., 2007)
Non genetic origin mutations reported*					
**V213D**	DI (S4)	Missense	EOEE	Focal seizure Focal spikes (EEG)	(Nakamura et al., 2013)
**T218K**	DI	Missense	EIMFS	Focal seizures Spasms	(Howell et al., 2015)
**D649N**	DI-DII	Missense	DS	NR	(Wang et al., 2012)
**V752F**	DI-DII	Missense	Absence epilepsy	Increased current density Shift steady-state activation and inactivation to more negative values	(Oliva et al., 2014)
**M1128T**	DII-DIII	Missense	AERRPS	Generalized convulsive seizure Slow background activity and rare multifocal spikes over the right temporal and bilateral frontopolar regions (EEG) Brain edema (Cranial computed tomography)	(Kobayashi et al., 2012)
**G1522A**	DIII-DIV	Missense	EE	Absence seizures Generalized spike and waves (EEG)	(Mercimek-Mahmutoglu et al., 2015)
**R1629L**	DIV (S4)	Missense	EOEE	Focal seizure Burst of spikes (EEG)	(Nakamura et al., 2013)
**R1918H**	C-terminus	Missense	GEFS+	Generalized tonic-clonic seizures	(Haug et al., 2001)
**GAL879-881QQQ**	DII (S4-S5) (rat brain)	Mutated channel in transgenic mice	Epilepsy	Delayed fast inactivation Increased persistent current when expressed in Xenopus oocytes	(Kearney et al., 2001)
**R85Cβ1**	Extracellular immunoglobulin-like domain (β1 subunit)	Substitution *Human embryonic kidney (HEK)-293T cells co-expressing human brain NaV1.2 alpha subunit and R85Cβ1	GEFS+	Fail to modulate fast inactivation kinetics Fail to modulated steady-state inactivation	(Xu et al., 2007)
**R85Hβ1**	Extracellular immunoglobulin-like domain (β1 subunit)	Substitution *Human embryonic kidney (HEK)-293T cells co-expressing human brain NaV1.2 alpha subunit and R85Hβ1	GEFS+	Fail to modulated fast inactivation kinetics	(Xu et al., 2007)
**C121Wβ1** β1 subunit mutation*	Ig-like domain (β1 subunit)	Substitution * Chinese hamster ovary (CHO) cells co-expressing human Nav1.2 sodium channels and C121Wβ1	GEFS+	Destabilization of steady-state inactivation potentials Disrupts the thermoprotective role of the β1 subunit on channel availability	(Egri et al., 2012; Abdelsayed and Sokolov, 2013)
**Chromosome 2q24.3** **Involves the *SCN2A* and *SCN3A* genes**	Chromosome	Duplication (1.77 Mb)	EOEE	Multifocal spikes (EEG) Epileptic spasms	(Baumer et al., 2015)
**Chromosome 2q24.3- q31.1** **47 genes involved including *SCN1A, SCN2A, SCN3A, SCN7A* and *SCN9A***	Chromosome	Deletion (10.4-Mb)	Severe epilepsy	Epileptic seizure with pale, atonic periods followed by a spasm-like out-throwing of both arms Predominantly right-sided epileptiform activity (EEG)	(Davidsson et al., 2008)

*Non genetic origin mutations reported: Mutations described through clinical diagnosis, but the mutation type (Mendelian or de novo) were not reported, mainly due to the lack of parents to perform genotyping and difficulty in contacting the family. Generalized epilepsy with febrile seizures plus (GEFS+); Benign familial neonatal-infantile seizures (BFNIS); Benign familial neonatal seizures (BFNS); Benign Familial Infantile Seizures (BFIS); Benign neonatal/infantile seizures (BNIS); Benign neonatal seizures (BNS); Benign infantile seizures (BIS); Febrile seizures (FS); Febrile seizures plus (FS+); Epilepsy of infancy with migrating focal seizures (EIMFS); Ohtahara syndrome (OS); West syndrome (WS); Lennox-Gastaut syndrome (LGS); Dravet syndrome (DS); Borderline severe myoclonic epilepsy (SMEB); Severe myoclonic epilepsy (SME); Early-onset epileptic encephalopathies (EOEE); Acute encephalitis with refractory, repetitive partial seizures (AERRPS); Early infantile epileptic encephalopathy (EIEE); myoclonic-atonic epilepsy; Infantile onset epileptic encephalopathy (IOEE); Sporadic neonatal epileptic encephalopathy (SNEE); Epileptic encephalopathy (EE); Not Reported (NR); Domain (D); Segment (S); Electroencephalography (EEG); Magnetic resonance imaging (MRI).

NaV1.2 channels are expressed in the excitatory neurons; therefore, GoF mutations are related to epilepsy because it causes neuronal hyperexcitability. On the other hand, LoF mutations are related to autism and intellectual disability phenotype (Ben-Shalom et al., 2017). Nevertheless, some studies have already related loss of function to epilepsy, as described by Lossin and co-workers (2012) with *R1312T* mutation (Lossin et al., 2012). Normally, LoF SCN2A gene mutations for epilepsy are related to late-onset epilepsy; however, the mechanism of action is unclear (Mason et al., 2019).

In some cases, NaV1.2 seizures are not controlled not even by various antiepileptic drugs, as with the patient described by Syrbe and colleagues (2016). The proband, even after being treated with oxcarbazepine (OXC), valproic acid, topiramate, sulthiame, phenytoin, among other drugs, kept on having seizures (Syrbe et al., 2016). Furthermore, the SCB drugs can assist the patient during the treatment as described by Gorman and King (2017). The patient had seizures controlled after administration of phenytoin (Gorman and King, 2017). In addition, Musto et al. (2020) cite benefits treatments using SCB such as carbamazepine, mexiletine, oxcarbazepine, phenytoin, lidocaine, and lamotrigine for patients with early onset epilepsies (Musto et al., 2020). Besides, Peters and colleagues studied a substance commercially used as an antianginal drug (human heart) called ranolazine that has been shown to affect NaV1.2 channels, reducing macroscopic currents and delaying the recovery of fast and slow inactivation of the NaV1.2 channel, consequently with more future studies ranolazine could be a efficacious therapy for epilepsy (Peters et al., 2013).

Drugs can be important to modulate channel kinetics for both GoF and LoF, but some precautions must be observed. For example, the degree of conservation between subtypes, such as NaV1.2 and other sodium channels as NaV1.5 and the excessive decrease in channel function or the excessive increase in function obtained by the drug (Sanders et al., 2018).

Organizations like the FamilieSCN2A Foundation (www.scn2a.org) might be essential in the search for new treatments. Understanding the genotype-phenotype of gain and loss of function is essential because science-patient relationship may be helpful in the search for new therapies (Sanders et al., 2018).

### NaV1.3

*SCN3A* is a gene that encodes for type 3 voltage-gated Na^+^ channel α subunit, the NaV1.3, located on human chromosome 2q24, in a cluster with *SCN1A* and *SCN2A* (Holland et al., 2008). NaV1.3 is expressed predominantly in the CNS during embryonic and neonatal development, being extremely low or sometimes undetectable in postnatal individuals. Subsequently, during infancy, it is gradually replaced by increased expression of the NaV1.1 isoform (Felts et al., 1997; Whitaker et al., 2000; Cheah et al., 2013; Zaman et al., 2018). On the other hand, studies regarding nervous system injury and neuropathic pain showed an increasing presence of NaV1.3 channels in affected tissues, suggesting a pivotal hole of these transmembrane proteins in these processes and diseases (Hains et al., 2003; Waxman and Hains, 2006; Black et al., 2008). For the reasons mentioned above, in the last decades, NaV1.3-associated pathogenesis has been restricted to pain. Recently, a genetic linkage between NaV1.3 mutated variants and epilepsy has been suggested, especially in cryptogenic epilepsy cases (OMIM#182391).

K354Q was the first described NaV1.3 epilepsy-related mutation that revealed harmful electrophysiological alterations (Holland et al., 2008; Estacion et al., 2010). In fact, mutations can change many functional characteristics of NaV1.3 affecting biophysical properties differently; however, these changes result predominantly in neuronal hyper-responsiveness (**Table 3**) (Cummins and Waxman, 1997; Chen et al., 2000; Cummins et al., 2001; Sun et al., 2007). Previous reports correlate heterozygous variants in *SCN3A* in association with moderate forms of epilepsy, while homozygosis is related with severe cognitive damage and premature mortality, resulting in a broad range of epileptic phenotypes (Estacion and Waxman, 2013; Vanoye et al., 2014; Lamar et al., 2017).

**Table 3:** SCN3A-related epilepsies identified in clinical patients through WES and/or NGS.

Variant	Location	Mutation	Disease	Alteration on *biophysical properties* or/and Clinical report	Reference
**Inherited mutation**					
**K354Q**	DI	Missense	CCE	Enhanced persistent current and current amplitude provokes by ramp protocol	(Holland et al., 2008; Estacion et al., 2010)
**R357Q**	DI (S5-S6)	Missense	Focal epilepsy	Reduced current density Enhanced current amplitude provokes by ramp voltage protocol	(Vanoye et al., 2014)
**R621C**	DI-DII	Missense	BECTS FS	Centro-temporal spikes (EEG)	(Zaman et al., 2018)
**E1111K**	DII-III	Missense	Focal epilepsy	Enhanced current amplitude provokes by ramp voltage protocol Enhanced persistent current	(Vanoye et al., 2014)
**M1323V**	DIII (S5-S6)	Missense	Focal epilepsy	Enhanced current amplitude provokes by ramp voltage protocol	(Vanoye et al., 2014)
**C121Wβ1** β1 subunit mutation*	Extracellular Ig loop	Substitution * Chinese hamster ovary (CHO) cells co-expressing human Nav1.3 sodium channels and C121Wβ1	GEFS+	Resistant to enter into close-state inactivation Shift steady state inacativation to more positive values	(Lucas et al., 2005)
**Chromosome 2q24.3** **Involves the *SCN1A,SCN2A*, and *SCN3A* genes**	Chromosome	Duplication (1.57 Mb)	BFNS	NR	(Heron et al., 2010)
**Chromosome 2q24.3** **Involves the *SCN1A,SCN2A*, and *SCN3A* genes**	Chromosome	Duplication (2.0 Mb)	Neonatal- infantile epilepsy	Facial flushing, head turning to the left, eye deviation, bilateral arm jerking movement	(Raymond et al., 2011)
**Chromosome** **2q23.3q24.3** **Involves the *SCN2A* and *SCN3A* genes**	Chromosome	Mosaic duplication (12 Mb)	DS BFNIS	Focal seizures with secondary generalization Atonic seizures (EEG)	(Vecchi et al., 2011)
***De novo* mutation**					
**L247P**	DI	Missense	Childhood focal epilepsy	Reduced current density associated with low protein expression	(Lamar et al., 2017)
**I875T**	DII (S4-S5)	Missense	EE	Enhanced persistente current Shift steady-state activation and inactivation to more negative values Generalized convulsion, infantile spasm	(Miyatake et al., 2018; Smith et al., 2018; Zaman et al., 2018)
**P1333L**	DIII	Missense	EIEE	Enhanced persistent current Increased current density Shift steady-state activation and inactivation to more negative values	(Trujillano et al., 2017; Zaman et al., 2018)
**M1765I**	DIV	Missense	Refractory epilepsy	Focal and generalized seizures Myoclonus and epileptic spasms	(Inuzuka et al., 2019)
**V1769A**	DIV (S6)	Missense	EIEE	Enhanced persistent current Shift steady-state activation to more negative values Shift steady-state inactivation to more positive values	(Zaman et al., 2018)
**chromosome 2q24.3** **Involves the *SCN1A,SCN2A*, and *SCN3A* genes**	chromosome	Deletion (1.1 Mb)	WS	Typical hypsarrhythmic pattern (sleeping and awake)	(Chong et al., 2018)
Non genetic origin mutations reported*					
**N302S**	DI	Missense	GEFS+	Shift steady-state activation and inactivation to more positive values Slower recovery from inactivation with 500 ms duration pre pulse Faster recovery from inactivation with 20 ms duration pre pulse	(Chen et al., 2015)
**D766N**	DII (S2)	Missense	Focal epilepsy	Increased current amplitude by ramp voltage protocol	(Vanoye et al., 2014)

*Non genetic origin mutations reported: Mutations described through clinical diagnosis, but the mutation type (Mendelian or de novo) were not reported, mainly due to the lack of parents to perform genotyping and difficulty in contacting the family. Cryptogenic childhood epilepsy (CCE); Benign epilepsy with centro-temporal spikes (BECTS); Generalized epilepsy with febrile seizures plus (GEFS+); West syndrome (WS); Febrile seizures (FS); Benign familial neonatal-infantile seizures (BFNIS); Benign familial neonatal seizures (BFNS); Dravet syndrome (DS); Epileptic encephalopathy (EE); Early infantile epileptic encephalopathy (EIEE); Not Reported (NR); Domain (D); Segment (S); Electroencephalography (EEG).

Different hereditary mutations on NaV1.3 have been reported to date in patients with epilepsy. In general, the biophysical characterization of these mutations reveals GoF, only one mutation (N302S) is related with LoF (Chen et al., 2015), but both GoF and LoF may lead to an increased seizure susceptibility (Lamar et al., 2017).

Moreover, several *de novo* mutations in *SCN3A* have been described in the last three years, related with severe infantile neurological dysfunctions and cognitive impairments. These mutations may alter the functionality of NaV1.3 channels, neurons organization, migration, and proliferation during the embryonic development (Smith et al., 2018). Epileptic encephalopathy and polymicrogyria are the main features related with these pathogenic variants, and, so far, polymicrogyria was not reported in other channelopathies, being an exclusive characteristic of *SCN3A* mutants (Inuzuka et al., 2019).

There is a lack of clinical data on *SCN3A*-related epilepsies, especially regarding treatment and the use of specific medication. However, *in vitro* studies reported that mutations related with GoF effect respond favorably to treatment using SCB, like phenytoin, carbamazepine, lacosamide, and topiramate (Sun et al., 2007; Sheets et al., 2008; Colombo et al., 2013; Zaman et al., 2018). The anticonvulsant valproic acid represents a novel and promising epigenetic therapeutic approach (Tan et al., 2017). The compound modulates the *SCN3A* gene through methylation, downregulating the expression of NaV1.3 and, consequently, decreasing biophysical alterations in the channel.

### NaV1.6 

The *SCN8A* gene encodes for type 8 voltage-gated Na^+^ channel α subunit, the NaV1.6, located in chromosome 12q13.13. The first case of *SCN8A* pathogenic variant associated with epilepsy was reported eight years ago (Veeramah et al., 2012). Thereafter, due to advances in genome sequencing technology, especially the WES, the number of epilepsy diagnosis associated with NaV1.6 mutations has increased significantly (OMIM #600702), with more than 300 patients diagnosed with *SCN8A* epilepsy mutations and nearly 200 different putative spots of mutations described, totaling over 100 published reports (**Table 4**). A website developed especially to present *SCN8A* epilepsy and related diseases (www.scn8a.net) was created to provide information to families, clinicians, and researchers, gathering news and recent publications on the subject in a private forum for family interaction, to answer questions, strengthening the ties between the community and the researchers.

**Table 4:** SCN8A-related epilepsies identified in clinical patients through WES and/or NGS.

Variant	Location	Mutation	Alteration on *biophysical properties* or/and Clinical report	Reference
**Inherited mutation**				
**K101R**	N-terminus	Missense	NR	(Butler et al., 2017b)
**I137M**	D1 (S1)	Missense	NR	(Johannesen et al., 2019)
**T164M**	DI (S2)	Missense	NR	(Butler et al., 2017a)
**G269R**	DI (S5)	Missense	Non-functional channel	(Wengert et al., 2019)
**R530W**	DI (S6)-DII (S1)	Missense	NR	(Olson et al., 2015)
N544 fs*39	DI (S6)-DII (S1)	Frameshift	NR	(Johannesen et al., 2019)
**S702T**	DI (S6)-DII (S1)	Missense	NR	(Jang et al., 2019)
**G822R**	DII (S3)	Missense	Non-functional channel	(Wengert et al., 2019)
**V891M**	DII (S5)	Missense	NR	(Johannesen et al., 2019)
**L1290V**	DIII (S3-S4)	Missense	NR	(Carvill et al., 2013)
**L1331V**	DIII (S5)	Missense	NR	(Larsen et al., 2015)
**T1360N**	DIII (S5-S6)	Missense	Shift steady-state inactivation to more negative values	(Wengert et al., 2019)
**E1442K**	DIII (S5-S6)	Missense	NR	(Liu et al., 2018)
**I1464T**	DIII (S6)-DIV (S1)	Missense	NR	(Johannesen et al., 2019)
**G1476D**	DIII (S6)-DIV (S1)	Missense	NR	(Han et al., 2017)
**E1483K**	DIII (S6)-DIV (S1)	Missense	NR	(Gardella et al., 2016)
**I1583T**	DIV (S3)	Missense	NR	(Berghuis et al., 2015)
**V1598A**	DIV (S3)	Missense	NR	(Wang et al., 2017a)
**R1638C**	DIV (S4)	Missense	Shift steady-state activation to more positive values	(Wengert et al., 2019)
**V1758A**	DIV (S6)	Missense	Shift steady-state activation to more positive values	(Zaman et al., 2019)
**N1877S**	C-Terminus	Missense	NR	(Butler et al., 2017b; Johannesen et al., 2019)
**R1904C**	C-Terminus	Missense	NR	(Schreiber et al., 2020)
***De novo* mutation**				
**Exons 2-14**	–	Deletion	NR	(Berghuis et al., 2015)
**c.-8A > G UTR**	5′ UTR	Eight base pairs change upstream of start codon	NR	(Johannesen et al., 2019)
**c.4296A>G**	DIII	Splice-site mutation	NR	(Denis et al., 2019)
**M139I**	D1 (S1)	Missense	Shift steady-state inactivation to more negative values Enhanced persistent current Slightly impaired fast channel inactivation	(Zaman et al., 2019)
**I142V**	D1 (S1)	Missense	NR	(Denis et al., 2019; Kim et al., 2019)
**A205E**	D1 (S1)	Missense	NR	(Lindy et al., 2018)
**F210L**	D1 (S1)	Missense	NR	(Mercimek-Mahmutoglu et al., 2015)
**V211L**	DI (S3)	Missense	NR	(Denis et al., 2019)
**V211A**	DI (S3)	Missense	NR	(Berkovic et al., 2018)
**L213P**	D1 (S3)	Missense	NR	(Denis et al., 2019)
**G214D**	DI (S3-S4)	Missense	NR	(Allen et al., 2013)
**N215R**	DI (S3-S4)	Missense	NR	(Larsen et al., 2015)
**N215D**	DI (S3-S4)	Missense	NR	(Deciphering Developmental Disorders Study, 2015)
**V216D**	DI (S3-S4)	Missense	NR	(Ohba et al., 2014)
**R223G**	D1 (S4)	Missense	Reduced current density Increased current amplitude provokes by ramp voltage protocol	(de Kovel et al., 2014; Berkovic et al., 2018; Denis et al., 2019)
**I231T**	D1 (S4)	Missense	NR	(Berkovic et al., 2018)
**S232P**	D1 (S4)	Missense	NR	(Wang et al., 2017a)
**T239S**	D1 (S4-S5)	Missense	NR	(Møller et al., 2016)
**I240V**	DI (S4-S5)	Missense	NR	(McNally et al., 2016)
**L257V**	DI (S5)	Missense	NR	(Schreiber et al., 2020)
**F260S**	DI (S5)	Missense	NR	(Larsen et al., 2015; Boerma et al., 2016)
**C261F**	DI (S5)	Missense	NR	(Rim et al., 2018; Kim et al., 2019)
**L267S**	DI (S5)	Missense	NR	(Malcolmson et al., 2016)
**G317A**	DI (S5-S6)	Missense	NR	(Denis et al., 2019)
**F360A**	DI (S5-S6)	Missense	NR	(Rolvien et al., 2017)
**M367V**	DI (S5-S6)	Missense	NR	(Lindy et al., 2018)
**N374K**	DI (S5-S6)	Missense	Shift steady-state activation to more negative values	(Johannesen et al., 2019; Zaman et al., 2019)
**T386R**	DI (S5-S6)	Missense	NR	(Lindy et al., 2018)
**Y401H**	DI (S6)	Missense	NR	(Gardella et al., 2018)
**L405M**	DI (S6)	Missense	NR	(Denis et al., 2019)
**L407F**	DI (S6)	Missense	NR	(Fung et al., 2015; Zhang et al., 2015)
**A408T**	DI (S6)	Missense	NR	(Trump et al., 2016; Denis et al., 2019)
**V410L**	DI (S6)	Missense	NR	(Larsen et al., 2015)
**L483F**	DI (S6) –DII (S1)	Missense	Slight shift steady-state activation to more negative values	(Zaman et al., 2019)
**E587Ter**	DI (S6)-DII (S1)	Nonsense	NR	(Schreiber et al., 2020)
**I763V**	DII (S1)	Missense	NR	(Butler et al., 2017b; Hewson et al., 2018; Lindy et al., 2018; Costain et al., 2019; Johannesen et al., 2019)
**T767I**	DII (S1)	Missense	Decreased current density Increased current amplitude provokes by voltage ramp protocol	(Estacion et al., 2014; Gardella et al., 2018; Lindy et al., 2018)
**V791F**	DII (S2)	Missense	NR	(Xie et al., 2019)
**V842E**	DII (S4)	Missense	NR	(Lindy et al., 2018)
**S845F**	DII (S4)	Missense	NR	(Lindy et al., 2018)
**F846S**	DII (S4)	Missense	NR	(Ohba et al., 2014)
**L848W**	DII (S4)	Missense	NR	(Denis et al., 2019)
**R850Q**	DII (S4)	Missense	Shift steady state inactivation to more negative values Increased persistent current Impaired inactivation	(Fung et al., 2015; Zhang et al., 2015; Lindy et al., 2018; Kim et al., 2019; Tsang et al., 2019; Pan and Cummins, 2020; Schreiber et al., 2020)
**R850E**	DII (S4)	Missense	NR	(Wang et al., 2017a)
**R850L**	DII (S4)	Missense	NR	(Gardella et al., 2018)
**L864V**	DII (S4-S5)	Missense	NR	(Gardella et al., 2018)
**L875Q**	DII (S5)	Missense	NR	(Allen et al., 2013)
**A890T**	DII (S5)	Missense	NR	(Fung et al., 2015; Larsen et al., 2015; Zhang et al., 2015)
**V891M**	DII (S5)	Missense	NR	(Wang et al., 2017a)
**V960D**	DII (S6)	Missense	NR	(Larsen et al., 2015)
**L971V**	DII (S6)	Missense	NR	(Kim et al., 2019)
**S978R**	DII (S6)-DIII (S1)	Missense	NR	(Kim et al., 2019)
**S978G**	DII (S6)-DIII (S1)	Missense	NR	(Parrini et al., 2017; Gardella et al., 2018)
**N984K**	DII (S6)-DIII (S1)	Missense	Shift steady-state activation to more negative values	(Blanchard et al., 2015; Boerma et al., 2016)
**G1050S**	DII (S6)-DIII (S1)	Missense	NR	(McMichael et al., 2015)
**S1073N**	DII (S6)-DIII (S1)	Missense	NR	(Lindy et al., 2018)
**E1201K**	DIII (S1)	Missense	NR	(Johannesen et al., 2019)
**V1274M**	DIII (S3)	Missense	NR	(Jang et al., 2019)
**V1315M**	DIII (S4-S5)	Missense	NR	(Trump et al., 2016; Bagnasco et al., 2018; Denis et al., 2019)
**N1318S**	DIII (S4-S5)	Missense	NR	(Johannesen et al., 2019; Lin et al., 2019)
**A1319S**	DIII (S4-S5)	Missense	NR	(Lindy et al., 2018)
**A1319D**	DIII (S4-S5)	Missense	NR	(Johannesen et al., 2019)
**A1323S**	DIII (S4-S5)	Missense	NR	(Trump et al., 2016)
**A1323T**	DIII (S4-S5)	Missense	NR	(Johannesen et al., 2019)
**I1327V**	DIII (S4-S5)	Missense	NR	(Vaher et al., 2013; Singh et al., 2015; Trump et al., 2016)
**N1329D**	DIII (S4-S5)	Missense	NR	(Butler et al., 2017b)
**V1330M**	DIII (S4-S5)	Missense	NR	(Schreiber et al., 2020)
**L1332R**	DIII (S5)	Missense	NR	(Butler et al., 2017b)
**P1428_K1473del**	DIII (S5-S6)	Missense	NR	(Larsen et al., 2015)
**G1451S**	DIII (S6)	Missense	Non-functional channel	(Blanchard et al., 2015; Denis et al., 2019)
**N1466K**	DIII (S6)-DIV (S1)	Missense	NR	(Ohba et al., 2014)
**N1466T**	DIII (S6)-DIV (S1)	Missense	NR	(Ohba et al., 2014)
**Q1470K**	DIII (S6)-DIV (S1)	Missense	NR	(Pons et al., 2018; Denis et al., 2019)
**G1475R**	DIII (S6)-DIV (S1)	Missense	Enhanced persistent current	(Hussain et al., 2016; Ortiz Madinaveitia et al., 2017; Parrini et al., 2017; Wang et al., 2017a; Gardella et al., 2018; Lindy et al., 2018; Xiao et al., 2018; Kim et al., 2019; Trivisano et al., 2019; Zaman et al., 2019; Ranza et al., 2020; Schreiber et al., 2020)
**G1476S**	DIII (S6)-DIV (S1)	Missense	NR	(Lindy et al., 2018)
**I1479V**	DIII (S6)-DIV (S1)	Missense	NR	(Larsen et al., 2015; Lindy et al., 2018; Schreiber et al., 2020)
**E1483K**	DIII (S6)-DIV (S1)	Missense	NR	(Johannesen et al., 2019)
**A1491V**	DIII (S6)-DIV (S1)	Missense	Shift steady-state activation to more negative values Increased current amplitude provokes by slow voltage ramp protocol	(Gardella et al., 2018; Lindy et al., 2018; Zaman et al., 2019)
**M1494T**	DIII (S6)-DIV (S1)	Missense	NR	(Kim et al., 2019)
**K1498M**	DIII (S6)-DIV (S1)	Missense	NR	(Gardella et al., 2018)
**M1529V**	DIV (S1)	Missense	NR	(Johannesen et al., 2019)
**I1532F**	DIV (S1)	Missense	NR	(Møller et al., 2016; Gardella et al., 2018)
**M1536I**	DIV (S1)	Missense	NR	(Lindy et al., 2018)
**F1547V**	DIV (S1-S2)	Missense	NR	(Gardella et al., 2018)
**F1588L**	DIV (S3)	Missense	NR	(Johannesen et al., 2019)
**V1592L**	DIV (S3)	Missense	NR	(Larsen et al., 2015; Ranza et al., 2020)
**S1596C**	DIV (S3)	Missense	NR	(Fung et al., 2015; Zhang et al., 2015; Boerma et al., 2016)
**I1605R**	DIV (S3-S4)	Missense	NR	(Larsen et al., 2015)
**T1614A**	DIV (S3-S4)	Missense	NR	(Johannesen et al., 2019)
**R1617Q**	DIV (S4)	Missense	Increased persistent current Increased peak current density Shift steady state activation to more negative values Shift steady-state inactivation to more positive values	(Rauch et al., 2012; Ohba et al., 2014; Dyment et al., 2015; Fung et al., 2015; Larsen et al., 2015; Zhang et al., 2015; Fung et al., 2017; Lindy et al., 2018; Johannesen et al., 2019; Schreiber et al., 2020)
**R1620L**	DIV (S4)	Missense	NR	(Rossi et al., 2017)
**L1621W**	DIV (S4)	Missense	NR	(Fung et al., 2015)
**G1625R**	DIV (S4)	Missense	NR	(Deciphering Developmental Disorders Study, 2015)
**L1630P**	DIV (S4)	Missense	NR	(Schreiber et al., 2020)
**I1631N**	DIV (S4)	Missense	NR	(Lindy et al., 2018)
**M1645I**	DIV (S4-S5)	Missense	NR	(Zhang et al., 2015)
**A1650T**	DIV (S4-S5)	Missense	NR	(Ohba et al., 2014; Larsen et al., 2015; Parrini et al., 2017; Gardella et al., 2018; Trivisano et al., 2019)
**A1650V**	DIV (S4-S5)	Missense	NR	(Lindy et al., 2018; Johannesen et al., 2019)
**F1754S**	DIV (S6)	Missense	NR	(Trump et al., 2016)
**V1758A**	DIV (S6)	Missense	Shift steady-state activation to more positive values	(Balciuniene et al., 2019; Johannesen et al., 2019; Zaman et al., 2019)
**N1759T**	DIV (S6)	Missense	NR	(Kim et al., 2019)
**A1763G**	DIV (S6)	Missense	NR	(Denis et al., 2019)
**I1764M**	DIV (S6)	Missense	NR	(Gardella et al., 2018)
**N1768D**	C-Terminus	Missense	Increased spontaneous firing Paroxysmal depolarizing-shift-like complexes, Increased firing frequency Increased persistent current	(Veeramah et al., 2012)
**V1771I**	C-Terminus	Missense	NR	(Johannesen et al., 2019)
**Q1801E**	C-Terminus	Missense	NR	(Larsen et al., 2015)
**R1820X**	C-Terminus	Nonsense	NR	(Møller et al., 2016; Johannesen et al., 2019)
**R1831Q**	C-Terminus	Missense	NR	(Liu et al., 2018)
**R1831W**	C-Terminus	Missense	NR	(Jang et al., 2019)
**T1852I**	C-Terminus	Missense	NR	(Lindy et al., 2018; Heyne et al., 2019)
**L1865P**	C-Terminus	Missense	NR	(Trump et al., 2016)
**R1866Q**	C-Terminus	Missense	NR	(Kothur et al., 2018; Johannesen et al., 2019)
**E1870D**	C-Terminus	Missense	NR	(Boerma et al., 2016)
**R1872L**	C-Terminus	Missense	Enhanced persistent current Increased peak current density Shift steady-state activation to more negative values Shift steady-state inactivation to more positive values	(Wagnon et al., 2016; Sprissler et al., 2017; Lindy et al., 2018; Zaman et al., 2019; Schreiber et al., 2020)
**R1872Q**	C-Terminus	Missense	Enhanced persistent current Increase peak current density Shift steady-state activation to more negative values Shift steady-state inactivation to more positive values	(Larsen et al., 2015; Horvath et al., 2016; Hussain et al., 2016; Arafat et al., 2017; Atanasoska et al., 2018; Lindy et al., 2018)
**R1872W**	C-Terminus	Missense	Enhanced persistent current Increased peak current density Shift steady-state activation to more negative values Shift steady-state inactivation to more positive values	(Ohba et al., 2014; Larsen et al., 2015; Takahashi et al., 2015; Gardella et al., 2018; Denis et al., 2019; Kim et al., 2019; Zaman et al., 2019)
**N1877S**	C-Terminus	Missense	NR	(Anand et al., 2016; Parrini et al., 2017; Wang et al., 2017a; Lindy et al., 2018; Costain et al., 2019; Epifanio et al., 2019; Jain et al., 2019; Ranza et al., 2020)
**P1878S**	C-Terminus	Missense	NR	(Lindy et al., 2018)
Non genetic origin mutations reported*				
**R45Q**	N-terminus	Missense	NR	(Encinas et al., 2019; Heyne et al., 2019)
**A108fsXTer7**	N-terminus	Truncated gene	NR	(Encinas et al., 2019)
**T166I**	DI (S2)	Missense	NR	(Encinas et al., 2019)
**I202N**	DI (S3)	Missense	NR	(Butler et al., 2017a)
**V211L**	DI (S3)	Missense	NR	(Encinas et al., 2019)
**V211A**	DI (S3)	Missense	NR	(Encinas et al., 2019)
**R220H**	D1 (S4)	Missense	NR	(Oates et al., 2018)
**R223S**	DI (S4)	Missense	NR	(Encinas et al., 2019)
**T239A**	DI (S4-S5)	Missense	NR	(Encinas et al., 2019)
**I240V**	DI (S4-S5)	Missense	NR	(Encinas et al., 2019)
**I240L**	DI (S4-S5)	Missense	NR	(Encinas et al., 2019)
**L257V**	DI (S5)	Missense	NR	(Encinas et al., 2019)
**L267V**	DI (S5)	Missense	NR	(Denis et al., 2019)
**I268L**	DI (S5)	Missense	NR	(Encinas et al., 2019)
**F360A**	DI (S5-S6)	Missense	NR	(Encinas et al., 2019)
**M367V**	DI (S5-S6)	Missense	NR	(Encinas et al., 2019)
**R381Q**	DI (S5-S6)	Missense	NR	(Encinas et al., 2019)
**T386R**	DI (S5-S6)	Missense	NR	(Encinas et al., 2019; Schreiber et al., 2020)
**S399P**	DI (S6)	Missense	NR	(Encinas et al., 2019; Heyne et al., 2019)
**V410L**	DI (S6)	Missense	NR	(Encinas et al., 2019)
**Y414F**	DI (S6)-DII (S1)	Missense	NR	(Butler et al., 2017a)
**E416K**	DI (S6)-DII (S1)	Missense	NR	(Encinas et al., 2019)
**Q417P**	DI (S6)-DII (S1)	Missense	NR	(Encinas et al., 2019)
**R530Q**	DI (S6)-DII (S1)	Missense	NR	(Encinas et al., 2019)
**E587Ter**	DI (S6)-DII (S1)	Nonsense	NR	(Encinas et al., 2019)
**R598W**	DI (S6)-DII (S1)	Missense	NR	(Encinas et al., 2019)
**G692R**	DI (S6)-DII (S1)	Missense	NR	(Encinas et al., 2019)
**I763V**	DII (S1)	Missense	NR	(Butler et al., 2017a; Encinas et al., 2019)
**T767I**	DII (S1)	Missense	Shift steady-state activation to more negative values	(Estacion et al., 2014)
**L840P**	DII (S3-S4)	Missense	NR	(Encinas et al., 2019)
**L840F**	DII (S3-S4)	Missense	NR	(Encinas et al., 2019)
**S845F**	DII (S4)	Missense	NR	(Encinas et al., 2019)
**L864V**	DII (S4-S5)	Missense	NR	(Trivisano et al., 2019)
**l868T**	DII (S4-S5)	Missense	NR	(Encinas et al., 2019)
**A874T**	DII (S4-S5)	Missense	NR	(Encinas et al., 2019)
**V881A**	DII (S5)	Missense	NR	(Encinas et al., 2019)
**E936K**	DII (S6)	Missense	NR	(Johannesen et al., 2019)
**L969M**	DII (S6)	Missense	NR	(Encinas et al., 2019)
**S979F**	DII (S6)-DIII (S1)	Missense	NR	(Encinas et al., 2019)
**G1050S**	DII (S6)-DIII (S1)	Missense	NR	(Encinas et al., 2019)
**Y1241C**	DIII (S2)	Missense	NR	(Encinas et al., 2019; Johannesen et al., 2019)
**S1308P**	DIII (S4)	Missense	NR	(Encinas et al., 2019)
**V1315M**	DIII (S4-S5)	Missense	NR	(Encinas et al., 2019)
**L1320F**	DIII (S4-S5)	Missense	NR	(Encinas et al., 2019; Schreiber et al., 2020)
**A1323P**	DIII (S4-S5)	Missense	NR	(Encinas et al., 2019)
**I1327V**	DIII (S4-S5)	Missense	NR	(Oates et al., 2018)
**M1328T**	DIII (S4-S5)	Missense	NR	(Encinas et al., 2019)
**N1329D**	DIII (S4-S5)	Missense	NR	(Butler et al., 2017a)
**G1451S**	DIII (S6)	Missense	NR	(Encinas et al., 2019)
**G1461V**	DIII (S6)	Missense	NR	(Encinas et al., 2019; Schreiber et al., 2020)
**N1466K**	DIII (S6)-DIV (S1)	Missense	NR	(Encinas et al., 2019)
**F1467C**	DIII (S6)-DIV (S1)	Missense	NR	(Encinas et al., 2019)
**Q1470H**	DIII (S6)-DIV (S1)	Missense	NR	(Trivisano et al., 2019)
**I1479V**	DIII (S6)-DIV (S1)	Missense	NR	(Encinas et al., 2019)
**A1491V**	DIII (S6)-DIV (S1)	Missense	Shift steady-state activation to more negative values	(Johannesen et al., 2018; Trivisano et al., 2019)
**M1492V**	DIII (S6)-DIV (S1)	Missense	NR	(Encinas et al., 2019; Ranza et al., 2020)
**Q1501K**	DIII (S6)-DIV (S1)	Missense	NR	(Encinas et al., 2019)
**Splice donor** **c.4419+1A>G**	DIII (S6)-DIV (S1)	Truncated gene	NR	(Encinas et al., 2019)
**M1536I**	DIV (S1)	Missense	NR	(Encinas et al., 2019)
**V1592L**	DIV (S3)	Missense	NR	(Encinas et al., 2019)
**I1594L**	DIV (S3)	Missense	NR	(Encinas et al., 2019)
**S1596C**	DIV (S3)	Missense	NR	(Encinas et al., 2019)
**T1614A**	DIV (S3-S4)	Missense	NR	(Encinas et al., 2019)
**R1617Q**	DIV (S4)	Missense	Enhanced persistent current Increased peak current density Shift steady-state activation to more negative values Shift steady-state inactivation to more positive values	(Encinas et al., 2019)
**R1617P**	DIV (S4)	Missense	NR	(Encinas et al., 2019)
**G1625R**	DIV (S4)	Missense	NR	(Encinas et al., 2019)
**L1630P**	DIV (S4)	Missense	NR	(Encinas et al., 2019)
**F1642C**	DIV (S4-S5)	Missense	NR	(Encinas et al., 2019)
**A1650T**	DIV (S4-S5)	Missense	NR	(Trivisano et al., 2019)
**A1650V**	DIV (S4-S5)	Missense	NR	(Encinas et al., 2019)
**I1654N**	DIV (S4-S5)	Missense	NR	(Johannesen et al., 2019)
**N1759S**	DIV (S6)	Missense	NR	(Encinas et al., 2019; Schreiber et al., 2020)
**M1760I**	DIV (S6)	Missense	Shift steady-state activation to more negative values Increase action potential firing frequency	(Liu et al., 2019)
**N1768D**	C-Terminus	Missense	Increased spontaneous firingParoxysmal depolarizing shift like complexes Increased firing frequency Enhanced persistent current	(Veeramah et al., 2012; Encinas et al., 2019)
**K1807N**	C-Terminus	Missense	NR	(Encinas et al., 2019)
**R1831W**	C-Terminus	Missense	NR	(Encinas et al., 2019)
**D1833H**	C-Terminus	Missense	NR	(Johannesen et al., 2019)
**T1852I**	C-Terminus	Missense	NR	(Encinas et al., 2019; Ranza et al., 2020)
**R1872L**	C-Terminus	Missense	Increased persistent current Increased peak current density Shift steady state activation to more negative values Shift steady inactivation to more positive values	(Encinas et al., 2019)
**N1877S**	C-Terminus	Missense	NR	(Johannesen et al., 2019; Schreiber et al., 2020)
**R1904C**	C-Terminus	Missense	NR	(Encinas et al., 2019)
				

*Non genetic origin mutations reported: Mutations described through clinical diagnosis, but the mutation type (Mendelian or de novo) were not reported, mainly due to the lack of parents to perform genotyping and difficulty in contacting the family. Not Reported (NR); Domain (D); Segment (S).

NaV1.6 is expressed since prenatal, during fetal development (Plummer et al., 1997). Shortly after birth, expression begins to increase, reaching maximum levels during the first years of life. This channel is widely expressed in the nodes of Ranvier of myelinated axons and in the distal part of the axon initial segments (AIS), although they are also ubiquitously present throughout the central and peripheral nervous systems, in both excitatory and inhibitory neurons (Caldwell et al., 2000; Oliva et al., 2012). For these reasons, NaV1.6 is one of the most common subtype of voltage-gated sodium channels found in the central nervous system (Caldwell et al., 2000). In humans, the distal AIS is the specialized membrane region in neurons where action potentials are triggered. Overexpression of Nav1.6 in the AIS has been shown to cause an increase in spontaneous and repetitive firing (Hu et al., 2009; Sun et al., 2013), a possible explanation for why *SCN8A* mutations in epilepsy patients are predominantly GoF and affect the action potential threshold. On the other hand, the functional importance of Nav1.6 in inhibitory interneurons is not clear yet, but evidence indicates a role for Nav1.6 in establishing synaptic inhibition in the thalamic network (Makinson et al., 2017), supporting the LoF results caused by missense mutations in the mature protein. These attributes lead to different network effects in distinct nervous system circuits. Mutations in *SCN8A* are associated with early-infantile epileptic encephalopathy type 13 (EIEE13; OMIM #614558), a phenotypically heterogeneous early onset epilepsy, with seizure onset happening before 18 months of age (Hammer et al., 2016). Patients typically develop intellectual disability, developmental delay, and movement disorders (Ohba et al., 2014; Gardella et al., 2016; Johannesen et al., 2018). Co-occurrence of autism spectrum disorders, severe juvenile osteoporosis, bradyarrhythmia, cortical visual impairment, and gastrointestinal disorders have been reported in rare cases (Larsen et al., 2015; Hammer et al., 2016; Rolvien et al., 2017; Gardella et al., 2018). Sudden unexpected death in epilepsy (SUDEP) has also been linked to *SCN8A* mutations, described as the most common cause of death in epilepsy patients. Reports have suggested that patients with *SCN8A*-related epilepsy have increased risk of SUDEP, ranging from 1% to 10% (Hammer et al., 2016; Wang et al., 2017a; Gardella et al., 2018; Johannesen et al., 2018). One possible correlation of SUDEP with *SCN8A*-related epilepsy is the presence of NaV1.6 in heart muscles and tissues, being broadly expressed within ventricular myocytes (Maier et al., 2002). Single mutations may affect heart function, causing failure of the cardiorespiratory system and, consequently, death (Haufe et al., 2005; Noujaim et al., 2012). Most recently, few cases of *SCN8A*-related epilepsies with “milder” phenotype were associated with benign familial infantile seizures-5 (BFIS5; OMIM #617080) (Anand et al., 2016; Gardella et al., 2016; Han et al., 2017).

An increase in new described variants made some mutation patterns visible. Wagnon and co-workers observed numerous cases of the same epiletogenic mutation, and suggested that CpG dinucleotides are mutation hotspots that, through enzymatic processing and epigenetic methylation, can convert cytosine to thymine, such as arginine residues 1617 and 1872 (Wagnon and Meisler, 2015). The prominent number of new variant cases in Arg850 indicates this residue as a new hotspot, since the arginine codon holds a CpG dinucleotide. In addition to these mutation hotspots, residues I763, I1327, G1475, A1650, and N1877 do not present CpG dinucleotides in their codon; however, they can be considered recurrent mutations in view of its high repetition cases in literature (**Table 4**).

The mutation at position c.- 8A>G produces a pathogenic variant, despite not being inside the gene, or promoter regions, transcriptional and translational sites. This mutation was detected in an untranslated region outside of the Kozak consensus sequence (Johannesen et al., 2019). Its role in *SCN8A*-related epilepsy is still unclear; however, it may change RNA stability, modulate transcriptional factors and promoters, modify the initiation of translation, or work as an enhancer or silencer in the splicing pattern. For all the reasons mentioned above, Nav1.6 variants are predominantly harmful, and the same mutation can lead to different phenotypes, hampering the correlation of genotypes with phenotypes (Blanchard et al., 2015).

*SCN8A* mutations can be both GoF and LoF, which will likely require different approaches and targets. Even in patients with the same SCN8A mutation, the response to the same drug treatment can differ. Surprisingly, most *SCN8A*-related epilepsies respond favorably to channel blockers. Phenytoin and lacosamide are SBCs widely used in SCN8A mutations with GoF effect, while carbamazepine exhibited positive seizure control in a patient with NaV 1.6 mutation and LoF effect. (Blanchard et al., 2015; Wagnon and Meisler, 2015; Hammer et al., 2016; Perucca and Perucca, 2019). Phenytoin demonstrated effectiveness in decreasing seizure episodes in several patients with *SCN8A*-related epilepsies, however, side effects during prolonged use are very common (Boerma et al., 2016; Braakman et al., 2017). A recent study of a DS model using zebrafish demonstrated the use of the channel blocking compound MV1312, which is 5–6 fold selectivity of NaV1.6 over NaV1.1–1.7, reduced burst movement phenotype and the number of epileptiform events, activity similar to that described with the use of a selective NaV1.1 activator AA43279 (Weuring et al., 2020). Selective Nav1.6 blockers may represent a new therapeutic strategy for DS patients. In addition, two precise and promising drugs have been described recently: XEN901 and GS967. XEN901 is an arylsulfonamide highly selective and potent NaV1.6 inhibitor that binds specifically in voltage sensor domain IV, avoiding recovery from inactivation. GS967 is a NaV1.6 modulator that inhibits the persistent sodium current and exhibits a protective effect (Baker et al., 2018; Bialer et al., 2018).

### NaV1.7 

The *SCN9A* gene encodes for the NaV1.7 channel, located in chromosome 2q24 (Yang et al., 2018). NaV1.7 is expressed preferably in the PNS, but it is also expressed in the CNS (Cen et al., 2017). Consequently, mutations in this channel are generally related to pain disorders (Young, 2007; Han et al., 2009; Doty, 2010; Rush et al., 2018); however, current studies have described a correlation between epilepsy and this channel (OMIM #603415).

Pain disorder mutations with GoF are related with diseases such as erythromelalgia (EMI), small-fiber neuropathy (SFN) and paroxysmal extreme pain disorder (PEPD), and mutations with LoF are related with congenital insensitivity to pain (CIP) (Cen et al., 2017). Epilepsy studies such as Zhang S. et al. (2020) showed mutations with GoF phenotype: W1150R, N641Y, and K655R mutations (**Table 5**). Being that, after treatment with OXC (120 µmol/L), N641Y and K655R reduced sodium current and decreased the opening time of the channel, while W1150R did not alter that (Zhang S. et al., 2020). However, in a study conducted by Yang et al. (2018), one of the patients presented generalized tonic-clonic seizure with fever, treated with sodium valproic acid, and a LoF mutation I1901fs was observed (Yang et al., 2018) (**Table 5**).

**Table 5:** SCN9A-related epilepsies identified in clinical patients through WES and/or NGS.

Variant	Location	Mutation	Disease	Alteration on *biophysical properties* or/and Clinical report	Reference
**Inherited mutation**					
**Q10R**	N-terminal	Missense	GEFS+	Febrile and afebrile seizures Generalized tonic-clonic seizures	(Cen et al., 2017)
**G327E**	DI	Missense	Epilepsy	Generalized tonic-clonic seizure	(Yang et al., 2018)
**N641Y**	DI- DII	Missense	FS	Reduced electroconvulsive seizure thresholds (Knocking mice) Increased corneal kindling acquisition rates (Knocking mice) Increased current density Faster recovery from inactivation More susceptible to clonic and tonic seizures induced by electrical stimulation (mice) Enhanced persistent current	(Singh et al., 2009; Zhang S. et al., 2020)
**I1901fs**	C-terminal	Frameshift	Epilepsy	Generalized tonic-clonic seizure	(Yang et al., 2018)
Non genetic origin mutations reported*					
**K655R**	DI-DII	Missense	FS	Enhanced persistent current Faster recovery from inactivation	(Zhang S. et al., 2020)
**W1150R**	DII-DIII	Missense	FS	Increased current density Enhanced persistent current Focal seizures with secondary generalization High-potential spike activity, paroxysmal release, and d frequency power enhancement (EEG)	(Zhang S. et al., 2020)

Variants of NaV1.7 have been related with febrile seizure or GEFS+ (Cen et al., 2017; Zhang S. et al., 2020) and even as asymptomatic (Singh et al., 2009). However, SCN9A can act as a putative modifier of NaV1.1 gene; consequently, it can elevate the severity of patients’ phenotype (Guerrini et al., 2010; Parihar and Ganesh, 2013). Some NaV1.7 mutations could probably contribute to generate a genetic susceptibility to a known epilepsy disease called Dravet syndrome, in a multifactorial way, as a modifier gene (Singh et al., 2009; Doty, 2010; Mulley et al., 2013; Cen et al., 2017; Zhang T. et al., 2020). That said, some rare cases of DS found in patients can be understood (Mulley et al., 2013). For example, even parents with mild phenotype had children with severe cases (Guerrini et al., 2010).

## Conclusion and Future Perspectives

The past two decades have enabled remarkable progress in understanding monogenic epilepsies. NaV-related epilepsies are diseases of phenotypic heterogeneity, since sodium channels are found in both the CNS and the PNS, but with different expression ranges. The lack of a clear genotype-phenotype correlation to help guide patient counseling and management by healthcare professionals makes it very complex, and often expensive, to determine a correct diagnosis. Consequently, identify the monogenic mutation in individual patients with epilepsy is important not only for diagnosis and prognosis, but also for a correct treatment approach (Mei et al., 2017; Reif et al., 2017).

Susceptibility to specific treatments may be different depending on the disease’s features, diverging even in patients who share the same phenotype and/or mutation (Weber et al., 2014). The use of innovative tools that facilitate and prevent diagnostic delay in patients with epilepsy of unknown etiology onset is crucial. WES has proved to be a valuable tool to circumvent the lack of an accurate and fast diagnosis to epilepsies caused by monogenic mutation, and also cheapen and drastically anticipate diagnosis. This genetic diagnostic tool may reduce traditional investigation costs by 55 to 70%, besides avoiding further pre-surgical evaluation and epilepsy surgery (Kothur et al., 2018; Oates et al., 2018). In addition to the financial impact, it can anticipate diagnosis from nearly 3.5 years to 21 days, optimizing management and health care support (Oates et al., 2018).

Effective and safe drugs for the treatment of monogenic epilepsy are still an unmet clinical need. The drugs currently available in the pharmaceutical market are only palliative methods for a temporary control of the disease symptoms, and few patients will benefit from the existing pharmacotherapy, since a great number of patients treated with antiepileptic channel blockers showed no improvement in clinical conditions. Also, most treated patients exhibited manifold side effects, and the prolonged use of these medications proved to be harmful (Boerma et al., 2016; Braakman et al., 2017). Several examples of novel and promising candidate compounds to be used in personalized medicine, such as precision therapies, have been suggested. A previously study demonstrated that CBD at 1μM inhibit preferably resurgent currents than transient current in Nav1.6 WT and also inhibit peak resurgent current in Nav1.6 mutant N1768D, with less effect in current density and without alters voltage dependence of activation (Patel et al., 2016) Possibly the modulation of CBD over mutations in SCN8A that promotes a phenotype with increased resurgent currents would cause a reduction in the causative excitability of epileptic seizures. CBD also showed its ability to preferential inhibit resurgent currents in the NaV1.2 channel (Mason and Cummins, 2020). Due the role of Nav1.2 and Nav1.6 in excitatory neurons, preferentially inhibition in resurgent currents by CBD could possibly reduce the excitability in that subset of neurons and decrease the frequency of seizures by a change in threshold of activation and repetitive fire (Lewis and Raman, 2014). Peptides derived from scorpion and spider venom are well known modulator tools in neuroscience and showed specific capacity to regulate most NaV subtypes related with monogenic epilepsy, unlike the available promiscuous drugs that generally interact with any NaV channel isoform (Schiavon et al., 2006; Israel et al., 2018; Richards et al., 2018; Tibery et al., 2019; Zhang et al., 2019). Bioengineering tools, like antisense oligonucleotides capable to regulate NaV1.1 channels expression, and the peptide Hm1, that modulates the function of this subtype of sodium channel, are some innovative treatment examples (Richards et al., 2018; Stoke Therapeutics, 2018).

However, there is still a long path toward the development of efficacious treatments for NaV-related epilepsies. Recent studies offered a better understanding of the complexity of the phenotypic and genetic spectrum, which has only just begun to be elucidated. Biomolecular diagnostic tools will drastically reduce the developmental and cognitive effects caused by misdiagnosis and late diagnosis, and maybe, in the upcoming years, the treatment for inherited NaV-related epilepsies will be conducted ideally *in utero*, during the prenatal stage. Moreover, further functional studies, with greater cohorts of patients, represent an urgent medical need for a better understanding of the correlations between genotype and clinical symptoms, as well as the different NaV-related epilepsies mechanisms. These studies will improve clinical efficacy and promote safety diagnostic strategies, as well as develop prognosis prediction in the near future.

## Author Contributions

All authors made an intellectual and direct contribution for this article and approved it for publication.

## Funding

This study was supported by the Conselho Nacional de Desenvolvimento Científico e Tecnológico (CNPq) [407625/2013-5] and the Fundação de Apoio à Pesquisa do Distrito Federal (FAPDF) [grants 193.001.202/2016 and 00193.0000109/2019-17].

## Conflict of Interest

The authors declare that the research was conducted in the absence of any commercial or financial relationships that could be construed as a potential conflict of interest.
