# Supplementary material for: Epilepsy-Related Voltage-Gated Sodium Channelopathies: A Review
Source: Front Pharmacol. 2020 Aug 18;11:1276. doi: 10.3389/fphar.2020.01276 (PMC7461817; doi:10.3389/fphar.2020.01276)
Supplement: Supplementary file 1 [file DataSheet_1.pdf]

Supplementary Material

Table S1. Complete epilepsy-related mutations identified in NaV1.1 table

Variant	Location	Mutation	Disease	Alteration in biophysical properties and/or Clinical report	Reference
M1	N-terminal	Start codon mutation	SME	NR	(Depienne et al., 2008)
M1T	N-terminal	Start codon mutation	DS	NR	(Zuberi et al., 2011)
Q3X (De novo)	N-terminal	Nonsense	DS	First seizures<br>Generalized tonic clonic seizures	(Claes et al., 2003; Lim et al., 2011)
L6P (Paternal)	N-termial	Nonsense	GEFS+	NR	(Cetica et al., 2017)
P8fsX12 (De novo)	N-terminal	FrameShift	DS	NR	(Wang et al., 2012)
P11Tfs*18	N-terminal	FrameShift	GEFS+	NR	(Cetica et al., 2017)
P11QfsX80 (De novo)	N-terminal	FrameShift	EE	NR	(Wei et al., 2018)

P11LfsX81	N-terminal	FrameShift	DS	NR	(Ishii et al., 2017)
P14SfsX78 (De novo)	N-terminal	FrameShift	DS	NR	(Harkin et al., 2007)
P17del	N-terminal	In-Frame deletion	DS	NR	(Zuberi et al., 2011)
T18del	N-terminal	In-Frame deletion	DS	NR	(Djémié et al., 2016)
A19X	N-terminal	Nonsense	SME	NR	(Lee et al., 2014)
E20X (De novo)	N-terminal	Nonsense	SME<br>DS	NR	(Ceulemans et al., 2004; Depienne et al., 2008; Wang et al., 2012)
A24GfsX4	N-terminal	FrameShift	DS	NR	(Zuberi et al., 2011)
A24T	N-terminal	Missense	SME	NR	(Xu et al., 2014)
E26X	N-terminal	Nonsense	DS	NR	(Zuberi et al., 2011)

A27T (Paternal)	N-terminal	Missense	GEFS+ SMEB	Diffuse spikes, prevailing in posterior regions (EEG)	(Nicita et al., 2010)
R28C (Paternal)	N-terminal	Missense	GEFS+	NR	(Lossin, 2009; Lal et al., 2016);
E32X	N-terminal	Nonsense	EP	NR	(Lindy et al., 2018)
L33A	N-terminal	Missense	GEFS+	NR	(Cetica et al., 2017)
L38fsX54 (De novo)	N-terminal	FrameShift	SME DS	NR	(Wallace et al., 2003; Depienne et al., 2008)
L41X	N-terminal	Nonsense	SME	NR	(Xu et al., 2014)
D43 MetfsX47	N-terminal	FrameShift	SME DS	Acute encephalopathy resulting in severe neurologic sequelae Generalized slow waves (EEG)	(Takayanagi et al., 2010; Wang et al., 2012)
D43MetfsX49 (De novo)	N-terminal	FrameShift	DS	NR	(Depienne et al., 2008)
D45N (Paternal)	N -terminal	Missense	Epilepsy-Aphasia FS+	NR	(Zuberi et al., 2011; Carvill et al., 2013)

D45E	N-terminal	Missense	DS	Multifocal discharges (EEG)<br><br>Hippocampal atrophy (MRI)	(Zhou et al., 2018)
N47MfsX45	N-terminal	FrameShift	Epilepsy	NR	(Stosser et al., 2018)
N47LfsX45	N-termianl	FrameShift	Epilepsy	NR	(Lindy et al., 2018)
G58X (De novo)	N-terminal	Nonsense	DS<br>Focal Epilepsy	Moderate mental retardation<br>Autistic caracteristics<br>Hyperactivity<br>Periventricular nodular heterotopia (MRI)	(Barba et al., 2014)
G58R	N-terminal	Missense	FS+	Febrile and afebrile GTC<br>Large amplitude and diffuse (EEG)	(Mina et al., 2015)
G58V	N-terminal	Missense	DS	NR	(Depienne et al., 2008)
L61F	N-terminal	Missense	DS	NR	(Depienne et al., 2008)
L61P (Paternal)	N-terminal	Missense	DS	Febrile seizures	(Halvorsen et al., 2016)

P62fsX30	N-terminal	FrameShift	SME	NR	(Xu et al., 2014)
F63L(Paternal)	N-terminal	Missense	DS	Severe developmental delay<br><br>Spike and Waves in right fronto-temporal region with spreading (EEG)	(Nicita et al., 2010)
Y65X (De novo)	N-terminal	Nonsense	DS	Generalized tonic-clonic seizures	(Zucca et al., 2008)
I68T (Maternal)	N-terminal	Missense	SMEB	NR	(Zuberi et al., 2011)
Ser74Pro (De novo)	N-terminal	Missense	FS+	NR	(Marini et al., 2007)
E75D (De novo)	N-terminal	Missense	DS	Slow-spike-wave complexes (EEG)	(Arafat et al., 2017)
P76T (De novo)	N-terminal	Missense	DS	NR	(Ishii et al., 2017)
L77P (De novo)	N-terminal	Missense	DS	NR	(Kothur et al., 2018)
E78X	N-terminal	Nonsense	DS	NR	(Surovy et al., 2016)

E78GfsX7	N-terminal	FrameShift	Epilepsy	NR	(Lindy et al., 2018)
E78D (De novo)	N-terminal	Missense	DS	NR	(Nabbout et al., 2003)
D79N(De novo)	N-terminal	Missense	DS	NR	(Depienne et al., 2008; Zuberi et al., 2011)
D79H (De novo)	N-terminal	Missense	SMEB	NR	(Harkin et al., 2007; Depienne et al., 2008)
L80_D81del (De novo)	N-terminal	In-frame deletion	DS	Pharmacoresistant	(Usluer et al., 2016)
D81N (De novo)	N-terminal	Missense	DS	Severe Motor and mental delay<br>Severe Autistic<br>Multi-focal spike-waves (EEG)	(Usluer et al., 2016)
D81E (De novo)	N-terminal	Missense	Epilepsy	NR	(Lindy et al., 2018)
Y83X (De novo)	N-terminal	Nonsense	SMEB	NR	(Zuberi et al., 2011)
Y84fsX4	N-terminal	FrameShift	DS	NR	(Ishii et al., 2017)

Y84C (De novo)	N-terminal	Missense	DS	NR	(Harkin et al., 2007; Wang et al., 2012)
N86SfsX7	N-terminal	FrameShift	DS	NR	(Ishii et al., 2017)
F90S(Paternal)	N-terminal	Missense	DS	Multifocal spikes, frontal-dominant spike-waves complex (EEG)	(Sun et al., 2008a; Wang et al., 2012; Xu et al., 2014; Butler et al., 2017b)
I91T (De novo)	N-terminal	Missense	DS	Frontal-dominant spike-waves complex (EEG)	(Sun et al., 2008a; Xu et al., 2014)
I91SfsX4	N-terminal	FrameShift	SMEB	NR	(Depienne et al., 2008; Villeneuve et al., 2014)
I91fs	N-terminal	FrameShift	SME	NR	(Lee et al., 2014)
L93EfsX2 (De novo)	N-terminal	FrameShift	DS	NR	(Riva et al., 2009)
L93S	N-terminal	Missense	SME	NR	(Lee et al., 2014)
L93X	N-terminal	Nonsense	DS	NR	(Cetica et al., 2017)
N94L (De novo)	N-terminal	Missense	DS	NR	(Ishii et al., 2017)

G96EfsX24 (De novo)	N-terminal	FrameShift	NR	Genetic generalized epilepsy with intellecutal disability	(Fry et al., 2016)
L97fsx14	N-terminal	FrameShift	SME	NR	(Xu et al., 2014)
A98P (De novo)	N-terminal	Missense	DS	NR	(Zuberi et al., 2011)
A98PfsX14	N-terminal	FrameShift	Epilepsy	NR	(Lindy et al., 2018)
R101W (De novo; Paternal)	N-terminal	Missense	DS<br>SMEB	NR	(Harkin et al., 2007; Depienne et al., 2008; Sun et al., 2010; Wang et al., 2012; Fry et al., 2016; Kothur et al., 2018)
R101Q(De novo; Maternal)	N-terminal	Missense	DS<br>SMEB<br>GEFS+<br>PEFS+	Psychomotor retardation	(Fukuma et al., 2004; Harkin et al., 2007; Marini et al., 2007; Depienne et al., 2008; Sun et al., 2010; Zuberi et al., 2011; Wang et al., 2012; Tonekaboni et al., 2013; Lee et al., 2014; Djémié et al., 2016)
S103G (Paternal)	N-terminal	Missense	SME<br>DS	Severe mental decline<br>Rare-spike wave complex (EEG)	(Fujiwara, 2003; Ebrahimi et al., 2010; Tonekaboni et al., 2013)
S103I	N-terminal	Missense	DS	NR	(Xu et al., 2015)

A104V (De novo)	N-terminal	Missense	DS	Epileptic discharges, slow spike and weave; sharp wave, sharp and slow wave complex (EEG)	(Kwong et al., 2012; Myers et al., 2017a)
T105I	N-terminal	Missense	DS	NR	(Wang et al., 2012; Lindy et al., 2018)
S106F (Paternal)	N-terminal	Missense	Focal epilepsy	Right temporal pariental occipital slow-wave and generalized spike-wave complex (EEG)	(Barba et al., 2014)
A107D (De novo)	N-terminal	Missense	DS	NR	(Ishii et al., 2017)
L108R	N-terminal	Missense	DS<br>SMEB	Generallized tonic-clonic seizures	(Tang et al., 2011; Zuberi et al., 2011)
L108P	N-terminal	Missense	DS	NR	(Xu et al., 2015)
T112I	N-terminal	Missense	SME	Rare spike-wave complex and rare multifocal spikes (EEG)	(Fujiwara, 2003)
P113T	N-terminal	Missense	SMEB	NR	(Xu et al., 2014)
P113L	N-terminal	Missense	Epilepsy	NR	(Lindy et al., 2018)

F114LfsX6 (De novo)	N-terminal	FrameShift	SME	NR	(Kodera et al., 2013)
L117QfsX6 (De novo)	N-terminal	FrameShift	DS	NR	(Depienne et al., 2008)
R118S (De novo)	N-terminal	Missense	DS	Generalized tonic-clonic seizures<br>Severe mental retardation	(Zucca et al., 2008)
L123RfsX11	DIS1	FrameShift	Epilepsy	NR	(Lindy et al., 2018)
I124N(Maternal)	DIS1	Missense	SME	NR	(Depienne et al., 2008)
H127N (De novo)	DIS1	Missense	SMEB<br>DS	NR	(Zuberi et al., 2011; Cetica et al., 2017)
H127Y (De novo)	DIS1	Missense	GEFS+	NR	(Djémié et al., 2016)
H127L	DIS1	Missense	DS	NR	(Xu et al., 2015)
S128X (De novo)	DIS1	Nonsense	DS<br>FS+<br>SME	NR	(Nabbout et al., 2003; Zuberi et al., 2011; Carvill et al., 2014)

L129del (De novo)	DIS1	In-frame deletion	DS	NR	(Riva et al., 2009)
S131R	DIS1	Missense	NR	NR	(Fry et al., 2016)
C136WfsX 14	DIS1	FrameShift	DS	NR	(Cho et al., 2018)
I138FfsX11 (Paternal	DIS1	FrameShift	DS	NR	(Zuberi et al., 2011)
T140R (De novo)	DIS1	Missense	DS	NR	(Ishii et al., 2017)
Val143fsX1 6 (De novo)	DIS1	FrameShift	DS	NR	(Wang et al., 2012)
Phe144Tyrf sX5 (De novo)	DIS1	Frameshift	SME<br>DS	Moderate psychomotor retardation	(Fukuma et al., 2004; Zuberi et al., 2011; Wang et al., 2012; Villeneuve et al., 2014)
Met145Asp fsX4 (De novo)	DIS1	Frameshift	PEFS+	Generalized tonic-clonic seizures without any provoked factors	(Yu et al., 2010)
Met145Val	DIS1	Missense	DS	NR	(Xu et al., 2015)
M145T (Paternal)	DIS1	Missense	Unidentified epilepsy	Decrease current density	(Mantegazza et al., 2005; Colosimo et al., 2007)

				Shift of steady-state inactivation to more positive values	
M145L	DIS1	Missense	DS	NR	(Lindy et al., 2018)
W153X	D1S1-S2	Nonsense	DS<br>SMEB	NR	(Zuberi et al., 2011)
E158Q	DIS1-S2	Missense	Epilepsy	NR	(Lindy et al., 2018)
Y159SfsX13	DIS2	FrameShift	Epilepsy	NR	(Lindy et al., 2018)
T162P (De novo)	DIS2	Missense	DS	NR	(Riva et al., 2009)
T162I	DIS2	Missense	SME	NR	(Cetica et al., 2017)
G163E	DIS2	Missense	SME	NR	(Hattori et al., 2008)
Y165X (De novo)	DIS2	Nonsense	DS	NR	(Nabbout et al., 2003)

T166VfsX5 (De novo)	DIS2	FrameShift	DS	NR	(Harkin et al., 2007)
S169IfsX10 8	DIS2	FrameShift	SME	Severe mental retardation<br>Autistic features	(Le Gal et al., 2010; Barba et al., 2014)
S169P (Mosaic)	DIS2	Missense	DS	NR	(Yang et al., 2017)
I171L(De novo)	DIS2	Missense	SMEB	NR	(Harkin et al., 2007)
I171R (De novo)	DIS2	Missense	DS	NR	(Depienne et al., 2008)
A175T (De novo)	DIS2	Missense	SMEB	NR	(Harkin et al., 2007)
A175V (De novo)	DIS2	Missense	DS	NR	(Depienne et al., 2008)
G177R (De novo)	DIS2-S3	Missense	SME	NR	(Hattori et al., 2008; Reyes et al., 2011)
G177DfsX4	DIS2-S3	FrameShift	DS	Generalized tonic-clonic seizures	(Fujiwara, 2003)
G177E (De novo)	DIS2-S3	Missense	SME<br>DS	Non-functional channel	(Nabbout et al., 2003; Ohmori et al., 2006; Usluer et al., 2016)

G177Val	DIS2-S3	Missense	DS	NR	(Cetica et al., 2017)
P178Ser	DIS2-S3	Missense	DS	NR	(Wang et al., 2012)
C179Arg	DIS2-S3	Missense	DS	NR	(Wang et al., 2012)
L180X (De novo)	DIS2-S3	Nonsense	DS	Focal spike wave (EEG)	(Liu et al., 2018)
E181X (Famial)	DIS2-S3	Nonsense	GEFS+	NR	(Orrico et al., 2009)
D182IfsX34 (De novo)	DIS2-S3	FrameShift	DS	NR	(Yang et al., 2017)
T184YfsX93	DIS2-S3	FrameShift	SMEB	NR	(Lindy et al., 2018)
D188V (Paternal)	DIS2-S3	Missense	GEFS+	Resistance to entry into closed state inactivation during high frequency stimulation	(Wallace et al., 2001; Cossette et al., 2003; Rilstone et al., 2012)
W190R(Maternal)	DIS3	Missense	DS	NR	(Fukuma et al., 2004; Wang et al., 2012)

W190X (De novo)	DIS3	Nonsense	DS	Febrile, partial and generalized tonic-clonic and myo-clonic seizures<br>Severe intellectual disability.	(Marini et al., 2007; Kwong et al., 2012)
N191Y	DIS3	Missense	DS	NR	(Depienne et al., 2008)
N191T	DIS3	Missense	DS	NR	(Ishii et al., 2017)
N191L	DIS3	Missense	DS	NR	(Depienne et al., 2008)
W192X (De novo)	DIS3	Nonsense	DS	NR	(Depienne et al., 2008)
L193F(Paternal)	DIS3	Missense	GEFS+	Generalized tonic–clonic seizures	(Cui et al., 2011)
D194N(De novo)	DIS3	Missense	SME<br>DS<br>SMEB	NR	(Harkin et al., 2007; Riva et al., 2009; Azmanov et al., 2010; Zuberi et al., 2011; Kodera et al., 2013; Cho et al., 2018; Lindy et al., 2018)
D194H	DIS3	Missense	DS	NR	(Zuberi et al., 2011)
D194G	DIS3	Missense	DS	NR	(Depienne et al., 2008)

D194A	DIS3	Missense	SME	EEG reveals backgrond slow	(Lee et al., 2014)
T199R (De novo)	DIS3	Missense	SMEB	NR	(Harkin et al., 2007)
T199SfsX15	DIS3	Frameshift	DS	NR	(Cho et al., 2018)
A201E	DIS3	Missense	DS	NR	(Do et al., 2017; Lindy et al., 2018)
Y202X (De novo)	DIS3	Nonsense	DS	NR	(Wang et al., 2012)
V207G	DIS3	Missense	EE	Early-onset multifocal seizures	(Daoud et al., 2016)
L209PfsX68	DIS3-S4	Frameshift	DS	NR	(Møller et al., 2016)
G210D	DIS3-S4	Missense	DS	NR	(Ishii et al., 2017)
V212A	DIS3-S4	Missense	SME	NR	(Hattori et al., 2008)

S213HfsX9	DIS3-S4	Frameshift	Epilepsy	NR	(Lindy et al., 2018)
S213W (De novo)	DIS3-S4	Missense	Epilepsy	Febrile and afebrile seizures<br>Developmental delay	(Butler et al., 2017a)
L215_R219 delinsfsX	DIS3-S4	Frameshift	DS	NR	(Ko et al., 2018)
T217L (De novo)	DIS4	Missense	DS	NR	(Riva et al., 2009)
F218L (Paternal, Maternal)+	DIS4	Missense	GEFS+<br>PEFS+	Slow wave discharges in left posterior region with occasional generalization; A second EEG reveal multifocal spike and generalized slow wave-spike complex (EEG)	(Livingston et al., 2009; Catarino et al., 2011)
R219G (De novo)	DIS4	Missense	DS	NR	(Usluer et al., 2016)
R219T	DIS4	Missense	Epilepsy	NR	(Lindy et al., 2018)
R219SfsX57 (De novo)	DIS4	FrameShift	DS	Generalized toic-clonic seizures	(Claes et al., 2001)
V220fsX4	DIS4	FrameShift	SME	NR	(Xu et al., 2014)

V220D	DIS4	Missense	Malignat migrating partial seizures of infancy	NR	(Zhang et al., 2015)
L221P	DIS4	Missense	Epilepsy	NR	(Lindy et al., 2018)
R222X (De novo)	DIS4	Nonsense	DS<br>SMEB	No measurable current	(Claes et al., 2001; Nabbout et al., 2003; Fukuma et al., 2004; Harkin et al., 2007; Depienne et al., 2008; Orrico et al., 2009; Zuberi et al., 2011; Wang et al., 2012; Xu et al., 2014; Esterhuizen et al., 2018)
A223E (De novo)	DIS4	Missense	DS	NR	(Depienne et al., 2008)
L224S	DIS4	Missense	Epilepsy	NR	(Lindy et al., 2018)
K225N	DIS4	Missense	SME	NR	(Lee et al., 2014; Xu et al., 2014; Lindy et al., 2018)
T226M (De novo)	DIS4	Missense	DS	NR	(Harkin et al., 2007; Mulley et al., 2013; Dhamija et al., 2014; Muona et al., 2015; Lindy et al., 2018)
T226K	DIS4	Missense	SME	NR	(Catarino et al., 2011)

T226R (De novo)	DIS4	Missense	DS	NR	(Wang et al., 2012; Lindy et al., 2018)
I227T (De novo)	DIS4	Missense	DS	NR	(Zuberi et al., 2011)
I227S (De novo)	DIS4	Missense	SME<br>SMEB	Low current density (no detectable)	(Nabbout et al., 2003; Ohmori et al., 2006; Depienne et al., 2008; Riva et al., 2009; Mak et al., 2011; Wang et al., 2012; Lindy et al., 2018)
S228P	DIS4	Missense	DS		(Parrini et al., 2017)
G232S	DIS4	Missense	DS	NR	(Depienne et al., 2008; Zuberi et al., 2011)
L233EfsX43 (De novo)	DIS4	FrameShift	DS	NR	(Petrelli et al., 2012)
L233G	DIS4	Missense	Epilepsy	NR	(Lindy et al., 2018)
L233R (De novo)	DIS4	Missense	DS	NR	(Zuberi et al., 2011)
T235S	DIS4-S5	Missense	GEFS+	NR	(Cetica et al., 2017)

Il236LfsX5	DIS4-S5	FrameShift	Epilepsy	NR	(Lindy et al., 2018)
A239T (De novo, Parternal)	DIS4-S5	Missense	DS	NR	(Harkin et al., 2007; Sun et al., 2008a; Cetica et al., 2017)
A239V (De novo)	DIS4-S5	Missense	SME<br>DS	Focal right fronto-temporal spikes with spreading (EEG)	(Iannetti et al., 2009; Nicita et al., 2010; Xu et al., 2014)
S243Y	DIS4-S5	Missense	SME	NR	(Depienne et al., 2008; Xu et al., 2014)
S243F	DIS4-S5	Missense	SME	Slow background, focal spikes and generalized epileptiform discharges (EEG)	(Lee et al., 2014)
V244L (Maternal)	DIS4-S5	Missense	DS	Myoclonic seizures and generalized spikes or spike-and-wave complexes in the interictal (EEG)	(Morimoto et al., 2006)
V244EfsX32 (De novo)	DIS4-S5	FrameShift	DS	NR	(Marini et al., 2007)
L247del	DIS4-S5	In-frame Deletion	Epilepsy	NR	(Lindy et al., 2018)
D249E	DIS4-S5	Missense	DS	Generalized tonic seizures; Absences<br>Mental retardation	(Le Gal et al., 2014)

V250I	DIS4-S5	Missense	Epilepsy	NR	(Retterer et al., 2016; Lindy et al., 2018)
M251X	DIS5	Nonsense	SME	NR	(Xu et al., 2014)
I252N	DIS5	Missense	DS	NR	(Ceulemans et al., 2004)
I252M	DIS5	Missense	Epilepsy	NR	(Wang et al., 2012)
T254CfsX4	DIS5	FrameShift	DS	Moderate intellectual disability<br>Slow speaking<br>Spasticity<br>Ataxia	(Martin et al., 2010)
T254I	DIS5	Missense	FS+	NR	(Zuberi et al., 2011)
F256LfsX20	DIS5	FrameShift	Epilepsy	NR	(Hesse et al., 2018)
C257R	DIS5	Missense	SME	NR	(Xu et al., 2014; Lindy et al., 2018)
S259R (De novo)	DIS5	Missense	DS	NR	(Sun et al., 2010; Wang et al., 2012)

F261IfsX16 (De novo)	DIS5	FrameShift	DS	NR	(Yang et al., 2017)
L263V (Paternal)	DIS5	Missense	FHM	Shift steady-state activation to more negative values<br>Slighty resists to entry into the fast inactivation state and slow inactivated state<br>Accelerated recovery from inactivation	(Kahlig et al., 2008; Castro et al., 2009; Barros et al., 2014)
K263X (De novo)	DIS5	Nonsense	DS	NR	(Zuberi et al., 2011)
I264fs	DIS5	FrameShift	SME	NR	(Lee et al., 2014)
I264T	DIS5	Missense	Epilepsy	NR	(Lindy et al., 2018)
G265W (De novo)	DIS5	Missense	DS	NR	(Depienne et al., 2008)
Q267X (De novo)	DIS5	Nonsense	DS	NR	(Wang et al., 2012)
G271R	DIS5	Missense	DS	NR	(Cetica et al., 2017)

L273Q	DIS5	Missense	DS	NR	(Cho et al., 2018)
L273P	DIS5	Missense	Epilepsy	NR	(Lindy et al., 2018)
N275K	DIS5	Missense	PEFS+	Hippocampa volume loss (MRI)	(Kim et al., 2014)
K276VfsX2	DIS5	FrameShift	DS	NR	(Xu et al., 2015)
K276Q	DIS5	Missense	DS	NR	(Xu et al., 2015)
K276I	DIS5	Missense	Epilepsy	NR	(Lindy et al., 2018)
C277R (De novo)	DIS5	Missense	DS	NR	(Depienne et al., 2008)
C277G	DIS5-S6	Missense	DS	NR	(Pérez Delgado et al., 2010)
Q279X (De novo)	DIS5-S6	Nonsense	SMEB	NR	(Zuberi et al., 2011)
W280R (De novo)	DIS5-S6	Missense	DS	Febrile seizures<br>Status epilepticus<br>Myoclonic	(Nabbout et al., 2003; Wang et al., 2012; Liu et al., 2018)

				Multifocal discharges (EEG)	
W280C	DIS5-S6	Missense	DS	Sporadic, diffuse, irregular spike-wave complex (EEG)	(Miyama et al., 2008; Villeneuve et al., 2014)
W281R (De novo)	DIS5-S6	Missense	SME	NR	(Xu et al., 2014)
P281S	DIS5-S6	Missense	DS	NR	(Depienne et al., 2008)
P281A	DIS5-S6	Missense	DS	NR	(Wang et al., 2012)
P281L (De novo)	DIS5-S6	Missense	DS	Moderate mental retardation	(Depienne et al., 2008; Gokben et al., 2017; Lindy et al., 2018)
S286WfsX6 (De novo)	DIS5-S6	FrameShift	DS	NR	(Nabbout et al., 2003; Riva et al., 2009)
E289X (De novo, Maternal)	DIS5-S6	Nonsense	DS	NR	(Nabbout et al., 2003; Orrico et al., 2009)
E289V	DIS5-S6	Missense	DS	Generalized tonic-clonic seizures<br>Developmental delay	(Lemke et al., 2012)

H290R	DIS5-S6	Missense	DS	NR	(Wang et al., 2012)
S291G	DIS5-S6	Missense	GEFS+	NR	(Zuberi et al., 2011)
T297I (Maternal)	DIS5-S6	Missense	DS	In association with R1525Q provokes shift in steady state activation to more positive values	(Nabbout et al., 2003; Binini et al., 2017)
V298EfsX3	DIS5-S6	FrameShift	SME<br>DS	Haploinsufficiency	(Zuberi et al., 2011; Petrelli et al., 2012)
N299fsX3	DIS5-S6	FrameShift	SME	NR	(Xu et al., 2014)
N306Metfs X26	DIS5-S6	FrameShift	DS	NR	(Wang et al., 2012)
F310fsX1	DIS5-S6	FrameShift	SME	NR	(Xu et al., 2014)
E311ValfsX 22	DIS5-S6	FrameShift	DS	NR	(Xu et al., 2015)
E311X (De novo)	DIS5-S6	Nonsense	DS	Haploinsufficiency	(Orrico et al., 2009)

F312S	DIS5-S6	Missense	DS	NR	(Ishii et al., 2017)
D313fs	DIS5-S6	FrameShift	SME	NR	(Lee et al., 2014)
W314R (Maternal)	DIS5-S6	Missense	Myoclonic-atonic epilepsy	NR	(Zuberi et al., 2011)
W314G	DIS5-S6	Missense	GEFS+	NR	(Afawi et al., 2016)
W314X	DIS5-S6	Nonsense	Epilepsy	NR	(Lindy et al., 2018)
Y317H	DIS5-S6	Missense	SME	NR	(Lee et al., 2014)
Y317C	DIS5-S6	Missense	DS	NR	(Cetica et al., 2017)
D320_R322 delinsG (De novo)	DIS5-S6	Slice donor site	DS	NR	(Depienne et al., 2008)
S321X (De novo)	DIS5-S6	Nonsense	DS	NR	(Depienne et al., 2008)

R322I	DIS5-S6	Missense	DS	NR	(Marini et al., 2007; Depienne et al., 2008)
Y323X (De novo)	DIS5-S6	Nonsense	DS	NR	(Riva et al., 2009; Nicita et al., 2010)
H324N	DIS5-S6	Missense	DS	NR	(Ishii et al., 2017)
H324P (Mosaic, Maternal)	DIS5-S6	Missense	DS	NR	(Yang et al., 2017)
Y325C (De novo)	DIS5-S6	Missense	SME	NR	(Liu et al., 2013)
Y325del	DIS5-S6	In-Frame Deletion	DS	NR	(Wu et al., 2015)
Y325X	DIS5-S6	Nonsense	SME	NR	(Liu et al., 2013)
L327R (Mosaic)	DIS5-S6	Missense	DS	Generalized tonic-clonic seizures	(de Lange et al., 2018)
E328GfsX12	DIS5-S6	FrameShift	DS	Generalized tonic-clonic seizures<br>Hemiconvulsion<br>Absences.	(de Lange et al., 2018)

G329C	DIS5-S6	Missense	Epilepsy	NR	(Lindy et al., 2018)
G329A (De novo)	DIS5-S6	Missense	GEFS+	Generalized tonic–clonic	(Myers et al., 2017a)
L331fsX (De novo)	DIS5-S6	FrameShift	SME<br>SMEB	NR	(Zuberi et al., 2011; Verbeek et al., 2013)
L331FefsX9 (De novo)	DIS5-S6	FrameShift	DS	NR	(Riva et al., 2009)
D332G	DIS5-S6	Missense	Epilepsy	NR	(Lindy et al., 2018)
C336Y	DIS5-S6	Missense	SME<br>DS	NR	(Xu et al., 2014)
G337E	DIS5-S6	Missense	DS	NR	(Ishii et al., 2017)
S340F	DIS5-S6	Missense	DS	NR	(Depienne et al., 2008)
A342S	DIS5-S6	Missense	DS	NR	(Xu et al., 2015)

A342V (De novo)	DIS5-S6	Missense	DS<br>SME	NR	(Zuberi et al., 2011; Xu et al., 2014)
G343R	DIS5-S6	Missense	DS	NR	(Ishii et al., 2017)
G343C	DIS5-S6	Missense	DS	NR	(Ishii et al., 2017)
G343E (De novo)	DIS5-S6	Missense	SMEB<br>SME<br>DS	Spike-wave complex and multifocal spikes (EEG)<br>Photo and pattern sensivity	(Fujiwara, 2003; Depienne et al., 2008; Zuberi et al., 2011)
C345VfsX8	DIS5-S6	FrameShift	DS	NR	(Harkin et al., 2007; Depienne et al., 2008)
C345R (De novo)	DIS5-S6	Missense	DS	NR	(Depienne et al., 2008)
C345Y	DIS5-S6	Missense	DS	Moderate intellectual disability<br>Slow speaking<br>Dysarthrophonia<br>Bradykinesia<br>Ataxia	(Martin et al., 2010)
C345X (De novo)	DIS5-S6	Nonsense	DS	NR	(Zuberi et al., 2011)
C345W (De novo)	DIS5-S6	Missense	DS	NR	(Zuberi et al., 2011)

G348X (De novo)	DIS5-S6	Nonsense	DS	NR	(Riva et al., 2009)
M350VfsX6 (De novo)	DIS5-S6	FrameShift	DS	NR	(Harkin et al., 2007)
M350V	DIS5-S6	Missense	DS	NR	(Xu et al., 2015)
C351R	DIS5-S6	Missense	DS	NR	(Doccini et al., 2015)
C351W (De novo)	DIS5-S6	Missense	DS	NR	(Zuberi et al., 2011)
C351X	DIS5-S6	Nonsense	DS	NR	(Kwong et al., 2012)
V352CfsX5	DIS5-S6	FrameShift	Epilepsy	NR	(Lindy et al., 2018)
K353fsX (De novo)	DIS5-S6	FrameShift	DS	NR	(Zuberi et al., 2011)
V352EfsX4 (De novo)	DIS5-S6	FrameShift	SME<br>DS	NR	(Harkin et al., 2007; Xu et al., 2014)

G355D (De novo)	DIS5-S6	Missense	DS	NR	(Depienne et al., 2008)
R356G	DIS5-S6	Missense	SMEB	NR	(Marini et al., 2007)
R356X	DIS5-S6	Nonsense	DS	NR	(Ishii et al., 2017)
N357Y	DIS5-S6	Missense	DS	NR	(Cho et al., 2018)
N357I (De novo)	DIS5-S6	Missense	DS	NR	(Depienne et al., 2008)
P358T	DIS5-S6	Missense	SMEB<br>SME	NR	(Marini et al., 2007; Xu et al., 2014)
P358S	DIS5-S6	Missense	DS	NR	(Xu et al., 2014)
N359S (De novo)	DIS5-S6	Missense	DS	NR	(Zuberi et al., 2011; Dhamija et al., 2014; Xu et al., 2014; Butler et al., 2017a)
N359IfsX20	DIS5-S6	FrameShift	DS	NR	(Wang et al., 2012)
N359I	DIS5-S6	Missense	Epilepsy	NR	(Lindy et al., 2018)

N359K (De novo)	DIS5-S6	Missense	DS	NR	(Yang et al., 2017)
T363P	DIS5-S6	Missense	DS	NR	(Zuberi et al., 2011)
Thr363Arg	DIS5-S6	Missense	DS	Generalized tonic-clonic seizures	(Zuberi et al., 2011; Le Gal et al., 2014)
S364AfsX15 (De novo)	DIS5-S6	FrameShift	DS	NR	(Zuberi et al., 2011)
F365LfsX14 (De novo)	DIS5-S6	Missense	DS	NR	(Yang et al., 2017)
D366X (De novo)	DIS5-S6	Nonsense	DS	NR	(Depienne et al., 2008)
D366fsX76	DIS5-S6	FrameShift	SME	NR	(Xu et al., 2014)
D366E (De novo)	DIS5-S6	Missense	DS	Generalized tonic-clonic seizures	(Zucca et al., 2008)
A371V	DIS5-S6	Missense	Epilepsy	NR	(Lindy et al., 2018)

L373FfsX6	DIS5-S6	FrameShift	PEFS+	Mild ventricular dilatation (MRI)<br>Developmental delay	(Kim et al., 2014)
S374fsX5 (De novo)	DIS5-S6	FrameShift	DS	NR	(Nabbout et al., 2003)
S374Y (De novo)	DIS5-S6	Missense	DS	NR	(Djémié et al., 2016)
L375F (De novo)	DIS5-S6	Missense	DS	NR	(Wei et al., 2018)
R377X (De novo)	DIS5-S6	Nonsense	DS	NR	(Depienne et al., 2008; Riva et al., 2009; Zuberi et al., 2011; Wang et al., 2012; Gaily et al., 2013; Xu et al., 2014; Djémié et al., 2016; Esterhuizen et al., 2018)
R377L	DIS5-S6	Missense	SME	NR	(Hattori et al., 2008)
R377Q (Maternal)	DIS5-S6	Missense	GEFS+	Generalized tonic-clonic seizures	(Zucca et al., 2008; Xu et al., 2015; Cetica et al., 2017)
L378Q	DIS5-S6	Missense	DS	NR	(Depienne et al., 2008)
M379R	DIS5-S6	Missense	DS	Generalized tonic-clonic seizures<br>Partial seizures<br>Developmental delay	(Lemke et al., 2012)

M379K	DIS5-S6	Missense	DS	NR	(Xu et al., 2015)
T380IfsX11	DIS5-S6	FrameShift	Epilepsy	NR	(Lindy et al., 2018)
D382N (De novo)	DIS5-S6	Missense	SME	NR	(Zuberi et al., 2011)
D382R	DIS5-S6	Missense	DS	NR	(Xu et al., 2014)
F383D (De novo)	DIS5-S6	Missense	DS	NR	(Riva et al., 2009)
W384R (De novo)	DIS5-S6	Missense	DS<br>SMEB<br>SME	Generalized tonic-clonic seizures<br>Partial seizures	(Zuberi et al., 2011; Wang et al., 2012; Verbeek et al., 2013)
W384X	DIS5-S6	Nonsense	DS	Complex partial seizures with generalization<br>Tonic seizures	(Surovy et al., 2016)
E385K	DIS5-S6	Missense	SMEB	NR	(Harkin et al., 2007)
E385G(De novo)	DIS5-S6	Missense	DS	NR	(Petrelli et al., 2012; Ko et al., 2018)

E385V	DIS5-S6	Missense	DS	NR	(Doccini et al., 2015)
E385D (De novo)	DIS5-S6	Missense	DS	NR	(Wu et al., 2015)
N386KfsX64 (De novo)	DIS5-S6	FrameShift	DS	NR	(Wang et al., 2012)
Y388H (Paternal)	DIS5-S6	Missense	DS<br>GEFS+	NR	(Mahoney et al., 2009; Bennett et al., 2017)
Q389X	DIS5-S6	Nonsense	DS	NR	(Nabbout et al., 2003)
T391P (De novo)	DIS5-S6	Missense	DS	Generalized tonic-conic seizures<br>Partial Seizures	(Reyes et al., 2011)
R393TfsX57	DIS5-S6	FrameShift	DS	NR	(Zuberi et al., 2011)
L392F	DIS5-S6	Missense	DS	NR	(Ishii et al., 2017)
R393C (De novo)	DIS5-S6	Missense	DS<br>LGS<br>SMEB	NR	(Harkin et al., 2007; Marini et al., 2007; Riva et al., 2009; Lim et al., 2011; Zuberi et al., 2011; Kwong et al., 2012; Allen et al., 2013; Villeneuve et al., 2014)

R393S (De novo)	DIS5-S6	Missense	DS<br>Myoclonic astatic epilepsy	NR	(Harkin et al., 2007; Marini et al., 2007; Depienne et al., 2008; Riva et al., 2009)
R393H (De novo)	DIS5-S6	Missense	DS<br>SMEB	Generalized tonic-clonic seizures<br>Myoclonus<br>Febrile seizures<br>Developmental delay	(Claes et al., 2003; Marini et al., 2007; Sun et al., 2010; Zuberi et al., 2011; Lemke et al., 2012; Rilstone et al., 2012; Wang et al., 2012; Xu et al., 2014; Djémié et al., 2016; Haginoya et al., 2018)
R393L	DIS5-S6	Missense	DS	NR	(Cetica et al., 2017)
A394D	DIS5-S6	Missense	GEFS+	Mild proeminece of lateral ventricles (MRI)<br>Developmental delay<br>Generalized tonic-clonic seizures	(Myers et al., 2017a)
A395SfsX55	DIS5-S6	FrameShift	DS	NR	(Xu et al., 2015)
A395P (De novo)	DIS5-S6	Missense	Cryptogenic focal epilepsy	NR	(Harkin et al., 2007)
A395MfsX6 (De novo)	DIS5-S6	FrameShift	DS	NR	(Sun et al., 2010)
G396E (De novo)	DIS5-S6	Missense	DS	NR	(Lim et al., 2011)

T398RfsX3	DIS5-S6	FrameShift	Epilepsy	NR	(Lindy et al., 2018)
Y399D	DIS5-S6	Missense	Epilepsy	NR	(Lindy et al., 2018)
Y399F	DIS5-S6	Missense	GEFS+	Focal seizures with secondary generalization arising from hemispheres (EEG)	(Gauthier et al., 2017)
Y399X (De novo)	DIS5-S6	Nonsense	DS	NR	(Harkin et al., 2007)
M400DfsX 49	DIS5-S6	Missense	DS	NR	(Xu et al., 2015)
M400del (De novo)	DIS6	In-frame Deletion	SME<br>DS<br>SMEB	NR	(Ceulemans et al., 2004; Depienne et al., 2008; Wang et al., 2012)
M400K	DIS6	Missense	DS	NR	(Xu et al., 2015)
M400V	DIS6	Missense	GEFS+	NR	(Brunklaus et al., 2015)
F402L (Mosaic)	DIS6	Missense	Epilepsy	NR	(Stosser et al., 2018)

F403L (De novo)	DIS6	Missense	DS	NR	(Harkin et al., 2007)
V406F	DIS6	Missense	DS	NR	(Zuberi et al., 2011)
V404CfsX46	DIS6	FrameShift	DS	NR	(Ream and Mikati, 2014; Mahdieh et al., 2018)
F408L	DIS6	Missense	Epilepsy	NR	(Klassen et al., 2011)
L409W(Maternal)	DIS6	Missense	DS	NR	(Wang et al., 2012)
G408WfsX6	DIS6	FrameShift	DS	NR	(Cetica et al., 2017)
F412I (Paternal)	DIS6	Missense	SMEB GEFS+	Febrile seizure	(Ebrahimi et al., 2010; Tonekaboni et al., 2013)
Y413N (De novo)	DIS6	Missense	DS	NR	(Harkin et al., 2007; Mulley et al., 2013)
I415K (De novo)	DIS6	Missense	Epilepsy	NR	(Staněk et al., 2018)

N416IfsX2	DIS6	FrameShift	DS	NR	(Zuberi et al., 2011)
N416KfsX2	DIS6	FrameShift	Epilepsy	NR	(Lindy et al., 2018)
N416I	DIS6	Missense	DS	Focal spike-wave (EEG)	(Zhou et al., 2018)
L417X	DIS6	Nonsense	DS	NR	(Ishii et al., 2017)
A420WfsX 27	DIS6	FrameShift	DS	NR	(Zuberi et al., 2011)
A420V (De novo)	DIS6	Missense	GEFS+	NR	(Xu et al., 2015)
A420D	DIS6	Missense	Epilepsy	NR	(Butler et al., 2017a)
V421M	DIS6	Missense	DS	NR	(Trujillano et al., 2017)
V422M(De novo)	DIS6	Missense	DS	NR	(Kwong et al., 2012; Butler et al., 2017a)
V422Le(De novo)	DIS6	Missense	EE	Psychomotor developmental delay	(Ohashi et al., 2014)

				Theta activies with right predominance (EEG)	
V422E(De novo)	DIS6	Missense	Cryptogenic generalized epilepsy	NR	(Harkin et al., 2007)
V422A	DIS6	Missense	DS	NR	(Xu et al., 2015)
Y426N(De novo)	DI-DII	Missense	DS	Reduced current density<br>Shift steady state inactivation to more negative values<br>Larger time constant for the slow component of recovery from fast inactivation	(Nabbout et al., 2003; Ohmori et al., 2006; Allen et al., 2016)
Y426C (De novo)	DI-DII	Missense	DS	NR	(Depienne et al., 2008; Wang et al., 2012)
Y426X	DI-DII	Nonsense	DS	NR	(Hesse et al., 2018)
E427K	DI-DII	Missense	DS	NR	(Cho et al., 2018)
E431X	DI-DII	Nonsense	DS	NR	(Zuberi et al., 2011)
A432S	DI-DII	Missense	DS	NR	(Usluer et al., 2016)

L433fsX16 (De novo)	DI-DII	FrameShift	Myoclonic astatic epilepsy	Severe idiopathic generalized epilepsy of infancy with generalized tonic-clonic seizures	(Ebach et al., 2005)
L434WfsX14	DI-DII	FrameShift	DS	NR	(Xu et al., 2015)
E435X (De novo)	DI-DII	Nonsense	DS	Mild psychomotor retardation<br>Myoclonic seizures<br>Atypical absence	(Fukuma et al., 2004; Wang et al., 2012)
Q439X (De novo)	DI-DII	Nonsense	DS	NR	(Depienne et al., 2008; Wang et al., 2012)
E443X	DI-DII	Nonsense	DS	Multifocal Discharges<br>Generalized polyspike-wave (EEG)	(Liu et al., 2018)
Q445X	DI-DII	Nonsene	DS	NR	(Surovy et al., 2016)
I448X (De novo)	DI-DII	Nonsense	DS	NR	(Wallace et al., 2003)
E449X	DI-DII	Nonsense	DS	NR	(Xu et al., 2015)
E449DfsX41 (De novo)	DI-DII	FrameShift	Epilepsy	NR	(Lindy et al., 2018)

Q450X (De novo)	DI-DII	Nonsene	DS	NR	(Depienne et al., 2008; Kwong et al., 2012; Mercimek-Mahmutoglu et al., 2015)
E450GfsX10 (De novo)	DI-DII	FrameShift	DS	NR	(Orrico et al., 2009)
Q459R(De novo)	DI-DII	Missense	DS<br>GEFS+	NR	(Møller et al., 2016)
Q460X	DI-DII	Nonsense	DS	NR	(Zuberi et al., 2011)
S470FfsX17 (De novo)	DI-DII	FrameShift	DS	NR	(Depienne et al., 2008)
S470fsX15 (De novo)	DI-DII	FrameShift	DS	NR	(Sun et al., 2010)
G477AfsX13	DI-DII	FrameShift	Epilepsy	NR	(Lindy et al., 2018)
D481AfsX8	DI-DII	FrameShift	SME	NR	(Xu et al., 2015)
S487EfsX6 (Mosaic)	DI-DII	FrameShift	DS	Generalized tonic-clonic seizure<br>Focal seizures with secondary generalization<br>Mild motor and cognitive delay	(Sharkia et al., 2016)

K488EfsX6 (Maternal)	DI-DII	FrameShift	DS	NR	(Yang et al., 2017)
R498X (De novo)	DI-DII	Nonsense	DS	NR	(Depienne et al., 2008)
R500EfsX10 (De novo)	DI-DII	FrameShift	DS	NR	(Wang et al., 2012)
R500EfsX (De novo)	DI-DII	FrameShift	DS	NR	(Wang et al., 2012)
R500Q	DI-DII	Missense	DS	NR	(Zhou et al., 2018)
R501fsX43 (De novo)	DI-DII	FrameShift	SMEB	NR	(Ohmori et al., 2002)
R504TfsX12	DI-DII	FrameShift	SME	NR	(Villeneuve et al., 2014)
K505X (De novo)	DI-DII	Nonsense	DS	NR	(Liu et al., 2018)
Q506X	DI-DII	Nonsense	SME<br>DS	NR	(Cantarín Extremera et al., 2011)
K507AfsX9	DI-DII	FrameShift	Epilepsy	NR	(Lindy et al., 2018)

K507SfsX36	DI-DII	FrameShift	SME	NR	(Villeneuve et al., 2014)
Q509X (De novo)	DI-DII	Nonsense	Epilepsy	NR	(Staněk et al., 2018)
E511fsX543	DI-DII	FrameShift	DS	NR	(Wang et al., 2012)
E513KfsX31 (Paternal, Mosaic)	DI-DII	FrameShift	DS	NR	(Verbeek et al., 2011)
E513X	DI-DII	Nonsense	DS	NR	(Depienne et al., 2008)
E513GfsX5	DI-DII	FrameShift	Epilepsy	NR	(Lindy et al., 2018)
E524X	DI-DII	Nonsense	Epilepsy	NR	(Lindy et al., 2018)
S525F(De novo)	DI-DII	Missense	DS	NR	(Depienne et al., 2008)
Y525LfsX2	DI-DII	FrameShift	DS	NR	(Cetica et al., 2017)

I529SfsX15	DI-DII	FrameShift	DS	NR	(Ishii et al., 2017)
l529NfsX11	DI-DII	FrameShift	DS	NR	(Cetica et al., 2017)
R530KfsX10	DI-DII	FrameShift	DS	NR	(Haginoya et al., 2018)
G533VfsX11	DI-DII	FrameShift	DS	NR	(Ishii et al., 2017)
R542X (De novo)	DI-DII	Nonsense	SME DS	NR	(Marini et al., 2007; Depienne et al., 2008; Riva et al., 2009; Sun et al., 2010; Zuberi et al., 2011)
R542Q(Paternal)	DI-DII	Missense	GEFS+ SME	NR	(Escayg et al., 2001; Weiss et al., 2003; Combi et al., 2009; Orrico et al., 2009; Wang et al., 2012; Lee et al., 2014; Lal et al., 2016)
L543X	DI-DII	Nonsense	DS	NR	(Cetica et al., 2017)
T544HfsX14 (De novo)	DI-DII	FrameShift	SMEB	NR	(Lim et al., 2011)
Y545LfsX12	DI-DII	FrameShift	DS	NR	(Ishii et al., 2017)

E546X	DI-DII	Nonsense	DS	NR	(Liu et al., 2018)
Y549GfsX10	DI-DII	FrameShift	DS	NR	(Zuberi et al., 2011)
K547EfsX23	DI-DII	FrameShift	DS	NR	(Harkin et al., 2007; Depienne et al., 2008)
R548EsX23 (De novo)	DI-DII	FrameShift	SMEB	NR	(Ohmori et al., 2002)
Q554fsX17	DI-DII	FrameShift	DS	NR	(Sun et al., 2010)
E554R	DI-DII	Missense	SME	NR	(Xu et al., 2014)
Q554H (De novo)	DI-DII	Missense	DS	Genelized tonic-clonic seizure, atonic and myoclonic seizures	(Skjei et al., 2015)
S555FfsX6 (De novo)	DI-DII	FrameShift	DS	NR	(Zuberi et al., 2011)
L556CfsX2 (De novo)	DI-DII	FrameShift	DS	NR	(Sun et al., 2008a)

R560VfsX63	DI-DII	FrameShift	Epilepsy	Generalized epilepsy<br>Developmental delay	(Hesse et al., 2018)
R560PfsX8 (De novo)	DI-DII	FrameShift	DS	NR	(Zuberi et al., 2011)
L563YfsX60 (De novo)	DI-DII	FrameShift	DS	NR	(Harkin et al., 2007)
L563Q	DI-DII	Missense	Episodic ataxia	NR	(Choi et al., 2017)
P566S	DI-DII	Missense	DS	NR	(Usluer et al., 2016)
R568X (De novo)	DI-DII	Nonsense	DS	NR	(Ohmori et al., 2002; Marini et al., 2007; Zuberi et al., 2011; Okumura et al., 2012; Villeneuve et al., 2014; Kothur et al., 2018)
R568Q	DI-DII	Missense	NR	Seizures	(Butler et al., 2017a)
S570N	DI-DII	Missense	EE	NR	(Posey et al., 2017)
S570I (Familial)	DI-DII	Missense	GEFS+	NR	(Cetica et al., 2017)

F575SfsX48 (De novo)	DI-DII	FrameShift	Cryptogenic focal epilepsy<br>PEFS+	NR	(Harkin et al., 2007; Kim et al., 2014)
R580X (Familial)	DI-DII	Nonsense	DS	NR	(Depienne et al., 2008; Arlier et al., 2010; Zuberi et al., 2011)
K582SfsX7	DI-DII	FrameShift	Epilepsy	NR	(Lindy et al., 2018)
E602X	DI-DII	Nonsense	DS	NR	(Depienne et al., 2008)
R604H (Paternal)	DI-DII	Missense	DS<br>Juvenile myoclonic epilepsy	Generalizad tonic-clonic seizures and Febrile Seizures	(Escayg et al., 2001; Harkin et al., 2007; Fendri-Kriaa et al., 2009; Wang et al., 2012; Lal et al., 2016)
S607PfsX16	DI-DII	FrameShift	Epilepsy	NR	(Lindy et al., 2018)
L608CfsX15	DI-DII	FrameShift	SME<br>SMEB	NR	(Ohmori et al., 2002; Depienne et al., 2008)
R612X (De novo)	DI-DII	Nonsense	DS	NR	(Sun et al., 2008a; Xu et al., 2014)
R613X (De novo)	DI-DII	Nonsense	SME<br>DS	NR	(Kearney et al., 2006; Depienne et al., 2008; Riva et al., 2009; Zuberi et al., 2011; Rodda et al., 2012; Wang et al., 2012; Moehring et al., 2013; Lee et al., 2014; Xu et al., 2014)

E616RfsX7	DI-DII	FrameShift	DS	NR	(Cetica et al., 2017)
E616D	DI-DII	Missense	Epilepsy	NR	(Parrini et al., 2017)
R617TfsX10 (Paternal)	DI-DII	FrameShift	DS	NR	(Yang et al., 2017)
R618C (Paternal)	DI-DII	Missense	PEFS+	Generalized tonic-clonic seizures<br>Multifocal epilepsy (EEG)<br>Bilateral bursts of 3-4 Hz spike and wave (EEG)	(Brunklaus et al., 2015)
R618AfsX5 (Paternal)	DI-DII	FrameShift	Epilepsy	NR	(Lindy et al., 2018)
S620AfsX5 (De novo)	DI-DII	FrameShift	SMEB	NR	(Marini et al., 2007)
T625PfsX47 (De novo)	DI-DII	FrameShift	DS	NR	(Depienne et al., 2008)
S626G	DI-DII	Missense	SME<br>SMEB<br>Cryptogenic generalized epilepsy	NR	(Harkin et al., 2007; Depienne et al., 2008; Xu et al., 2014; Lee et al., 2017)

R627K	DI-DII	Missense	GEFS+	NR	(Cetica et al., 2017)
H642TfsX30 (De novo)	DI-DII	FrameShift	DS	NR	(Ishii et al., 2017)
V645GfsX30 (De novo)	DI-DII	FrameShift	DS	NR	(Yang et al., 2017; Liu et al., 2018)
N648Lys	DI-DII	Missense	EE	NR	(Zhu et al., 2017)
P657L	DI-DII	Missense	DS	NR	(Xu et al., 2015)
S662X (De novo)	DI-DII	Nonsense	PEFS+	Generalized tonic-clonic seizures	(Yu et al., 2010)
D674G (De novo)	DI-DII	Missense	DS	NR	(Harkin et al., 2007)
T685LfsX5	DI-DII	FrameShift	SMEB	NR	(Zuberi et al., 2011)
K691X	DI-DII	Nonsense	SME	NR	(Xu et al., 2014)

S695TfsX7 (De novo)	DI-DII	FrameShift	DS	NR	(Depienne et al., 2008)
S696SfsX8 (De novo)	DI-DII	FrameShift	SME	NR	(Lim et al., 2011)
H698Y	DI-DII	Missense	SME	NR	(Villeneuve et al., 2014)
S700fsX4	DI-DII	FrameShift	SME	NR	(Xu et al., 2014)
D702AfsX25 (De novo)	DI-DII	FrameShift	EE	NR	(de Kovel et al., 2016)
P707NfsX9 (De novo)	DI-DII	FrameShift	DS	NR	(Kearney et al., 2006)
Q711X	DI-DII	Nonsense	SME	Slow background, generalized spikes (EEG)<br>Generalized tonic-clonic seizures	(Lee et al., 2014)
Q711HfsX4 (De novo)	DI-DII	FrameShift	DS	NR	(Depienne et al., 2008)
R712X (De novo; Maternal)	DI-DII	Nonsense	SME<br>DS	Focal epilepsy frontal right, beta activity (EEG)<br>Reduced current densitity, low protein expression	(Ohmori et al., 2002; Sugawara et al., 2002, 2003; Fukuma et al., 2004; Depienne et al., 2008; Zuberi et al., 2011; Okumura et al., 2012; Wang et al., 2012; Moehring et al., 2013)

W737X	DI-DII	Nonsense	DS	NR	(Ko et al., 2018)
W738X (De novo)	DI-DII	Nonsense	SME	Febrile seizures<br>Partial, generalized tonic-clonic, myoclonc<br>Severe intellectual disability	(Kwong et al., 2012; Xu et al., 2014)
W738L	DI-DII	Missense	SME	NR	(Xu et al., 2014)
W738CfsX9	DI-DII	FrameShift	SME	NR	(Hattori et al., 2008)
I747TfsX9	DI-DII	FrameShift	DS	NR	(Ishii et al., 2017)
W748X	DI-DII	Nonsense	DS	NR	(Wang et al., 2012)
Y753X	DI-DII	Nonsense	DS	NR	(Do et al., 2017)
W754X	DI-DII	Nonsense	DS	NR	(Ishii et al., 2017)
W754L	DI-DII	Missense	DS	NR	(Xu et al., 2015)

H759fsX14 (De novo)	DI-DII	FrameShift	SME	NR	(Lin et al., 2013)
N762D (Paternal)	DI-DII	Missense	DS	NR	(Zuberi et al., 2011)
P768L	DIIS1	Missense	SME<br>DS	NR	(Sun et al., 2010; Xu et al., 2014)
P768fsX5	DIIS1	FrameShift	DS	NR	(Sun et al., 2010)
F769Y	DIIS1	Missense	GEFS+	NR	(Cetica et al., 2017)
A773TfsX31 (De novo)	DIIS1	FrameShift	EE	NR	(Wei et al., 2018)
I774HfsX25	DIIS1	FrameShift	DS	NR	(Xu et al., 2015)
L780delinsFI	DIIS1	In-frame Deletion	Epilepsy	NR	(Lindy et al., 2018)
L783P (De novo)	DIIS1	Missense	DS	NR	(Harkin et al., 2007)
M785V	DIIS1-S2	Missense	DS	NR	(Xu et al., 2015)

M785T (De novo)	DIIS1-S2	Missense	DS	NR	(Zuberi et al., 2011; Lindy et al., 2018)
A786T	DIIS1-S2	Missense	DS	NR	(Cetica et al., 2017)
E788K	DIIS1-S2	Missense	SME	NR	(Hattori et al., 2008)
Y790C (Paternal)	DIIS1-S2	Missense	GEFS+	Reduced current density (91%) associeted with reduction of cell surface expression (91%)	(Annesi et al., 2003; Orrico et al., 2009; Bechi et al., 2015; Bennett et al., 2017)
Y790F	DIIS1-S2	Missense	GEFS+<br>Panayiotopoulos Syndrome	NR	(Grosso et al., 2007; Cetica et al., 2017)
Y790X (De novo)	DIIS1-S2	Nonsense	DS	NR	(Depienne et al., 2008)
Q791X (De novo)	DIIS1-S2	Nonsense	DS	NR	(Esterhuizen et al., 2018)
N804K	DIIS2	Missense	DS	NR	(Cetica et al., 2017)
F807LfsX11 (De novo)	DIIS2	FrameShift	DS	NR	(Zuberi et al., 2011)

T808S (De novo)	DIIS2	Missense	ICEGTC	Rare sharp waves in left temporal (EEG)<br>Moderate mental decline<br>Increase current density<br>Delayed recovery from inactivation	(Fujiwara, 2003; Rhodes et al., 2005)
T812I	DIIS2	Missense	SMEB	NR	(Zuberi et al., 2011)
T812R (De novo)	DIIS2	Missense	DS	NR	(Claes et al., 2009; Riva et al., 2009)
Y825fs	DIIS2	FrameShift	DS	NR	(Martin et al., 2010)
Y827D	DIIS2	Missense	DS	NR	(Xu et al., 2015)
E830X (De novo)	DIIS2	Nonsense	DS	NR	(Zhang et al., 2015)
W832X	DIIS3	Nonsense	DS	NR	(Depienne et al., 2008)
F835WfsX7 (De novo)	DIIS3	FrameShift	DS	NR	(Depienne et al., 2008)
D836X (De novo)	DIIS3	Nonsense	DS	NR	(Zuberi et al., 2011)

V841A	DIIS3	Missense	DS	NR	(Ko et al., 2018)
L842R	DIIS3	Missense	DS	NR	(Zuberi et al., 2011)
F843R	DIIS3	Missense	DS	NR	(Depienne et al., 2008)
S843X (De novo)	DIIS3	Nonsense	DS	EEG reveals focal spike activity, diffuse polyspike waves accompanied by myoclonic jerks	(Buoni et al., 2006)
S843R (De novo)	DIIS3	Missense	DS	NR	(Depienne et al., 2008)
L844X	DIIS3	Nonsense	SME	NR	(Dhamija et al., 2014)
E853K (De novo)	DIIS3	Missense	DS	NR	(Riva et al., 2009)
E846G	DIIS3	Missense	SME	NR	(Xu et al., 2014)
R846DfsX31	DIIS3	FrameShift	DS	NR	(Ishii et al., 2017)

G848DfsX29	DIIS3	FrameShift	DS	NR	(Depienne et al., 2008)
L849TfsX66	DIIS3	FrameShift	DS	NR	(Depienne et al., 2008)
L849P	DIIS3	Missense	DS	NR	(Cetica et al., 2017)
R851P (De novo)	DIIS3	Missense	DS	NR	(Esterhuizen et al., 2018)
E853K (De novo)	DIIS3-S4	Missense	DS	NR	(Riva et al., 2009)
G854_L855del (De novo)	DIIS3-S4	In-frame Deletion	SMEB	NR	(Zuberi et al., 2011)
G854fsX23 (De novo)	DIIS3-S4	FrameShift	DS	NR	(Harkin et al., 2007)
V857FfsX20	DIIS3-S4	FrameShift	SME	NR	(Depienne et al., 2008; Lindy et al., 2018)
V857I	DIIS3-S4	Missense	Epilepsy	NR	(Lindy et al., 2018)
R859C (De novo, Paternal)	DIIS4	Missense	GEFS+ DS	Shift steady state activation to more positive values	(Barela et al., 2006; Depienne et al., 2008; Zuberi et al., 2011)

R859H (Paternal)	DIIS4	Missense	GEFS+	Shift steady state activation and inactivation to more negative values<br>Increase Persistent current	(Volkers et al., 2011; Myers et al., 2017a; Lindy et al., 2018)
R862X (De novo, Familal)	DIIS4	Nonsense	GEFS+<br>SME<br>SMEB	NR	(Orrico et al., 2009; Wang et al., 2012; Lee et al., 2014; Xu et al., 2014)
R862G (De novo)	DIIS4	Missense	MMPSI	Multifocal epilepsy<br>Hemiclonic<br>Severe intellectual disability	(Carranza Rojo et al., 2011; Barba et al., 2014)
R862Q	DIIS4	Missense	DS	NR	(Arlier et al., 2010; Zuberi et al., 2011)
L863W (De novo)	DIIS4	Missense	SME	NR	(Xu et al., 2014)
L864RfsX13 (De novo)	DIIS4	FrameShift	DS	NR	(Zuberi et al., 2011)
R865G	DIIS4	Missense	DS	Shift steady state activation to more negative values<br>Increase Persistent current	(Volkers et al., 2011)
R865X (De novo)	DIIS4	Nonsense	DS	NR	(Ohmori et al., 2002; Depienne et al., 2008; Lim et al., 2011; Wang et al., 2012;

					Xu et al., 2014; Kothur et al., 2018)
K868del	DIIS4	In-frame Deletion	SME	NR	(Moehring et al., 2013)
K868fsX7	DIIS4	FrameShift	SME	NR	(Xu et al., 2014)
Ks868N (Maternal)	DIIS4	Missense	GEFS+	NR	(Cetica et al., 2017)
A870LfsX5 (De novo)	DIIS4	FrameShift	DS	NR	(Marini et al., 2007)
S872Y	DIIS4	Missense	SME	NR	(Xu et al., 2014)
W873X	DIIS4	Nonsense	SMEB	NR	(Villeneuve et al., 2014)
T875K (Paternal)	DIIS4	Missense	SME<br>GEFS+	Self-limited focal epilepsy of childhood<br>Atypical childhood epilepsy with centrotemporal spikes (EEG)<br>Panayiotopoulos syndrome	(Depienne et al., 2008; Kivity et al., 2017)
T875M	DIIS4	Missense	PEFS+<br>SMEB	Enhance slow inactivation in the presence of beta1 subunity	(Escayg et al., 2000; Alekov et al., 2001; Spampanato et al., 2001a; George, 2004; Wang et al., 2012)

				Increase persistent current<br>Shift steady state activation to more positive values	
L876I	DIIS4	Missense	DS	NR	(Wang et al., 2012)
L879X	DIIS4-S5	Nonsense	DS	NR	(Depienne et al., 2008)
L879P	DIIS4-S5	Missense	DS<br>GEFS+	NR	(Møller et al., 2016)
I880KfsX14	DIIS4-S5	FrameShift	SME	NR	(Depienne et al., 2008)
I882F (De novo)	DIIS4-S5	Missense	DS	NR	(Ishii et al., 2017)
S886F	DIIS4-S5	Missense	DS	NR	(Cetica et al., 2017)
Ala889LeufsX5 (De novo)	DIIS4-S5	FrameShift	DS	NR	(Zuberi et al., 2011)
Leu890Phe (De novo)	DIIS4-S5	Missense	DS	NR	(Zuberi et al., 2011; Tsai et al., 2018)

Asn892Ilefs X2 (De novo)	DIIS5	FrameShift	SME	NR	(Singh et al., 2009)
L893X (De novo)	DIIS5	Nonsense	DS	NR	(Riva et al., 2009)
T894S	DIIS5	Missense	Epilepsy	NR	(Lindy et al., 2018)
V896L (De novo)	DIIS5	Missense	DS	NR	(Depienne et al., 2008)
V896I	DIIS5	Missense	DS	Generalized tonic-clonic seizures	(Rilstone et al., 2012; Wang et al., 2012)
V896F	DIIS5	Missense	SMEB	NR	(Wang et al., 2012)
V896_A898 del	DIIS5	In-frame deletion	Epilepsy	NR	(Lindy et al., 2018)
L897S	DIIS5	Missense	DS	NR	(Xu et al., 2015)
I899T (De novo, Familial)	DIIS5	Missense	GEFS+ LGS	NR	(Orrico et al., 2009; Allen et al., 2013; Bennett et al., 2017)
F902C	DIIS5	Missense	DS	Not measurable current	(Ohmori et al., 2002; Rhodes et al., 2004)

A905T	DIIS5	Missense	DS	NR	(Ishii et al., 2017)
A905P (De novo)	DIIS5	Missense	EE	NR	(Hamdan et al., 2017)
V906GfsX29	DIIS5	FrameShift	DS	NR	(Ishii et al., 2017)
V907F	DIIS5	Missense	Focal epilepsy	Bitemporal slow waves (EEG)	(Hildebrand et al., 2016)
G908R	DIIS5	Missense	Epilepsy	NR	(Lindy et al., 2018)
Q910K	DIIS5	Missense	SME	NR	(Xu et al., 2014)
Q910L	DIIS5	Missense	DS	NR	(Ishii et al., 2017)
S915AfsX20 (De novo)	DIIS5-S6	FrameShift	SMEB	NR	(Kearney et al., 2006)
D918IfsX17	DIIS5-S6	FrameShift	DS	NR	(Sun et al., 2010)

M923I (Paternal)	DIIS5-S6	Missense	DS	NR	(Liu et al., 2018)
S925VfsX10	DIIS5-S6	FrameShift	Epilepsy	NR	(Lindy et al., 2018)
C927F (De novo)	DIIS5-S6	Missense	DS	NR	(Depienne et al., 2008)
Q928X	DIIS5-S6	Nonsense	Epilepsy	NR	(Lindy et al., 2018)
L929SfsX6	DIIS5-S6	FrameShift	DS	Episode of status epilepticus followed by persistent lethargy afted 48 hours of acute febrile	(Tsuji et al., 2011)
R931C (De novo)	DIIS5-S6	Missense	DS<br>SMEB	NR	(Ohmori et al., 2002; Depienne et al., 2008)
R931H	DIIS5-S6	Missense	SME<br>DS	Fever vaccination<br>Generalized tonic-clonic<br>Dyscognitive<br>Developmental regression after seizure onset	(Catarino et al., 2011; Zuberi et al., 2011; Rilstone et al., 2012; Xu et al., 2014)
R931P	DIIS5-S6	Missense	SME	NR	(Xu et al., 2014)
W932R (De novo)	DIIS5-S6	Missense	DS	NR	(Kothur et al., 2018)

W932C	DIIS5-S6	Missense	DS	NR	(Zuberi et al., 2011)
T932X (De novo)	DIIS5-S6	Nonsense	SME<br>DS	Generalized tonic-clonic seizures<br>Severe mental retardation	(Claes et al., 2003; Dhamija et al., 2014)
H933P	DIIS5-S6	Missense	DS	NR	(Zuberi et al., 2011)
M934R	DIIS5-S6	Missense	Epilepsy	NR	(Lindy et al., 2018)
M934I (De novo)	DIIS5-S6	Missense	DS	Moderate psychomotor retardation	(Fukuma et al., 2004; Depienne et al., 2008; Wang et al., 2012)
N935H (Paternal)	DIIS5-S6	Missense	PEFS+	Generalized tonic-clonic<br>Hemiclonic seizure<br>Multiple generalized slow waves with some spike discharges while awake, bilateral frontal spikes and spike waves during sleep (EEG)	(Sun et al., 2008b)
D936Y (De novo)	DIIS5-S6	Missense	Epilepsy	NR	(Lindy et al., 2018)
D936V (De novo)	DIIS5-S6	Missense	SME	NR	(Lin et al., 2013)

H939Y (De novo)	DIIS5-S6	Missense	DS	NR	(Sun et al., 2010)
H939P	DIIS5-S6	Missense	DS	NR	(Depienne et al., 2008)
H939Q (De novo)	DIIS5-S6	Missense	DS	Status epilepticus<br>Generalized tonic-clonic seizures<br>Complex partial seizures<br>Moderate mental retardation<br>No measurable current	(Claes et al., 2003; Ohmori et al., 2006)
S940F	DIIS5-S6	Missense	DS	NR	(Wang et al., 2012)
L942V	DIIS5-S6	Missense	DS<br>GEFS+	NR	(Møller et al., 2016)
L942P (De novo)	DIIS5-S6	Missense	DS	NR	(Riva et al., 2009)
I943CfsX10 (De novo)	DIIS5-S6	FrameShift	DS	NR	(Depienne et al., 2008)
I943D	DIIS5-S6	Missense	DS	NR	(Depienne et al., 2008)

V944A (De novo)	DIIS5-S6	Missense	SMEB	NR	(Fukuma et al., 2004; Wang et al., 2012)
V944E (De novo)	DIIS5-S6	Missense	DS	NR	(Harkin et al., 2007; Rodda et al., 2012)
F945L (De novo)	DIIS5-S6	Missense	DS	NR	(Harkin et al., 2007)
R946fsX8 (De novo)	DIIS5-S6	FrameShift	DS	NR	(Fujiwara, 2003)
R946C (De novo)	DIIS5-S6	Missense	SME<br>DS<br>SMEB	Non-functional Channel	(Fukuma et al., 2004; Volkers et al., 2011; Zuberi et al., 2011; Wang et al., 2012; Lee et al., 2014; Xu et al., 2014)
R946S (De novo)	DIIS5-S6	Missense	Severe idiopathic generalized epilepsy of infancy	Short generalized tonic-clonic seizures at night<br>Seizure onset left temporo-parietal (EEG)<br>Tonic seizure right arm<br>Seizure onset left frontal<br>Seizure onset right frontocentral	(Ebach et al., 2005; Tiefes et al., 2019)
R946H (De novo)	DIIS5-S6	Missense	PEFS+<br>SMEB<br>DS	Non-functional Channel	(Fukuma et al., 2004; Harkin et al., 2007; Depienne et al., 2008; Liao et al., 2010; Verbeek et al., 2011, 2013; Volkers et al., 2011; Zuberi et al., 2011; Wang et al., 2012)
R946P	DIIS5-S6	Missense	SME	NR	(Xu et al., 2014)

C949Y (De novo)	DIIS5-S6	Missense	DS	NR	(Depienne et al., 2008)
C949S (De novo)	DIIS5-S6	Missense	SME	NR	(Depienne et al., 2008)
G950R (De novo)	DIIS5-S6	Missense	DS	NR	(Zuberi et al., 2011)
G950E (De novo)	DIIS5-S6	Missense	DS	NR	(Harkin et al., 2007; Rodda et al., 2012)
W952R (De novo)	DIIS5-S6	Missense	DS	NR	(Lim et al., 2011)
W952G (De novo)	DIIS5-S6	Missense	DS	NR	(Sun et al., 2008a; Xu et al., 2014)
W952X	DIIS5-S6	Nonsense	DS	Spikes in frontal-occipital; High voltage slow-waves (EEG)	(Fujiwara, 2003; Fukuma et al., 2004; Wang et al., 2012)
E954K	DIIS5-S6	Missense	DS	NR	(Arlier et al., 2010; Zuberi et al., 2011)
T955I	DIIS5-S6	Missense	DS	NR	(Ishii et al., 2017)
M956K (De novo)	DIIS5-S6	Missense	DS	NR	(Zuberi et al., 2011)

M956T (Familial)	DIIS5-S6	Missense	FS+	Reduced current density (80%) associecited with reduction of cell surface expression (74%)	(Bechi et al., 2015; Pippucci et al., 2015)
W957R	DIIS5-S6	Missense	DS	NR	(Cantarín Extremera et al., 2011)
W957L (De novo)	DIIS5-S6	Missense	DS	NR	(Marini et al., 2007; Zuberi et al., 2011)
D958TfsX16	DIIS5-S6	FrameShift	DS	NR	(Zuberi et al., 2011)
D958RfsX13	DIIS5-S6	FrameShift	DS	NR	(Xu et al., 2015)
C959VfsX15 (De novo)	DIIS5-S6	FrameShift	DS	NR	(Sugawara et al., 2002)
C959R (De novo)	DIIS5-S6	Missense	DS	Post trauma epilepsy<br>lateralized tonic-clonic seizures<br>Severe mental retardation<br>Non-functional Channel	(Claes et al., 2003; Ohmori et al., 2006)
C959Y (De novo)	DIIS5-S6	Missense	LGS<br>SME	NR	(Allen et al., 2013; Xu et al., 2014)
C959S (De novo)	DIIS5-S6	Missense	DS	NR	(Cetica et al., 2017; Wei et al., 2018)

C959X	DIIS5-S6	Nonsense	Epilepsy	NR	(Lindy et al., 2018)
M960V	DIIS5-S6	Missense	DS	Spike-waves complex, rare focal spikes (EEG)<br>Severe mental retardation	(Fujiwara, 2003)
M960T	DIIS5-S6	Missense	GEFS+	NR	(Zuberi et al., 2011)
M960R	DIIS5-S6	Missense	DS	NR	(Xu et al., 2015)
Q965X	DIIS5-S6	Nonsense	DS	NR	(Harkin et al., 2007)
Q965fsX	DIIS5-S6	FrameShift	DS	Multifocal discharges (EEG)	(Liu et al., 2018)
M967I (Maternal)	DIIS6	Missense	GEFS+	NR	(Selmer et al., 2008)
C968G (De novo)	DIIS6	Missense	EE	NR	(de Kovel et al., 2016)
C968F	DIIS6	Missense	DS	NR	(Xu et al., 2015)

C968X (De novo)	DIIS6	Nonsense	SME<br>DS	NR	(Depienne et al., 2008; Wang et al., 2012)
L969P (De novo)	DIIS6	Missense	DS	NR	(Do et al., 2017)
T970DfsX9	DIIS6	FrameShift	DS	NR	(Zuberi et al., 2011)
T970fsX972 (De novo)	DIIS6	FrameShift	DS	NR	(Riva et al., 2009)
V971I	DIIS6	Missense	DS	NR	(Cetica et al., 2017)
V971L (De novo)	DIIS6	Missense	DS	Generalized and unilateral tonic-clonic seizurez<br>Myoclonic seizures<br>Apneic spells	(Poryo et al., 2017)
M973V (Paternal)	DIIS6	Missense	Cryptogenic generalized epilepsy	NR	(Harkin et al., 2007)
M973K (De novo)	DIIS6	Missense	DS	NR	(Depienne et al., 2008)
V975L	DIIS6	Missense	NR	Seizures and developmental delay	(Butler et al., 2017b)

M976RfsX1	DIIS6	FrameShift	DS	Myoclonic, atonic, focal or motor seizures	(Skjei et al., 2015)
M976I (De novo, Paternal)	DIIS6	Missense	GEFS+<br>DS	Generalized tonic-clonic seizures<br>Focal Seizures<br>Hemiconvulsions	(Orrico et al., 2009; Zuberi et al., 2011; Petrelli et al., 2012; Bennett et al., 2017)
V977M	DIIS6	Missense	DS	NR	(Xu et al., 2015)
G979R	DIIS6	Missense	ICEGTC	Bilateral frontal sharp wave, Spike-slow (EEG)	(Fujiwara, 2003; Rhodes et al., 2005)
G979V	DIIS6	Missense	SMEB	NR	(Zuberi et al., 2011)
G979E	DIIS6	Missense	DS	NR	(Xu et al., 2015)
N980S	DIIS6	Missense	Epilepsy	NR	(Lindy et al., 2018)
L981P	DIIS6	Missense	DS	NR	(Ishii et al., 2017)
V982L (De novo)	DIIS6	Missense	SMEB<br>Acute encephalopathy with biphasic seizures and	Focal epilepsy<br>Spastic quadriplegia<br>Severe mental retardation	(Singh et al., 2009; Saitoh et al., 2012, 2015b, 2015a)

			late reduced diffusion		
V983F	DIIS6	Missense	DS	NR	(Martin et al., 2010)
V983A (De novo)	DIIS6	Missense	ICEGTC	Multifocal spikes, high voltage slow-waves (EEG)<br>Reduced current density<br>Shift steady state activation to ore positive values<br>Acelerrated recovery from inactivation	(Fujiwara, 2003; Rhodes et al., 2005)
V983AfsX2 (De novo)	DIIS6	FrameShift	DS	Enlarged extracerebral gap (MRI)	(Wang et al., 2017)
N985I	DIIS6	Missense	DS	Spike waves complex, spike wave in bilateral-frontal (EEG)	(Fujiwara, 2003; Sugawara et al., 2003)
N985S	DIIS6	Missense	DS	NR	(Cetica et al., 2017)
L986F (De novo)	DIIS6	Missense	DS	Gerenalized tonic-clonic seizures<br>Moderate mental retardation<br>Non-fuctional channel	(Claes et al., 2001; Lossin et al., 2003)
L988WfsX5	DIIS6	FrameShift	DS	NR	(Ishii et al., 2017)

F987L (De novo)	DIIS6	Missense	DS	NR	(Wang et al., 2012)
A989V	DIIS6	Missense	Epilepsy	NR	(Lindy et al., 2018)
L990F	DIIS6	Missense	DS	NR	(Hattori et al., 2008)
L991VfsX2 (De novo)	DIIS6	FrameShift	DS	Febrile, partial, generalized tonic-clonic<br>Myoclonic seizures<br>Moderate intellectual disability	(Kwong et al., 2012)
S993R	DII-DIII	Missense	SMEB	NR	(Zuberi et al., 2011)
S994IfsX2	DII-DIII	FrameShift	DS	NR	(Xu et al., 2015)
S994fsX16	DII-DIII	FrameShift	SME	NR	(Xu et al., 2014)
F995L	DII-DIII	Missense	DS	NR	(Xu et al., 2014; Ishii et al., 2017; Lindy et al., 2018)
D998G	DII-DIII	Missense	DS	NR	(Depienne et al., 2008)

D998E	DII-DIII	Missense	Epilepsy	NR	(Lindy et al., 2018)
D998EfsX12	DII-DIII	FrameShift	Epilepsy	NR	(Lindy et al., 2018)
N999_Leu1000del	DII-DIII	In-frame Deletion	DS	NR	(Zuberi et al., 2011)
A1002fsX8 (De novo)	DII-DIII	FrameShift	SMEB	NR	(Ohmori et al., 2002)
I1003fsX	DII-DIII	FrameShift	DS	NR	(Esterhuizen et al., 2018)
D1006fsX4	DII-DIII	FrameShift	SME	NR	(Xu et al., 2014)
E1008X (De novo)	DII-DIII	Nonsense	SMEB	NR	(Harkin et al., 2007; Rodda et al., 2012)
N1011I (De novo)	DII-DIII	Missense	ICEGTC	Rare sharp waves in lateral-temporal (EEG)<br>Moderate mental retardation<br>Reduced current density<br>Shift steady state inactivation to more negative values	(Fujiwara, 2003; Rhodes et al., 2005)

L1012delins 9 (De novo)	DII-DIII	In-frame Deletion	DS	NR	(Wu et al., 2015)
H1020TfsX 4	DII-DIII	FrameShift	Intrantable epilepsy	NR	(Wang et al., 2012)
E1021X (De novo)	DII-DIII	Nonsense	DS	NR	(Nicita et al., 2010)
K1021fsX1 0	DII-DIII	FrameShift	DS	NR	(Xu et al., 2014)
Y1025X	DII-DIII	Nonsense	Intrantable epilepsy	NR	(Wang et al., 2012)
K1027X (De novo)	DII-DIII	Nonsense	DS	NR	(Ohmori et al., 2002)
E1032X	DII-DIII	Nonsense	DS	NR	(Lin et al., 2010; Rocca et al., 2010)
E1032DfsX 14 (De novo)	DII-DIII	FrameShift	DS	NR	(Harkin et al., 2007)
Q1035X	DII-DIII	Nonsense	DS	NR	(Ishii et al., 2017)
Q1035HfsX 11	DII-DIII	FrameShift	DS	NR	(Ishii et al., 2017)

Q1036X	DII-DIII	Nonsense	SME	NR	(Xu et al., 2014)
Q1042X	DII-DIII	Nonsense	Intrantable epilepsy	NR	(Wang et al., 2012)
D1046MfsX9 (De novo)	DII-DIII	FrameShift	DS	Diffuse cerebral edema (Computed tomography)	(Myers et al., 2017b)
E1047X (De novo)	DII-DIII	Nonsense	DS	NR	(Zuberi et al., 2011)
N1056D (De novo)	DII-DIII	Missense	DS	NR	(Lim et al., 2011; Xu et al., 2014)
K1058TfsX21 (De novo)	DII-DIII	FrameShift	DS	NR	(Riva et al., 2009)
D1059Y	DII-DIII	Missense	DS	NR	(Zhou et al., 2018)
C1061X (De novo)	DII-DIII	Nonsense	DS	NR	(Depienne et al., 2008)
N1064IfsX14	DII-DIII	FrameShift	DS	NR	(Orrico et al., 2009)

T1065SfsX 8 (De novo)	DII-DIII	FrameShift	DS	NR	(Nabbout et al., 2003)
T1066A	DII-DIII	Missense	DS	NR	(Ishii et al., 2017)
E1068K	DII-DIII	Missense	DS	NR	(Depienne et al., 2008)
D1078Mfs X2 (De novo)	DII-DIII	FrameShift	SMEB	NR	(Harkin et al., 2007)
G1081R	DII-DIII	Missense	Juvenile abscente epilepsy	NR	(Escayg et al., 2001)
S1084C (Paternal)	DII-DIII	Missense	Juvenile myoclonic epilepsy DS	Paroxysmal generalised polyspike-and-wavecomplexeswithmy oclonic seizures (EEG)	(Jingami et al., 2014)
G1087E	DII-DIII	Missense	GEFS+	NR	(Cetica et al., 2017)
S1090KfsX 4	DII-DIII	FrameShift	Epilepsy	NR	(Lindy et al., 2018)
K1094NfsX 13	DII-DIII	FrameShift	DS	NR	(Cetica et al., 2017)

E1099X (De novo)	DII-DIII	Missense	DS	Isolated diffuse spikes-waves; prominent generalized polyspike-wave paroxysms and multifocal spikes involving the frontal or temporo-parietal regions (EEG)	(Depienne et al., 2008; Riva et al., 2009)
S1100KfsX8 (De novo)	DII-DIII	FrameShift	DS	Generalized clonic seizures<br>Severe mental retardation	(Claes et al., 2001)
Y1102X (De novo)	DII-DIII	Nonsense	DS	NR	(Wang et al., 2012)
M1103VfsX20	DII-DIII	FrameShift	DS	NR	(Wang et al., 2012)
S1104X (De novo)	DII-DIII	Missense	DS	Febrile seizures	(Depienne et al., 2008; Hernández Chávez et al., 2014)
L1111YfsX12	DII-DIII	FrameShift	DS	NR	(Ishii et al., 2017)
P1116QfsX4 (De novo)	DII-DIII	FrameShift	DS	NR	(Marini et al., 2007; Xu et al., 2014)
E1121X	DII-DIII	Nonsense	Epilepsy	NR	(Lindy et al., 2018)
F1124LfsX4	DII-DIII	FrameShift	DS	NR	(Zhang et al., 2015)

E1125X (De novo)	DII-DIII	Missense	DS	NR	(Wang et al., 2012)
N1128KfsX18 (De novo)	DII-DIII	FrameShift	SMEB	NR	(Lim et al., 2011)
E1130KfsX16	DII-DIII	FrameShift	DS	NR	(Ishii et al., 2017)
D1137IfsX9	DII-DIII	FrameShift	Epilepsy	NR	(Lindy et al., 2018)
K1142X	DII-DIII	Nonsense	DS	NR	(Cetica et al., 2017)
L1145QfsX2	DII-DIII	FrameShift	DS	NR	(Yang et al., 2017)
N1146KfsX17 (De novo)	DII-DIII	FrameShift	DS	NR	(Wang et al., 2012)
E1147X (De novo)	DII-DIII	Nonsense	SME	NR	(Xu et al., 2014; Djémié et al., 2016)
E1147CfsX4	DII-DIII	FrameShift	DS	NR	(Møller et al., 2016)
S1150LfsX5	DII-DIII	FrameShift	DS	NR	(Zuberi et al., 2011)

S1151X (De novo)	DII-DIII	Nonsense	SME<br>DS	Neurologic sequealar with severe regressions of all skills<br>MRI reveals focal hyperintensity in the left frontal lobe	(Depienne et al., 2008; Gaily et al., 2013)
S1152X	DII-DIII	Nonsense	DS	NR	(Xu et al., 2015)
E1153X (De novo)	DII-DIII	Nonsense	DS	Focal epilepsy with frontal-lateral activity (EEG)	(Hernández Chávez et al., 2014)
S1155AfsX9 (De novo)	DII-DIII	FrameShift	DS	NR	(Harkin et al., 2007)
S1155X (De novo)	DII-DIII	Nonsense	DS	NR	(Sun et al., 2010)
I1159fsX5	DII-DIII	FrameShift	DS	NR	(Xu et al., 2014)
P1162KfsX5	DII-DIII	FrameShift	DS	NR	(Zuberi et al., 2011)
P1162LfsX2	DII-DIII	FrameShift	SME	NR	(Wang et al., 2012)
V1163X	DII-DIII	Nonsense	DS	NR	(Sun et al., 2010)

Q1166X	DII-DIII	Nonsense	DS	NR	(Ishii et al., 2017)
Q1166RfsX 3	DII-DIII	FrameShift	DS	NR	(Ishii et al., 2017)
Q1172A	DII-DIII	Missense	DS	NR	(Cho et al., 2018)
T1174S (Maternal)	DII-DIII	Missense	FHM<br>FS	Shify steady state activation to more positive values<br>Delayed fast inactivation<br>Enhanced persistent current	(Escayg et al., 2001; Gargus and Tournay, 2007; Yordanova et al., 2011; Rilstone et al., 2012; Cestèle et al., 2013; Lal et al., 2016)
L1175RfsX 8 (De novo)	DII-DIII	FrameShift	DS	NR	(Fukuma et al., 2004; Wang et al., 2012)
E1176NfsX 32 (De novo)	DII-DIII	FrameShift	DS	Severe intellectual disability<br>Intractable seizures despite multiple anti-epileptic drugs<br>High hypermetropia and a substantial astigmatism	(Willemsen et al., 2012)
C1180SfsX 27	DII-DIII	FrameShift	DS	NR	(Zuberi et al., 2011)
E1183KfsX 25 (De novo)	DII-DIII	FrameShift	DS	NR	(Yang et al., 2017)

C1185VfsX23	DII-DIII	FrameShift	Epilepsy	NR	(Lindy et al., 2018)
C1185X (De novo)	DII-DIII	Nonsense	DS	NR	(Zuberi et al., 2011)
Q1187fsX29 (De novo)	DII-DIII	FrameShift	DS	NR	(Harkin et al., 2007)
K1190SfsX18	DII-DIII	FrameShift	DS	NR	(Cetica et al., 2017)
C1192TfsX29	DII-DIII	FrameShift	DS	NR	(Ishii et al., 2017)
I1194SfsX14 (De novo)	DII-DIII	FrameShift	EE	NR	(Veeramah et al., 2013)
I1194CfsX21	DII-DIII	FrameShift	DS	NR	(Cho et al., 2018)
G1201X	DII-DIII	Nonsense	SMEB	NR	(Zuberi et al., 2011)
Q1203X	DII-DIII	Nonsense	Epilepsy	NR	(Lindy et al., 2018)

Gln1203His fsX5	DII-DIII	FrameShift	DS	NR	(McArdle et al., 2008)
W1204R (Paternal)	DII-DIII	Missense	GEFS+	Enhanced persistent current<br>Shift steady state activation and inactivation to more negative vakues	(Escayg et al., 2001; Lossin et al., 2003; Spampanato et al., 2003; Marini et al., 2007)
W1204S (Maternal)	DII-DIII	Missense	GEFS+	NR	(Zuberi et al., 2011)
W1204X	DII-DIII	Nonsense	DS	NR	(Sugawara et al., 2002)
W1205X	DII-DIII	Nonsense	SME	NR	(Xu et al., 2014)
L1207P	DII-DIII	Missense	DS	Hemiclonic seizures<br>Absence seizures	(Zucca et al., 2008)
R1208K (Maternal)	DII-DIII	FrameShift	SMEB	NR	(Zuberi et al., 2011)
T1210K (De novo)	DII-DIII	Missense	DS	NR	(Sun et al., 2010; Xu et al., 2014)
T1210M (Paternal)	DII-DIII	Missense	GEFS+	NR	(Cetica et al., 2017)

C1211X	DII-DIII	Nonsense	DS	NR	(Cho et al., 2018)
R1213X (De novo)	DII-DIII	Nonsense	SME<br>DS<br>LGS	Rare spikes, multifocal spikes and spike-wave complex (EEG)<br>Severe mental delay	(Fujiwara, 2003; Depienne et al., 2008; Zuberi et al., 2011; Wang et al., 2012; Allen et al., 2013; Xu et al., 2014)
I1214R (De novo)	DIIIS1	Missense	DS	Febrile Seizures, Partial and gerenalized tonic-clonic seizures<br>Absence seizure<br>Mild Intellectual disability	(Kwong et al., 2012)
V1215X	DIIIS1	Nonsense	DS	NR	(Nabbout et al., 2003)
R1216K	DIIIS1	Missense	DS	NR	(Ishii et al., 2017)
E1216X (De novo)	DIIIS1	Nonsense	DS	NR	(Yang et al., 2017)
W1219IfsX 42 (De novo) (De novo)	DIIIS1	FrameShift	SME	Neurologic sequel<br>Left-side hemiparesis	(Gaily et al., 2013)
W1219X	DIIIS1	Nonsense	DS	NR	(Depienne et al., 2008)
E1221K	DIIIS1	Missense	DS	NR	(Zuberi et al., 2011)

E1221Q	DIIIS1	Missense	DS	NR	(Xu et al., 2015)
I1228S	DIIIS1	Missense	Epilepsy	NR	(Lindy et al., 2018)
L1230F (Maternal)	DIIIS1	Missense	GEFS+<br>DS	NR	(Zuberi et al., 2011)
L1230P (De novo)	DIIIS1	Missense	DS	Focal spike-wave complex (EEG)<br>Febrile seizures<br>Myoclonic seizures	(Liu et al., 2018)
S1231T (De novo)	DIIIS1	Missense	DS	NR	(Kearney et al., 2006)
S1231R (De novo)	DIIIS1	Missense	DS	polyspike-wave and multiple spikes (EEG)<br>Moderate mental delay	(Fujiwara, 2003)
G1233R	DIIIS1	Missense	DS	NR	(Nabbout et al., 2003)
L1235fs (De novo)	DIIIS1	FrameShift	DS	NR	(Riva et al., 2009)
A1234VfsX 36	DIIIS1	FrameShift	DS	NR	(Cetica et al., 2017)

A1236P	DIIIS1	Missense	SME	NR	(Xu et al., 2014)
E1238D	DIIIS1	Missense	DS	NR	(Harkin et al., 2007)
D1239Y (De novo)	DIIIS1-S2	Missense	DS	NR	(Depienne et al., 2008)
D1239G (De novo)	DIIIS1-S2	Missense	DS	NR	(Depienne et al., 2008)
I1240YfsX30	DIIIS1-S2	FrameShift	SME	NR	(Wang et al., 2012)
Y1241Rfsx30 (De novo)	DIIIS1-S2	FrameShift	DS	NR	(Sun et al., 2010)
Y1241H	DIIIS1-S2	Missense	DS	NR	(Ishii et al., 2017)
D1243LfsX28 (De novo)	DIIIS1-S2	FrameShift	DS	NR	(Marini et al., 2007; Wang et al., 2012)
Q1244X	DIIIS1-S2	Nonsense	SME<br>DS	NR	(Xu et al., 2014; Wei et al., 2018)

R1245X (De novo)	DIIIS1-S2	Nonsense	SME<br>DS	NR	(Nabbout et al., 2003; Harkin et al., 2007; Depienne et al., 2008; Arlier et al., 2010; Zuberi et al., 2011; Moehring et al., 2013; Xu et al., 2014)
R1245Q (De novo)	DIIIS1-S2	Missense	DS	NR	(Riva et al., 2009; Craig et al., 2012; Cetica et al., 2017)
T1247KfsX 4 (De novo)	DIIIS1-S2	FrameShift	DS	NR	(Depienne et al., 2008)
K1249N (Familial)	DIIIS1-S2	Missense	GEFS+	NR	(Orrico et al., 2009)
T1250M (Familial)	DIIIS1-S2	Missense	SME<br>GEFS+	Generalized tonic-clonic seizures<br>Developmental delay after seizure onset<br>Behavioural problems	(Orrico et al., 2009; Catarino et al., 2011; Lal et al., 2016)
E1253X	DIIIS2	Nonsense	SME	NR	(Xu et al., 2014)
Y1254Mfs X16	DIIIS2	FrameShift	SME	NR	(Cetica et al., 2017)
Y1254C	DIIIS2	Missense	Epilepsy	NR	(Wang et al., 2012)
A1255D (De novo)	DIIIS2	Missense	DS	NR	(Depienne et al., 2008)

V1257fsX13 (De novo)	DIIIS2	FrameShift	DS	NR	(Zucca et al., 2008)
T1260HfsX34	DIIIS2	FrameShift	Epilepsy	NR	(Lindy et al., 2018)
F1259S	DIIIS2	Missense	Epilepsy	NR	(Lindy et al., 2018)
T1260P (De novo)	DIIIS2	Missense	DS	NR	(Sun et al., 2010)
Y1261X (De novo)	DIIIS2	Nonsense	DS	NR	(Depienne et al., 2008)
F1263L (De novo)	DIIIS2	Missense	SMEB	Rare spike-wave complex and poly spike-waves complex (EEG)	(Fujiwara, 2003)
L1265P (De novo)	DIIIS2	Missense	DS	NR	(Ohmori et al., 2002)
E1266A	DIIIS2	Missense	DS	NR	(Zuberi et al., 2011)
L1268GfsX22	DIIIS2	FrameShift	DS	NR	(Xu et al., 2015)

K1270T (Maternal)	DIIIS2	Missense	PEFS+<br>GEFS+	Multiple febrile seizures<br>Generalized tonic clonic seizures<br>Partial becoming generalized tonic-clonic	(Abou-Khalil et al., 2001; Sun et al., 2012; Allen et al., 2017)
K1270NfsX21 (De novo)	DIIIS2	FrameShift	DS	NR	(Zucca et al., 2008)
W1271X	DIIIS2	Nonsense	SMEB	NR	(Ohmori et al., 2002)
A1273V	DIIIS2	Missense	DS	Shift steady state activation and inactivation to more positive values	(Xu et al., 2015; Peters et al., 2016)
Y1274N (Paternal)	DIIIS2	Missense	GEFS+	NR	(Ebrahimi et al., 2010; Tonekaboni et al., 2013)
Tyr1274Ser	DIIIS2	Missense	DS	NR	(Xu et al., 2015)
G1275A (De novo)	DIIIS2	Missense	EE	Prolonged febrile<br>Generalized tonic-clonic seizures<br>Generalized spike-wave complex (EEG)	(Veeramah et al., 2013)
Y1276X	DIIIS2	Nonsense	SME	NR	(Xu et al., 2014)
Q1277X	DIIIS2-S3	Nonsense	SME	NR	(Hattori et al., 2008)

W1284R	DIIIS3	Missense	Epilepsy	NR	(Lindy et al., 2018)
W1284S (De novo)	DIIIS3-S4	Missense	DS	NR	(Depienne et al., 2008)
W1284X	DIIIS3-S4	Nonsense	Development delay with seizures	NR	(Trump et al., 2016)
W1284CfsX7 (De novo)	DIIIS3-S4	FrameShift	DS	Epileptic discharges (EEG)	(Petrelli et al., 2012)
C1285LfsX9	DIIIS3-S4	FrameShift	DS	NR	(Lindy et al., 2018)
W1286X (De novo)	DIIIS3-S4	Nonsense	DS	NR	(Sun et al., 2010)
L1287Pro (De novo)	DIIIS3-S4	Missense	DS	NR	(Sun et al., 2010)
D1288N (De novo)	DIIIS3-S4	Missense	DS	NR	(Zuberi et al., 2011)
D1288G	DIIIS3-S4	Missense	Epilepsy	NR	(Lindy et al., 2018)

F1289del (De novo)	DIIIS3-S4	In-frame Deletion	SMEB DS	Non-functional channel	(Ohmori et al., 2002, 2006; Margherita Mancardi et al., 2006; Depienne et al., 2008)
L1290X	DIIIS3-S4	Nonsense	DS	NR	(Cetica et al., 2017)
D1293X	DIIIS3-S4	Nonsense	DS	NR	(Cetica et al., 2017)
D1293VfsX 7 (Familial)	DIIIS3-S4	FrameShift	DS	NR	(Marini et al., 2007; Depienne et al., 2008, 2010)
T1300R	DIIIS3-S4	Missense	DS	NR	(Xu et al., 2014)
T1300KfsX 19	DIIIS3-S4	FrameShift	DS	NR	(Ishii et al., 2017)
C1303MfsX 29 (De novo)	DIIIS3-S4	FrameShift	LGS	NR	(Allen et al., 2013)
Y1306X	DIIIS3-S4	Nonsense	Epilepsy	NR	(Lindy et al., 2018)
E1308D(Familial)	DIIIS3-S4	Missense	DS	NR	(Orrico et al., 2009; Nicita et al., 2010; Zuberi et al., 2011; Lal et al., 2016; Maksemous et al., 2016)
L1309F (Paternal)	DIIIS4	Missense	GEFS+	Generalized clonic seizures of changing lateralization	(Dimova et al., 2010; Allen et al., 2017)

				Myoclonic, atonic seizures<br>Generalized polyspike-wave discharges EEG)	
L1309SfsX 10	DIIIS4	FrameShift	DS	NR	(Ishii et al., 2017)
G1310EfsX 9	DIIIS4	FrameShift	DS	NR	(Zuberi et al., 2011)
A1311PfsX 8 (De novo)	DIIIS4	FrameShift	DS	NR	(Zuberi et al., 2011)
K1313I	DIIIS4	Missense	SME	NR	(Lee et al., 2014)
R1316W (De novo)	DIIIS4	Missense	SMEB	NR	(Lim et al., 2011)
R1316G	DIIIS4	Missense	DS	NR	(Wang et al., 2012)
R1316S<br>(De novo)	DIIIS4	Missense	DS	NR	(Depienne et al., 2008; Kothur et al., 2018)
L1318R (De novo)	DIIIS4	Missense	DS	Bifrontal sharp waves in right side (EEG)	(Mak et al., 2011)

R1319X	DIIIS4	Nonsense	DS	NR	(Cetica et al., 2017)
R1319SfsX 12 (De novo)	DIIIS4	FrameShift	DS	NR	(Depienne et al., 2008)
A1320V (De novo)	DIIIS4	mISSENSE	DS	NR	(Zuberi et al., 2011)
R1322Il	DIIIS4	Missense	SME	NR	(Lee et al., 2014)
R1322SfsX 6	DIIIS4	FrameShift	Epilepsy	NR	(Lindy et al., 2018)
P1323LfsX 2	DIIIS4	FrameShift	DS	NR	(Zuberi et al., 2011)
P1323R(De novo)	DIIIS4	Missense	PEFS+	NR	(Lim et al., 2011)
L1324fsX1	DIIIS4	FrameShift	SME	NR	(Xu et al., 2014)
L1324fsX8 (De novo)	DIIIS4	FrameShift	DS<br>SME	NR	(Rocca et al., 2010)
R1325T (De novo)	DIIIS4	Missense	Intractable epilepsy	NR	(Wang et al., 2012)

A1326P (De novo)	DIIIS4	Missense	SME<br>DS	NR	(Wallace et al., 2003; Zuberi et al., 2011; Rodda et al., 2012)
A1326D(De novo)	DIIIS4	Missense	SMEB	NR	(Singh et al., 2009)
A1326V (De novo)	DIIIS4	Missense	Epilepsy-aphasia	NR	(Carvill et al., 2013)
S1328P	DIIIS4	Missense	DS	NR	(Depienne et al., 2008; Wang et al., 2012; Xiong et al., 2015)
R1329X (De novo; Maternal)	DIIIS4	Nonsense	DS	Myoclonic seizures<br>Generalized tonic-clonic seizures	(Depienne et al., 2008; Selmer et al., 2009; Zuberi et al., 2011; Lindy et al., 2018)
F1330LfsX3	DIIIS4	FrameShift	DS	Generalized tonic-clonic seizures<br>Status epilepticus and absence	(Lemke et al., 2012)
G1332E	DIIIS4	Missense	DS	NR	(Ishii et al., 2017)
M1333T	DIIIS4	Missense	GEFS+	NR	(Cantarín Extremera et al., 2011)
R1334M	DIIIS4	Missense	Epilepsy	NR	(Coll et al., 2017)

V1335M (De novo)	DIIIS4-S5	Missense	DS	Generalized tonic-clonic seizures<br>Absence seizures<br>Multifocal and diffuse spike-waves (EEG)	(Sun et al., 2008a; Zucca et al., 2008)
N1338fsX1338 (De novo)	DIIIS4-S5	FrameShift	DS	NR	(Lindy et al., 2018)
A1339V (De novo)	DIIIS4-S5	Missense	SME<br>DS	NR	(Okumura et al., 2012; Wang et al., 2012; Xu et al., 2014)
A1339D (De novo)	DIIIS4-S5	Missense	Epilepsy	NR	(Lindy et al., 2018)
V1339G (De novo)	DIIIS4-S5	Missense	DS	NR	(Esterhuizen et al., 2018)
L1340FfsX38	DIIIS4-S5	FrameShift	Epilepsy	NR	(Lindy et al., 2018)
L1341fsX8	DIIIS4-S5	FrameShift	SME	Febrile generalized clonic seizure	(Sanchez-Carpintero et al., 2013)
L1341WfsX37	DIIIS4-S5	FrameShift	DS	NR	(Depienne et al., 2008)
A1343P	DIIIS4-S5	Missense	Epilepsy	NR	(Lindy et al., 2018)

I1344FfsX5	DIIIS4-S5	FrameShift	Epilepsy	NR	(Lindy et al., 2018)
I1344M	DIIIS4-S5	Missense	DS	NR	(Wang et al., 2012)
P1345S (De novo)	DIIIS4-S5	Missense	EE	NR	(Carvill et al., 2013)
P1345L (De novo)	DIIIS4-S5	Missense	Malignant migrating partial seizures of infancy	NR	(Trump et al., 2016)
I1347T (De novo)	DIIIS4-S5	Missense	EE	NR	(de Kovel et al., 2016)
M1348I	DIIIS4-S5	Missense	DS	NR	(Xu et al., 2015)
V1350G (De novo)	DIIIS4-S5	Missense	DS	NR	(Zuberi et al., 2011)
V1350AfsX28 (De novo)	DIIIS4-S5	FrameShift	DS	NR	(Wang et al., 2012)
L1352P	DIIIS5	Missense	DS	NR	(Xu et al., 2015)

V1353L (Maternal)	DIIIS5	Missense	PEFS+ GEFS+	Non-functional channel	(Wallace et al., 2001; Lossin et al., 2003; Bennett et al., 2017)
C1354R	DIIIS5	Missense	Epilepsy	NR	(Lindy et al., 2018)
C1354Y	DIIIS5	Missense	SME	Slow background, generalized spikes (EEG)	(Lee et al., 2014)
I1356TfsX4 (De novo)	DIIIS5	FrameShift	SMEB	NR	(Harkin et al., 2007)
L1355P	DIIIS5	Missense	SMEB	NR	(Wang et al., 2012)
F1357L	DIIIS5	Missense	Intractable epilepsy	NR	(Wang et al., 2012)
W1358R(De novo)	DIIIS5	Missense	DS	NR	(Zuberi et al., 2011)
W1358X (De novo)	DIIIS5	Nonsense	DS	NR	(Zuberi et al., 2011)
W1358S (De novo)	DIIIS5	Missense	DS	Hemiclonic seizures<br>Severe mental retardation	(Zucca et al., 2008; Petrelli et al., 2012)
L1359I	DIIIS5	Missense	DS	NR	(Cetica et al., 2017)

L1359Q (De novo)	DIIIS5	Missense	DS	NR	(Gokben et al., 2017)
S1362R	DIIIS5	Missense	SME	NR	(Xu et al., 2014)
I1363N	DIIIS5	Missense	SME	NR	(Xu et al., 2014)
I1363fsX4	DIIIS5	FrameShift	SMEB	NR	(Wang et al., 2012)
M1364Lys	DIIIS5	Missense	SME	NR	(Villeneuve et al., 2014)
V1366I	DIIIS5	Missense	ICEGTC GEEFS+ FS+	4-5Hz diffuse irregular spikes and wave complex at a drowsy state; Diffuse bilateral spike-waves only when valproate was reduced, otherwise no paroxysmal discharges were recorded (EEG)	(Osaka et al., 2007)
V1366L	DIIIS5	Missense	Epilepsy	NR	(Lindy et al., 2018)
N1367K	DIIIS5	Missense	SME	NR	(Depienne et al., 2008)
F1369LfsX 21	DIIIS5	FrameShift	Epilepsy	NR	(Lindy et al., 2018)

A1370P	DIIIS5	Missense	SMEB	NR	(Lindy et al., 2018)
A1370V	DIIIS5	Missense	DS<br>GEFS+	NR	(Cetica et al., 2017)
G1371V (De novo)	DIIIS5	Missense	SME<br>DS	NR	(Dhamija et al., 2014; Liu et al., 2018)
K1372E	DIIIS5	Missense	GEFS+	Tonic-clonic and myoclonic seizures without fever<br>Slow spike waves from the left fronto-central region (EEG)	(Goldberg-Stern et al., 2014)
F1373S	DIIIS5	Missense	DS	NR	(Cetica et al., 2017)
C1376R (De novo)	DIIIS5	Missense	DS	NR	(Wang et al., 2012)
C1376YfsX2 (De novo)	DIIIS5	FrameShift	DS	NR	(Lim et al., 2011; Kim et al., 2013)
N1378H	DIIIS5-S6	Missense	DS	NR	(Zuberi et al., 2011)
N1378TfsX13	DIIIS5-S6	FrameShift	DS	NR	(Zuberi et al., 2011; Cho et al., 2018)

V1379L	DIIIS5-S6	Missense	PEFS+	Febrile and generalized seizures<br>Focal seizures with secondary generalization	(Mhanni et al., 2011)
G1382V (De novo)	DIIIS5-S6	Missense	DS	Diffuse spike-waves domintant in bilatareal frontal regions (EEG)	(Haginoya et al., 2018; Liu et al., 2018)
D1383GfsX4	DIIIS5-S6	FrameShift	DS	Complex partial seizures with generalization<br>Tonic seizures<br>Myoclonic astatic seizures	(Surovy et al., 2016)
F1385V	DIIIS5-S6	Missense	DS	NR	(Wang et al., 2012)
D1389AfsX2	DIIIS5-S6	FrameShift	Epilepsy	NR	(Lindy et al., 2018)
V1390M (De novo)	DIIIS5-S6	Missense	SME<br>SMEB<br>DS	Generalized spikes, focal spikes and polyspikes (EEG)	(Ohmori et al., 2002; Harkin et al., 2007; Sun et al., 2008a; Rilstone et al., 2012; Verbeek et al., 2013; Lee et al., 2014; Cho et al., 2018)
N1391S	DIIIS5-S6	Missense	Epilepsy	NR	(Depienne et al., 2008)
N1392K (De novo)	DIIIS5-S6	Missense	EEIE	NR	(Arafat et al., 2017)
His1393Asp (De novo)	DIIIS5-S6	Missense	GEFS+	FS+ with focal motor and absence	(Myers et al., 2017a)

H1393P (De novo)	DIIIS5-S6	Missense	SMEB	Febrile generalized tonic- clonic seizures	(Stefanaki et al., 2006)
T1394I (De novo)	DIIIS5-S6	Missense	DS	NR	(Zuberi et al., 2011)
D1396Y(De novo)	DIIIS5-S6	Missense	DS	Prolonged febrile seizures<br>Generalized tonic-clonic seizures<br>Atonic seizures	(Specchio et al., 2010)
C1396G (De novo)	DIIIS5-S6	Missense	SMEB	NR	(Harkin et al., 2007)
C1396Y (De novo)	DIIIS5-S6	Missense	DS	NR	(Zuberi et al., 2011)
C1396X	DIIIS5-S6	Nonsense	SME	NR	(Xu et al., 2014)
L1397X	DIIIS5-S6	Nonsense	DS	NR	(Depienne et al., 2008)
E1401KfsX 3	DIIIS5-S6	FrameShift	DS	NR	(Depienne et al., 2008)
R1402fsX9 (De novo)	DIIIS5-S6	FrameShift	DS	Generalized tonic-clonic seizures<br>Left-sided frontal epileptiform activity with secondary generalization (EEG)	(Yordanova et al., 2011)

A1406T(Parternal)	DIIIS5-S6	Missense	DS	NR	(Lim et al., 2011)
A1406GfsX11	DIIIS5-S6	FrameShift	Epilepsy	NR	(Lindy et al., 2018)
R1407X (De novo)	DIIIS5-S6	Nonsense	DS	Non-functional channel	(Sugawara et al., 2002, 2003; Harkin et al., 2007; Wang et al., 2012)
W1408X (De novo)	DIIIS5-S6	Nonsense	DS	Generalized tonic-clonic seizures<br>Frontal-dominante spike-wave, poly spike-waves (EEG)	(Fujiwara, 2003)
N1410MfsX2 (De novo)	DIIIS5-S6	FrameShift	DS	Febrile Seizures<br>Partial and gerenalized tonic-clonic seizures<br>Absence seizure<br>Mild Intellectual disability	(Kwong et al., 2012)
N1414D (De novo)	DIIIS5-S6	Missense	GEFS+	NR	(Zuberi et al., 2011)
N1414Y(De novo)	DIIIS5-S6	Missense	DS	NR	(Marini et al., 2007)
F1415I	DIIIS5-S6	Missense	DS	Shift steady state inactivation to more positive values	(Jiao et al., 2013)
F1415LfsX4	DIIIS5-S6	FrameShift	DS	NR	(Ishii et al., 2017)

F1415X (De novo)	DIIIS5-S6	Nonsense	DS	NR	(Depienne et al., 2008; Sun et al., 2010)
D1416H (De novo)	DIIIS5-S6	Missense	SME	NR	(Mulley et al., 2013)
D1416G (De novo)	DIIIS5-S6	Missense	DS	NR	(Depienne et al., 2008)
N1417S (De novo)	DIIIS5-S6	Missense	DS	NR	(Zuberi et al., 2011)
V1418G	DIIIS5-S6	Missense	DS	NR	(Wang et al., 2012)
G1419DfsX19 (De novo)	DIIIS5-S6	FrameShift	DS	NR	(Depienne et al., 2008)
G1419X	DIIIS5-S6	Nonsense	Epilepsy	NR	(Lindy et al., 2018)
G1421W	DIIIS5-S6	Missense	DS	NR	(Cho et al., 2018)
G1421AfsX18	DIIIS5-S6	FrameShift	DS	Generalized tonic-clonic seizures<br>Hemiconvulsion	(de Lange et al., 2018)
Y1422C (De novo)	DIIIS5-S6	Missense	DS	NR	(Riva et al., 2009)

Y1422X	DIIIS5-S6	Nonsense	Intractable Epilepsy	NR	(Wang et al., 2012; Zhu et al., 2017)
L1423F (Maternal)	DIIIS5-S6	Missense	DS	NR	(Zuberi et al., 2011)
L1426R (De novo)	DIIIS5-S6	Missense	DS	NR	(Riva et al., 2009)
Q1427cfsX 16	DIIIS5-S6	FrameShift	SME	NR	(Verbeek et al., 2013)
Q1427X (De novo)	DIIIS5-S6	Nonsense	SMEB	NR	(Harkin et al., 2007)
Q1427P (De novo)	DIIIS5-S6	Missense	DS	NR	(Wang et al., 2012)
V1428A	DIIIS5-S6	Missense	GEFS+	Febrile seizures<br>Right-temporal spike-wave (EEG)	(Sugawara et al., 2002; Perucca et al., 2017)
A1429del (De novo)	DIIIS5-S6	In-frame Deletion	DS	NR	(Zuberi et al., 2011)
A1429S (Paternal)	DIIIS5-S6	Missense	Autossomal dominant nocturnal frontal lobe epilepsy	NR	(Sone et al., 2012)

A1429T	DIIIS5-S6	Missense	Epilepsy	NR	(Lindy et al., 2018)
A1429D	DIIIS5-S6	Missense	Intractable epilepsy	NR	(Wang et al., 2012)
A1429DfsX 15	DIIIS5-S6	FrameShift	SMEB	NR	(Ohmori et al., 2002)
A1429V (De novo)	DIIIS5-S6	Missense	GEFS+	MRI reveals a Pineal cyst<br>FS+ with focal motor seizures	(Myers et al., 2017a)
F1431I	DIIIS5-S6	Missense	DS	NR	(Depienne et al., 2008)
K1432R	DIIIS5-S6	Missense	SME	Generalized spikes (Ictal EEG)	(Lee et al., 2014)
K1432KfsX 6 (De novo)	DIIIS5-S6	FrameShift	DS	NR	(Lim et al., 2011)
G1433R (De novo)	DIIIS5-S6	Missense	DS	NR	(Nicita et al., 2010)
G1433V (Paternal)	DIIIS5-S6	Missense	DS	NR	(Zuberi et al., 2011)

G1433E (De novo)	DIIIS5-S6	Missense	DS<br>SME	Rare multifocal spikes (EEG)<br>Hemiclonic seizures	(Sun et al., 2008a; Verbeek et al., 2013; Xu et al., 2014)
W1434R (De novo)	DIIIS5-S6	Missense	DS<br>SME	Myoclonic seizures<br>Severe regression of all skills after seizures<br>Severe psychomotor disability	(Claes et al., 2003; Gaily et al., 2013)
W1434X (De novo)	DIIIS5-S6	Nonsense	DS	Hemiclonic seizures	(Zucca et al., 2008) (Xu et al., 2015)
M1435K	DIIIS5-S6	Missense	Epilepsy	NR	(Lindy et al., 2018)
M1435R	DIIIS5-S6	Missense	Epilepsy	NR	(Lindy et al., 2018)
I1437M (De novo)	DIIIS5-S6	Missense	DS	NR	(Depienne et al., 2008)
M1438 (De novo)	DIIIS5-S6	Missense	DS	NR	(Do et al., 2017)
A1440QfsX 36	DIIIS5-S6	FrameShift	Epilepsy	NR	(Lindy et al., 2018)
A1440E (De novo)	DIIIS5-S6	Missense	SME	NR	(Gaily et al., 2013)

A1440G	DIIIS5-S6	Missense	Juvenile myoclonic epilepsy	NR	(Lee et al., 2018)
A1440V	DIIIS5-S6	Missense	Autism spectrum disorder	Tonic-clonic and potentially myoclonic seizures	(D'Gama et al., 2015)
A1441T	DIIIS5-S6	Missense	SME	Focal spikes; generalized epileptiform discharges (EEG)	(Lee et al., 2014)
A1441P (De novo)	DIIIS5-S6	Missense	DS	NR	(Harkin et al., 2007; Mulley et al., 2013)
Q1450K (De novo)	DIIIS5-S6	Missense	DS	NR	(Zuberi et al., 2011)
Q1450P (De novo)	DIIIS5-S6	Missense	SME	Slow background (EEG)	(Lin et al., 2013; Lee et al., 2014)
Q1450R (De novo)	DIIIS5-S6	Missense	SMEB	NR	(Ohmori et al., 2002)
P1451S (De novo)	DIIIS5-S6	Missense	DS	NR	(Zuberi et al., 2011)
P1451L (De novo)	DIIIS5-S6	Missense	DS	NR	(Riva et al., 2009)
Y1451CfsX22 (De novo)	DIIIS5-S6	FraneShift	DS	Multifocal discharges (EEG)	(Liu et al., 2018)

Y1453C	DIIIS5-S6	Missense	SMEB	NR	(Wang et al., 2012)
Y1453X (De novo)	DIIIS5-S6	Nonsense	DS	NR	(Zuberi et al., 2011)
E1454K(De novo)	DIIIS5-S6	Missense	DS	NR	(Depienne et al., 2008)
Leu1461Ile (Paternal)	DIIIS6	Missense	DS	NR	(Nabbout et al., 2003)
Y1462H (De novo)	DIIIS6	Missense	DS<br>SMEB	NR	(Zuberi et al., 2011; Wang et al., 2012)
Y1462LfsX 24 (De novo)	DIIIS6	FrameShift	Epilepsy	NR	(Staněk et al., 2018)
Y1462C	DIIIS6	Missense	DS	Absence and hemiplegic seizures	(Zucca et al., 2008)
Y1462CfsX 22 (De novo)	DIIIS6	FrameShift	DS	NR	(Depienne et al., 2008)
F1463L (De novo)	DIIIS6	Missense	DS	Afebrile unilateral clonic seizure<br>Generalized tonic-clonic<br>Generalized and focal epileptic discharges (EEG)	(Reyes et al., 2011)

F1463S (De novo)	DIIIS6	Missense	DS	NR	(Nabbout et al., 2003) (Lindy et al., 2018)
I1467L	DIIIS6	Missense	DS	NR	(Cetica et al., 2017)
G1470W (De novo)	DIIIS6	Missense	DS	NR	(Marini et al., 2007)
S1471F	DIIIS6	Missense	DS	NR	(Xu et al., 2015)
F1472S (De novo)	DIIIS6	Missense	SMEB	NR	(Wang et al., 2012)
F1473del (De novo)	DIIIS6	In-frame Deletion	DS	NR	(Depienne et al., 2008)
L1475S (De novo)	DIIIS6	Missense	DS	NR	(Riva et al., 2009)
N1476K (De novo)	DIIIS6	Missense	SMEB	NR	(Zuberi et al., 2011)
F1478C	DIIIS6	Missense	Epilepsy	NR	(Lindy et al., 2018)
G1480C	DIIIS6	Missense	Epilepsy	NR	(Lindy et al., 2018)

G1480V (De novo)	DIIIS6	Missense	Myoclonic astatic epilepsy	NR	(Harkin et al., 2007)
I1483del	DIIIS6	In-frame Deletion	DS	NR	(Depienne et al., 2008)
D1484IfsX16 (De novo)	DIII-DIV	FrameShift	Intractable epilepsy	NR	(Wang et al., 2012)
D1484G	DIII-DIV	Missense	SMEB	NR	(Depienne et al., 2008)
N1485D (De novo)	DIII-DIV	Missense	SME	NR	(Carvill et al., 2013)
N1485Y	DIII-DIV	Missense	DS	NR	(Wang et al., 2012)
Q1489K (Maternal)	DIII-DIV	Missense	FHM	Shift steady state activation to more negative values<br>Faster recovery from inactivation at negative voltages	(Dichgans et al., 2005)
K1491X	DIII-DIV	Nonsense	DS	NR	(Zuberi et al., 2011; Wang et al., 2012)
K1492EfsX18	DIII-DIV	FrameShift	DS	NR	(Ishii et al., 2017)

K1492N(De novo)	DIII-DIV	Missense	DS	NR	(Yang et al., 2017)
G1495Dfs7	DIII-DIV	FrameShift	DS	Afebrile seizures	(Haginoya et al., 2018)
G1495VfsX6 (De novo)	DIII-DIV	FrameShift	DS	NR	(Yang et al., 2017)
Q1496KfsX5 (De novo)	DIII-DIV	FrameShift	DS	NR	(Riva et al., 2009)
I1498N	DIII-DIV	Missense	Epilepsy	NR	(Lindy et al., 2018)
I1498M (Parternal)	DIII-DIV	Missense	FHM	Hemiplegic attacks were usually preceded by flashing lights or scotoma, speech problems and/or unilateral sensory symptoms	(Weller et al., 2014)
F1499LfsX2	DIII-DIV	FrameShift	SME	Generalized tonic–clonic convulsion;<br>Epileptic attack after photic stimulation with associated ictal discharges and temporal spikes waves (EEG)	(Lam et al., 2015)
F1499LfsX2	DIII-DIV	FrameShift	DS	NR	(Zuberi et al., 2011)
T1501fs	DIII-DIV	FrameShift	DS	NR	(Do et al., 2017)

E1503K (De novo)	DIII-DIV	Missense	SMEB	NR	(Shi et al., 2009; Zuberi et al., 2011; Wang et al., 2012)
E1503del (De novo)	DIII-DIV	In-frame Deletion	SMEB	NR	(Wang et al., 2012)
E1503G	DIII-DIV	Missense	SME	Background with tetha activity and focal left-frontal focal discharges (EEG)	(Moehring et al., 2013)
Q1504L	DIII-DIV	Missense	Epilepsy	NR	(Lindy et al., 2018)
K1506NfsX9 (De novo)	DIII-DIV	FrameShift	DS	NR	(Depienne et al., 2008)
Y1507D	DIII-DIV	Missense	DS	NR	(Ishii et al., 2017)
Y1507X	DIII-DIV	Nonsense	DS	NR	(Farwell et al., 2015; Helbig et al., 2016)
Y1508X	DIII-DIV	Nonsense	DS	NR	(Zuberi et al., 2011)
D1509MfsX3 (De novo)	DIII-DIV	FrameShift	DS	NR	(Harkin et al., 2007)

Me511K	DIII-DIV	Missense	Intractable epilepsy	NR	(Wang et al., 2012)
M1511R	DIII-DIV	Missense	Epilepsy	NR	(Lindy et al., 2018)
K1513NfsX2	DIII-DIV	FrameShift	DS	NR	(Wang et al., 2012)
L1514IfsX23 (De novo)	DIII-DIV	FrameShift	DS	NR	(Depienne et al., 2008)
L1514S(Maternal)	DIII-DIV	Missense	DS	NR	(Depienne et al., 2008)
G1515R	DIII-DIV	Missense	Epilepsy	NR	(Lindy et al., 2018)
S1516X (De novo)	DIII-DIV	Nonsense	DS	NR	(Sugawara et al., 2002; Harkin et al., 2007; Depienne et al., 2008; Riva et al., 2009; Zuberi et al., 2011)
S1516W	DIII-DIV	Missense	DS	NR	(Xu et al., 2015)
S1516L	DIII-DIV	Missense	Epilepsy	NR	(Lindy et al., 2018)
K1518NfsX21	DIII-DIV	FrameShift	Epilepsy	NR	(Depienne et al., 2008)

K1517X	DIII-DIV	Nonsense	DS	NR	(Xu et al., 2015)
K1518NfsX 21 (De novo)	DIII-DIV	FrameShift	DS	NR	(Depienne et al., 2008)
P1519TfsX 18	DIII-DIV	FrameShift	DS	NR	(Hesse et al., 2018)
P1519T	DIII-DIV	Missense	SME	Bilateral deactivation in the posterior cingulate cortex associated with the interictal epileptiform discharges together with deactivated areas in both parietal lobes (EEG)	(Moehring et al., 2013; Lindy et al., 2018)
P1519S	DIII-DIV	Missense	Epilepsy	NR	(Hesse et al., 2018)
P1519L	DIII-DIV	Missense	Epilepsy	NR	(Lindy et al., 2018)
Q1520KfsX 19 (De novo)	DIII-DIV	FrameShift	DS	Febrile Seizures<br>Partial Seizures	(Kwong et al., 2012)
K1521SfsX 18	DIII-DIV	FrameShift	Epilepsy	NR	(Hesse et al., 2018)
I1523T	DIII-DIV	Missense	SME	Generalized tonic-clonic seizures<br>Febrile seizures	(Catarino et al., 2011)

				Austistic features<br>Behavioural problems	
R1525X (De novo)	DIII-DIV	Nonsense	DS<br>SMEB<br>Myoclonic astatic epilepsy	Febrile seizures<br>Tonic-clonic seizures	(Kearney et al., 2006; Harkin et al., 2007; Depienne et al., 2008; Orrico et al., 2009; Vadlamudi et al., 2010; Zuberi et al., 2011; Djémié et al., 2016)
R1525Q	DIII-DIV	Missense	DS	Shift steady state activation to more negative values	(Binini et al., 2017; Cetica et al., 2017)
P1526QfsX13	DIII-DIV	FrameShift	DS	NR	(Zuberi et al., 2011)
N1528K	DIII-DIV	Missense	DS	NR	(Ishii et al., 2017)
K1529NfsX9	DIII-DIV	FrameShift	DS	Generalized tonic-clonic seizures<br>Mental retardation;<br>Obsessive-compulsive disorder	(Skjei et al., 2015)
F1535fs	DIII-DIV	FrameShift	SME	NR	(Marini et al., 2007)
V1538I	DIVS1	Missense	SME<br>Acute encephalopathy	NR	(Depienne et al., 2008)
T1539P	DIVS1	Missense	SME	NR	(Verbeek et al., 2013)

F1543S(Maternal)	DIVS1	Missense	Cyptogenic focal epilepsy<br>PEFS+	NR	(Harkin et al., 2007; Kim et al., 2014)
F1543fsX2	DIVS1	FrameShift	DS	NR	(Zuberi et al., 2011)
D1544H	DIVS1	Missense	Epilepsy	NR	(Lindy et al., 2018)
D1544G (De novo)	DIVS1	Missense	DS	NR	(Zuberi et al., 2011)
D1544A (De novo)	DIVS1	Missense	DS	NR	(Depienne et al., 2008)
I1545V (De novo)	DIVS1	Missense	DS	NR	(Harkin et al., 2007; Wang et al., 2012; Cetica et al., 2017)
S1546fsX1548	DIVS1	FrameShift	Intractable epilepsy	NR	(Wang et al., 2012)
M1548K	DIVS1	Missense	DS	NR	(Cetica et al., 2017)
C1552Y	DIVS1	Misesense	DS	NR	(Wu et al., 2015)

L1553HfsX8	DIVS1	FrameShift	DS	NR	(Zuberi et al., 2011)
L1553_N1554delinsHisIle	DIVS1	In-frame Deletion	DS	NR	(Cetica et al., 2017)
N1554KfsX4	DIVS1	FrameShift	DS	NR	(Lim et al., 2011)
M1555R	DIVS1	Missense	DS	NR	(Wang et al., 2012)
M1558del	DIVS1	In-frame Deletion	DS	NR	(Wang et al., 2012)
M1558I	DIVS1	Missense	Epilepsy	NR	(Lindy et al., 2018)
M1559K	DIVS1	Missense	DS	Hemiclonic and generalized tonic clonic seizures	(Kumar et al., 2018)
V1560EfsX5	DIVS1	FrameShift	DS	NR	(Wang et al., 2012)
E1561K	DIVS2	Missense	DS	NR	(Depienne et al., 2008)
E1561X	DIVS2	Nonsense	Epilepsy	NR	(Lindy et al., 2018)

Q1565X	DIVS2	Nonsense	Epilepsy	NR	(Lindy et al., 2018)
T1571TfsX 20	DIVS2	FrameShift	SMEB	NR	(Wang et al., 2012)
S1574HfsX 17	DIVS2	FrameShift	DS	NR	(Depienne et al., 2008)
R1575C (De novo; Maternal; Paternal)	DIVS2	Missense	DS	Focal seizures<br>Mental deterioration<br>Enhanced persistent current	(Depienne et al., 2008; Ohmori et al., 2008; Lim et al., 2011; Saitoh et al., 2012, 2015a)
I1576T	DIVS2	Missense	DS	NR	(Tuncer et al., 2015)
V1579E	DIVS2	Missense	DS	NR	(Depienne et al., 2008)
H1583R	DIVS2	Missense	GEFS+	Generalized tonic-clonic seizures	(Hattori et al., 2008) (Tan et al., 2012)
T1585fsX6	DIVS2	FrameShift	DS	NR	(Wang et al., 2012)
G1586E (De novo)	DIVS2	Missense	DS	NR	(Depienne et al., 2008; Sun et al., 2010; Zuberi et al., 2011)

C1588R (De novo)	DIVS2	Missense	DS	NR	(Marini et al., 2007; Zuberi et al., 2011; Mercimek-Mahmutoglu et al., 2015)
C1588LfsX 21 (De novo)	DIVS2	FrameShift	DS	NR	(Orrico et al., 2009)
C1588X	DIVS2	Nonsense	DS	NR	(Cetica et al., 2017)
Val1589Gly	DIVS2	Missense	DS	NR	(Xu et al., 2015)
Leu1590Pro	DIVS2	Missense	EE	NR	(Ostrander et al., 2018)
Lys1591X	DIVS2	Nonsense	DS	NR	(Wang et al., 2012)
Leu1592His	DIVS2	Missense	SMEB	NR	(Zuberi et al., 2011)
Leu1592Pro	DIVS2	Missense	DS	NR	(Zuberi et al., 2011)
Ile1593Leuf sX15	DIVS2	FrameShift	Epilepsy	NR	(Lindy et al., 2018)
Ile1593Asn	DIVS2	Missense	DS	NR	(Cho et al., 2018)

Ser1594Tyr fsX14	DIVS2	FrameShift	DS	NR	(Wang et al., 2012)
Leu1595Thr fsX13	DIVS2	FrameShift	DS	NR	(Wang et al., 2012)
Arg1596Cys (De novo; Parternal)	DIVS2-S3	Missense	FS+<br>DS	Myoclonic seizures<br>Generalized tonic-clonic seizures	(Dlugos et al., 2007; Hoffman-Zacharska et al., 2015)
Arg1596His (Paternal)	DIVS2-S3	Missense	GEFS+	NR	(Hoffman-Zacharska et al., 2015)
R1596L	DIVS2-S3	Missense	DS	NR	(Depienne et al., 2008)
Y1598F	DIVS2-S3	Missense	GEFS+	NR	(Lindy et al., 2018)
Y1598X	DIVS2-S3	Nonsense	DS	NR	(Harkin et al., 2007)
I1602LfsX16 (De novo)	DIVS2-S3	FrameShift	DS	NR	(Yang et al., 2017)
W1604X	DIVS3	Nonsense	DS	NR	(Xu et al., 2015)

N1605S (De novo)	DIVS3	Missense	SMEB	NR	(Zuberi et al., 2011)
N1605I	DIVS3	Missense	DS	NR	(Parrini et al., 2017)
F1607X	DIVS3	Nonsense	DS	NR	(Harkin et al., 2007; Parrini et al., 2017)
F1607LfsX 11	DIVS3	FrameShift	Epilepsy	NR	(Lindy et al., 2018)
D1608Y (De novo)	DIVS3	Missense	DS	NR	(Marini et al., 2007; Xu et al., 2014)
D1608VfsX 10 (De novo)	DIVS3	FrameShift	DS	NR	(Sun et al., 2010)
D1608G (De novo)	DIVS3	Missense	DS	NR	(Wang et al., 2012)
F1609LfsX 1610 (De novo)	DIVS3	FrameShift	DS	NR	(Depienne et al., 2008)
V1610fsX3 3	DIVS3	FrameShift	DS	NR	(Xu et al., 2014)
l1611WfsX 8	DIVS3	FrameShift	DS	NR	(Xu et al., 2015)

V1611F (Maternal)	DIVS3	Missense	GEFS+<br>ICEGTC	Generalized tonic-clonic seizure<br><br>Enhanced persistent current<br>Shift steady state activation and inactivation to more negative values	(Fujiwara, 2003; Rhodes et al., 2005)
V1612I (Partenal; Maternal)	DIVS3	Missense	DS	Febrile Seizures<br>Partial and gerenalized tonic-clonic seizures<br>Absence seizure<br>Moderate Intellectual disability<br>Focal sharp waves; spike-wave discharges (EEG)	(Depienne et al., 2008; Kwong et al., 2012; Lee et al., 2014)
L1614PfsX 4 (De novo)	DIVS3	FrameShift	DS	NR	(Zuberi et al., 2011)
S1615fs	DIVS3	FrameShift	SME	Focal spikes (EEG)	(Lee et al., 2014)
I1616T (Paternal)	DIVS3	Missense	PEFS+	NR	(Hattori et al., 2008; Shi et al., 2012)
M1619V (Maternal)	DIVS3	Missense	Intractable Epilepsy	NR	(Wang et al., 2012)
F1620V	DIVS3	Missense	GEFS+	NR	(Cetica et al., 2017)

L1624Q(Maternal)	DIVS3	Missense	Focal Family Epilepsy	NR	(Oates et al., 2018)
L1624P (Maternal)	DIVS3	Missense	FHM	Enhanced persistent current<br>Delayed fast inactivation<br>Faster recovery from inactivation<br>Shift steady state inactivation to more positive values	(Fan et al., 2016)
T1628X (De novo)	DIVS3	Nonsense	DS	NR	(Sun et al., 2010)
V1630M (Familial)	DIVS3-S4	Missense	DS	NR	(Marini et al., 2007)
V1630L (Maternal)	DIVS3-S4	Missense	SMEB	Acute encephalopathy<br>Moderate neurologic sequelae	(Okumura et al., 2012; Wang et al., 2012)
V1630G	DIVS3-S4	Missense	DS	NR	(Djémié et al., 2016)
S1631C	DIVS3-S4	Missense	DS	Multifocal spikes (EEG)	(Haginoya et al., 2018)
P1632S	DIVS3-S4	Missense	ICEGTC	Generalizes tonic-clonic<br>Severe mental delay	(Fujiwara, 2003)

T1633YfsX10	DIVS3-S4	FrameShift	Epilepsy	NR	(Lindy et al., 2018)
L1634CfsX4	DIVS3-S4	FrameShift	DS	Generalized tonic-clonic seizures<br><br>Focal tonic and myoclonic seizures	(Rilstone et al., 2012)
F1635L (Maternal)	DIVS4	Missense	GEFS+	NR	(Zhang et al., 2015)
R1636X (De novo)	DIVS4	Nonsense	SMEB<br>DS	NR	(Lim et al., 2011; Zuberi et al., 2011; Cetica et al., 2017; Tsai et al., 2018)
R1636Q(De novo)	DIVS4	Missense	DS<br>LGS	Epileptic encephalopathy<br><br>Myoclonic seizures<br><br>Dystonia, and spasticity	(Harkin et al., 2007; Butler et al., 2017b)
R1636P (De novo)	DIVS4	Missense	Malignant migrating partial seizures of infancy	NR	(Gokben et al., 2017)
V1637E (De novo)	DIVS4	Missense	DS	Episodes of status epilepticus triggered by fever- unresponsive to medications	(Nishri et al., 2010; Zuberi et al., 2011)
I1638VfsX11	DIVS4	FrameShift	DS	NR	(Depienne et al., 2008)

I1638T (De novo)	DIVS4	Missense	DS	NR	(Zuberi et al., 2011)
I1638N (De novo)	DIVS4	Missense	DS	NR	(Wang et al., 2012)
R1639G	DIVS4	Missense	DS	NR	(Depienne et al., 2008)
R1639VfsX 11	DIVS4	FrameShift	DS	NR	(Kumar et al., 2018)
R1639P	DIVS4	Missense	Epilepsy	NR	(Lindy et al., 2018)
A1641T (Mosaic)	DIVS4	Missense	DS	NR	(Yang et al., 2017)
R1642M	DIVS4	Missense	SME	Generalized tonic-clonic seizures	(Lee et al., 2014)
R1642S (De novo)	DIVS4	Missense	DS	NR	(Wang et al., 2012)
Il1643LfsX 7 (De novo)	DIVS4	FrameShift	SME	NR	(Gaily et al., 2013)
R1642S	DIVS4	Missense	Epilepsy	NR	(Lindy et al., 2018)

G1644D	DIVS4	Missense	Epilepsy	NR	(Lindy et al., 2018)
R1645X (De novo)	DIVS4	Nonsense	DS	Generalized spike and wave, polyspike and wave discharges with photosensitivity (EEG)	(Fukuma et al., 2004; Harkin et al., 2007; Marini et al., 2007; Gökben et al., 2009; Zuberi et al., 2011; Wang et al., 2012; Cho et al., 2018)
R1645Q (De novo)	DIVS4	Missense	DS	NR	(Harkin et al., 2007; Oates et al., 2018)
R1645P	DIVS4	Missense	DS	NR	(Xu et al., 2015)
R1648C	DIVS4	Missense	DS	Increase current density<br>Shift steady state activation to more positive values<br>Shift steady state inactivation to more negative values	(Ohmori et al., 2002; Rhodes et al., 2005; Orrico et al., 2009; Zuberi et al., 2011)
R1648H (Paternal)	DIVS4	Missense	GEFS+	Enhanced persistent current<br>Delayed inactivation	(Alekov et al., 2000; Escayg et al., 2000; Spampanato et al., 2001; Rhodes et al., 2004; Depienne et al., 2008)
R1648L	DIVS4	Missense	Epilepsy	NR	(Lindy et al., 2018)
L1649Q(Paternal)	DIVS4	Missense	FHM	NR	(Vanmolkot et al., 2007)

K1651QfsX22 (De novo)	DIVS4	FrameShift	SMEB	NR	(Harkin et al., 2007)
G1652X	DIVS4	Nonsense	DS	NR	(Depienne et al., 2008)
A1653E	DIVS4	Missense	SMEB	NR	(Zuberi et al., 2011)
I1656S (De novo)	DIVS4	Missense	DS	NR	(Usluer et al., 2016)
I1656M (Maternal)	DIVS4	Missense	GEFS+	Shift steady state activation to more positive values	(Lossin et al., 2003)
R1657C (Maternal)	DIVS4	Missense	DS	Decrease current density<br>Shift steady state activation to more positive values<br>Faster recovery from slow inactivation	(Lossin et al., 2003)
R1657H	DIVS4	Missense	GEFS+	Febrile seizures<br>Focal seizures<br>Generalized tonic-clonic seizures	(Harkin et al., 2007; Catarino et al., 2011)
T1658R (De novo; Maternal)	DIVS4-S5	Missense	SMEB<br>DS	NR	(Marini et al., 2007; Depienne et al., 2008)
T1658M (Maternal)	DIVS4-S5	Missense	DS<br>SME	NR	(Depienne et al., 2008)

L1659M	DIVS4-S5	Missense	DS	NR	(Cetica et al., 2017)
L1660P	DIVS4-S5	Missense	SME<br>DS	NR	(Zuberi et al., 2011; Rodda et al., 2012)
F1661L (Maternal)	DIVS4-S5	Missense	FHM	NR	(Weller et al., 2014)
F1661S (De novo)	DIVS4-S5	Missense	DS	Reduce current density<br>Enhandec persistent current	(Claes et al., 2003; Rhodes et al., 2004)
A1662V	DIVS4-S5	Missense	SMEB	NR	(Wang et al., 2012)
L1663FfsX10 (De novo)	DIVS4-S5	FrameShift	DS	NR	(Zuberi et al., 2011)
M1664K (Maternal)	DIVS4-S5	Missense	DS	Reduced current density (92%) associeted with reduction of cell surface expression (95%)	(Depienne et al., 2008; Bechi et al., 2015)
S1666fsX7	DIVS4-S5	FrameShift	SME	NR	(Xu et al., 2014)
M1665_S1666insIle (De novo)	DIVS4-S5	In-frame Insertion	DS	NR	(Yang et al., 2017)

M1665I	DIVS4-S5	Missense	Epilepsy	NR	(Lindy et al., 2018)
S1666F	DIVS4-S5	Missense	DS	NR	(Xu et al., 2015)
L1667P (De novo)	DIVS4-S5	Missense	DS	NR	(Zuberi et al., 2011; Wang et al., 2012)
P1668A (Maternal)	DIVS4-S5	Missense	DS	NR	(Nabbout et al., 2003)
P1668R(Maternal)	DIVS4-S5	Missense	SME	NR	(Huang et al., 2014)
P1668fsX12	DIVS4-S5	FrameShift	SME	NR	(Xu et al., 2014)
P1668L	DIVS4-S5	Missense	SMEB	NR	(Zuberi et al., 2011)
A1669T	DIVS4-S5	Missense	NR	Moderate/Severe developmental delay<br>Epilepsy with continuous spikes and waves during sleep (EEG)	(Fry et al., 2016)
A1669E	DIVS4-S5	Missense	Malignant migrating partial seizures of infancy	Left arm clonic activity<br>Microcephaly (autopsy)	(Freilich et al., 2011)

L1670W (Maternal)	DIVS4-S5	Missense	FHM3	Reduced current density<br>Shift steady state activation and inactivation to more positive values<br>Incomplete inactivation<br>faster recovery from fast inactivation<br>Resistence to entry into closed state inactivation	(Zhang et al., 2017; Dhifallah et al., 2018; Bertelli et al., 2019)
F1671fsX8 (De novo)	DIVS4-S5	FrameShift	DS	Gerenalized tonic-clonic seizures<br>Severe mental retardation	(Claes et al., 2001; Sugawara et al., 2002; Depienne et al., 2008; Riva et al., 2009)
N1672H	DIVS4-S5	Missense	SME	NR	(Xu et al., 2014)
N1672I	DIVS4-S5	Missense	DS	NR	(Zuberi et al., 2011)
I1673T	DIVS5	Missense	DS<br>GEFS+	Genelized tonic-clonic seizure with fever	(Skjei et al., 2015)
I1673N	DIVS5	Missense	DS	NR	(Cetica et al., 2017)
G1674S (Maternal)	DIVS5	Missense	FS+<br>Hemiconvulsions hemiplegia epilepsy	Febrile seizure<br>Hemiconvulsion	(Saitoh et al., 2015a)
G1674R(De novo)	DIVS5	Missense	DS	NR	(Ohmori et al., 2002; Depienne et al., 2008)

Leu1675P (De novo)	DIVS5	Missense	DS	NR	(Yang et al., 2017)
L1677F	DIVS5	Missense	DS	NR	(Depienne et al., 2008; Lim et al., 2011; Wang et al., 2012)
M1681CfsX 34	DIVS5	FrameShift	DS	NR	(Depienne et al., 2008)
M1681Kfs X35	DIVS5	Frame Shift	DS	NR	(Riva et al., 2009)
F1682S	DIVS5	Missense	DS	Severe psycomotor retardation	(Fukuma et al., 2004)
I1683T (De novo)	DIVS5	Missense	DS<br>SMEB	NR	(Zuberi et al., 2011; Wang et al., 2012; Lee et al., 2014)
Y1684D	DIVS5	Missense	DS	NR	(Zuberi et al., 2011)
Y1684S	DIVS5	Missense	Intractable Epilepsy	NR	(Wang et al., 2012)
Y1684X (De novo)	DIVS5	Nonsense	SMEB	NR	(Zuberi et al., 2011)
A1685D(De novo)	DIVS5	Missense	DS	Spike-wave complex (EEG)<br>Non-fuctional channel	(Fujiwara, 2003) (Sugiura et al., 2012)

A1685V	DIVS5	Missense	PEFS+	Frequent seizures, both febrile and afebrile, during infancy and subsequently developed epilepsies<br><br>Non functional channel	(Sugawara et al., 2001) (Sugiura et al., 2012)
F1687S (Familial)	DIVS5	Missense	Myiclonic astatic epilepsy<br>GEFS+	NR	(Marini et al., 2007; Cetica et al., 2017)
G1688W (De novo)	DIVS5	Missense	DS	NR	(Zuberi et al., 2011)
G1688V	DIVS5	Missense	DS	NR	(Xu et al., 2015)
M1689R	DIVS5	Missense	DS	NR	(Ishii et al., 2017)
S1690AfsX 25	DIVS5	FrameShift	DS	NR	(Ream and Mikati, 2014)
F1692S (De novo)	DIVS5	Missense	DS	NR	(Wang et al., 2012)
Y1694YfsX 22 (De novo)	DIVS5	FrameShift	DS	NR	(Zuberi et al., 2011)
Y1694C (De novo)	DIVS5	Missense	DS	Myoclonic seizures<br>Atypical absence	(Fukuma et al., 2004; Wang et al., 2012; Cetica et al., 2017)

				Severe psychomotor retardation	
Y1694X	DIVS5	Nonsense	DS	NR	(Xu et al., 2015)
V1695LfsX 20 (De novo)	DIVS5	FrameShift	DS	NR	(Depienne et al., 2008)
K1696EfsX 6 (De novo)	DIVS5	FrameShift	DS	NR	(Orrico et al., 2009)
I1701DfsX3 (De novo)	DIVS5-S6	FrameShift	DS	NR	(Wang et al., 2012)
D1702H	DIVS5-S6	Missense	DS	NR	(Cetica et al., 2017)
D1702Y	DIVS5-S6	Missense	Epilepsy	NR	(Lindy et al., 2018)
D1702E	DIVS5-S6	Missense	SME	NR	(Xu et al., 2014)
D1703Y	DIVS5-S6	Missense	DS	NR	(Ishii et al., 2017)
D1703V	DIVS5-S6	Missense	SME	NR	(Xu et al., 2014)

F1707V	DIVS5-S6	Missense	DS	NR	(Harkin et al., 2007; Rodda et al., 2012)
F1707L	DIVS5-S6	Missense	Epilepsy	NR	(Lindy et al., 2018)
T1709P	DIVS5-S6	Missense	DS	NR	(Haginoya et al., 2018)
T1709I (Maternal)	DIVS5-S6	Missense	ICEGTC	Spike-wave complex and multifocal spikes (EEG)<br>Non-functional channel	(Fujiwara, 2003) (Rhodes et al., 2005)
T1709N	DIVS5-S6	Missense	DS	NR	(Cetica et al., 2017)
G1711S	DIVS5-S6	Missense	Epilepsy	NR	(Lindy et al., 2018)
N1712K	DIVS5-S6	Missense	DS	NR	(Ishii et al., 2017)
S1713N	DIVS5-S6	Missense	DS	Focal spikes in the right parietal region; sharp waves over the left frontal region in the sleep record (EEG)	(Kimura et al., 2005)
A1713D (De novo)	DIVS5-S6	Missense	DS	Febrile seizures, Hemiclonic, generalized tonic clonic seizures<br>Focal spike-waves complex (EEG)	(Liu et al., 2018)

M1714V (Maternal)	DIVS5-S6	Missense	GEFS+	NR	(Tsai et al., 2018)
M1714R (De novo)	DIVS5-S6	Missense	DS	NR	(Riva et al., 2009; Zuberi et al., 2011)
M1714K	DIVS5-S6	Missense	DS	NR	(Depienne et al., 2008)
C1716R (De novo)	DIVS5-S6	Missense	SMEB Epilepsy and/or NDD	Febril convulsios Afebril concursions Parcial seizures Generalized spikes-wave (EEG)	(Marini et al., 2007; Lindy et al., 2018)
L1717P (De novo)	DIVS5-S6	Missense	SME	Generalized tonic clonic seizure	(Verbeek et al., 2013)
T1721R (De novo)	DIVS5-S6	Missense	DS	NR	(Harkin et al., 2007)
T1721K	DIVS5-S6	Missense	Ep and/or NDD	Seizures	(Retterer et al., 2016; Lindy et al., 2018)
I1722FfsX46	DIVS5-S6	Frameshift	DS	Not reported	(Ko et al., 2018)
T1722A (De novo)	DIVS5-S6	Missense	DS	Myoclonic, hemiclonic Focal seizures	(Wu et al., 2015)

S1723F	DIVS5-S6	Missense	Ep and/or NDD	NR	(Lindy et al., 2018)
A1724P	DIVS5-S6	Missense	Intractable Epilepsy	NR	(Wang et al., 2012)
A1724T (De novo)	DIVS5-S6	Missense	DS	NR	(Ishii et al., 2017)
G1725C (De novo)	DIVS5-S6	Missense	DS	NR	(Depienne et al., 2008)
W1726R (De novo)	DIVS5-S6	Missense	DS	NR	(Harkin et al., 2007)
W1726X	DIVS5-S6	Nonsense	DS	NR	(Zuberi et al., 2011)
G1728S.fsX 4	DIVS5-S6	Frameshift	Ep and/or NDD	NR	(Lindy et al., 2018)
D1727G (De novo)	DIVS5-S6	Missense	DS	NR	(Wang et al., 2012)
L1729W (De novo)	DIVS5-S6	Missense	DS	NR	(Ishii et al., 2017)

L1730P	DIVS5-S6	Missense	Ep and/or NDD	NR	(Lindy et al., 2018)
A1731P<br>(De novo)	DIVS5-S6	Missense	DS	NR	(Ishii et al., 2017)
I1733FfsX46	DIVS5-S6	Frameshift	DS	Generalized tonic clonic seizure<br>Atonic, complex partial and absence seizures	(de Lange et al., 2018)
L1729FfsX9<br>(De novo)	DIVS5-S6	Frameshift	DS	NR	(Depienne et al., 2008)
P1732L<br>(De novo)	DIVS5-S6	Missense	DS	NR	(Djémié et al., 2016)
N1733D	DIVS5-S6	Missense	DS<br>GEFS+	Generalized tonic clonic seizure	(Tan et al., 2012)
L1734P	DIVS5-S6	Missense	Ep and/or NDD	NR	(Lindy et al., 2018)
P1739L	DIVS5-S6	Missense	GEFS+<br>FS+	NR	(Zuberi et al., 2011)
D1740N	DIVS5-S6	Missense	FS+/FS+ and focal seizures<br>Ep and/or NDD	NR	(Lindy et al., 2018)

C1741R (De novo)	DIVS5-S6	Missense	DS	NR	(Wang et al., 2012)
C1741S (De novo)	DIVS5-S6	Missense	TLT-MTS	Febrile status epilepticus	(Tiefes et al., 2019)
C1741S (De novo)	DIVS5-S6	Missense	LGS	NR	(Allen et al., 2013)
C1741Y	DIVS5-S6	Missense	DS<br>Ep and/or NDD	NR	(Xu et al., 2015; Lindy et al., 2018)
D1742G	DIVS5-S6	Missense	PEFS+	Myoclonic astatic seizures<br>Febrile seizures<br>Generalized tonic-clonic seizure	(Pineda-Trujillo et al., 2005)
G1746W	DIVS5-S6	Missense	DS	NR	(Esterhuizen et al., 2018)
1747Nfs1779X	DIVS5-S6	Frameshift	DS	NR	(Margherita Mancardi et al., 2006)
G1749E (De novo)	DIVS5-S6	Missense	DS	Generalized tonic-clonic seizures<br>Complex partial seizures<br>Reduced current density	(Claes et al., 2003; Rhodes et al., 2005)

S1750fsX1778 (De novo)	DIVS5-S6	Deletion and insertion	DS	NR	(Sun et al., 2010)
S1751GfsX29	DIVS5-S6	Frameshift	DS	NR	(Xu et al., 2015)
G1754V	DIVS5-S6	Missense	Ep and/or NDD	NR	(Lindy et al., 2018)
G1754R (De novo)	DIVS5-S6	Missense	DS	Focal seizures Hemiconvulsions	(Petrelli et al., 2012)
D1755G	DIVS5-S6	Missense	SME	NR	(Xu et al., 2014)
C1756G	DIVS5-S6	Missense	SMEB	Febrile seizures with generalized tonic clonic seizure	(Herini et al., 2010)
G1757R (De novo)	DIVS5-S6	Missense	DS	NR	(Djémié et al., 2016)
G1757E	DIVS5-S6	Missense	Ep and/or NDD	NR	(Lindy et al., 2018)
N1758IfsX35	DIVS5-S6	Frameshift	Ep and/or NDD	NR	(Lindy et al., 2018)

F1764fsX17	DIVS6	Frameshift	SME	NR	(Xu et al., 2014)
G1762E (De novo)	DIVS6	Missense	DS	NR	(Margherita Mancardi et al., 2006)
I1763F De novo	DIVS6	Missense	DS	NR	(Zuberi et al., 2011)
I1763N De novo	DIVS6	Missense	DS	NR	(Zuberi et al., 2011)
F1764Y	DIVS6	Missense	Ep and/or NDD	NR	(Lindy et al., 2018)
F1765fsX1794	DIVS6	Frameshift	DS	Generalized tonic-clonic seizures Multifocal spikes (EEG)	(Fujiwara, 2003)
F1765L De novo	DIVS6	Missense	PEFS+	Complete absence of sodium current	(Liao et al., 2010)
F1765delX1777 De novo	DIVS6	Frameshift	DS	Febrile convulsions Generalized spike-wave (EEG)	(Marini et al., 2007)

F1756del	DIVS6	In-frame deletion	SMEB	NR	(Fukuma et al., 2004)
F1766CfsX28	DIVS6	Frameshift	DS	NR	(Xu et al., 2015)
S1768R<br><br>De novo	DIVS6	Missense	DS	Absences and tonic-clonic seizures	(Willemsen et al., 2012)
I1770F	DIVS6	Missense	DS	NR	(Zuberi et al., 2011)
I1770HfsX24	DIVS6	Frameshift	Ep and/or NDD	NR	(Lindy et al., 2018)
I1770N	DIVS6	Missense	DS<br><br>Secondary epilepsy	Encephalopathic<br>Movement disorder<br>Cognitive regression	(Tang et al., 2011)
I1770T	DIVS6	Missense	SMEB	NR	(Zuberi et al., 2011)
I1771N	DIVS6	Missense	DS	NR	(Depienne et al., 2008)
A1772T<br><br>De novo	DIVS6	Missense	DS	Febrile seizures<br>Focal to bilateral tonic-clonic<br>Focal spike wave (EEG)	(Esterhuizen et al., 2018)

S1773F<br>De novo	DIVS6	Missense	DS	NR	(Margherita Mancardi et al., 2006)
F1774S	DIVS6	Missense	HM3	Enhanced persintet current	(Chastan et al., 2016; Bertelli et al., 2019)
L1775P	DIVS6	Missense	DS	NR	(Jiménez-Arredondo et al., 2017)
V1776F	DIVS6	Missense	DS	NR	(Ishii et al., 2017)
M1780T<br>De novo	DIVS6	Missense	DS	NR	(Nabbout et al., 2003)
Y1781H<br>De novo	DIVS6	Missense	DS	NR	(Depienne et al., 2008)
Y1771C	DIVS6	Missense	DS	NR	(Fukuma et al., 2004)
I1782S	DIVS6	Missense	DS	NR	(Depienne et al., 2008)

I1782M	DIVS6	Missense	DS<br>SME	Febrile and hemiclonic seizures	(Villeneuve et al., 2014)
A1783T<br>De novo	DIVS6	Missense	DS<br>SME<br>DS	Generalized tonic–clonic seizures<br>Myoclonic seizures<br>Complex partial seizures<br>Clonic seizures	(Sun et al., 2010; Klassen et al., 2011; Petrelli et al., 2012; Moehring et al., 2013; Lindy et al., 2018)
A1783V<br>De novo	DIVS6	Missense	DS<br>Epilepsy and/or NDD	Febril convulsions<br>Afebril convulsions<br>Myoclonic seizures<br>Parcial seizures<br>Generalized spikes-wave and focal (EEG)	(Marini et al., 2007; Klassen et al., 2011; Petrelli et al., 2012; Wang et al., 2012; Butler et al., 2017a)
I1785fsX11	DIVS6	Frameshift	SME	NR	(Xu et al., 2014)
E1787K	DIVS6	Missense	DS	Febril convulsions<br>Generalized spikes-wave and multifocal (EEG)	(Marini et al., 2007)
E1787D	DIVS6	Missense	Ep and/or NDD	NR	(Lindy et al., 2018)
N1788fsX1796	C-terminal	Frameshift	DS	NR	(Wallace et al., 2003)
N1788K(;) H939P	C-terminal	Missense<br>Compound	DS	NR	(Depienne et al., 2008)

F1789LfsX5	C-terminal	Frameshift	DS	NR	(Marini et al., 2007)
A1792T De novo	C-terminal	Missense	DS	NR	(Wang et al., 2012)
A1792D	C-terminal	Missense	Ep and/or NDD	NR	(Lindy et al., 2018)
E1795Q	C-terminal	Missense	Ep and/or NDD	NR	(Lindy et al., 2018)
E1795G De novo	C-terminal	Missense	Epilepsy	NR	(Staněk et al., 2018)
A1797P	C-terminal	Missense	Tonic-clonic + focal seizures	Tonic-clonic and focal seizures	(Butler et al., 2017a)
A1797EfsX4	C-terminal	Frameshift	DS	NR	(Cho et al., 2018)
S1801RfsX56	C-terminal	Frameshift	Ep and/or NDD	NR	(Lindy et al., 2018)

E1802X	C-terminal	Nonsense	DS	NR	(Xu et al., 2015)
F1805X<br>De novo	C-terminal	Frameshift	DS	NR	(Nabbout et al., 2003)
E1806X	C-terminal	Nonsense	Ep and/or NDD	NR	(Lindy et al., 2018)
1807delMFYE	C-terminal	In-frame deletion	DS	Generalized tonic clonic seizures	(Fujiwara, 2003)
F1808L	C-terminal	Missense	ICEGTC	Reduced current density<br>Shift steady state activation and inactivation to more negative values	(Fujiwara, 2003; Rhodes et al., 2005)
F1808I<br>De novo	C-terminal	Missense	SME	NR	(Depienne et al., 2008)
W1812G<br>De novo	C-terminal	Missense	DS<br>Epilepsy and/or NDD	Generalized tonic clonic seizures<br>Multifocal spikes (EEG)	(Fujiwara, 2003; Skjei et al., 2015; Lindy et al., 2018)
W1812R	C-terminal	Missense	Ep and/or NDD	NR	(Lindy et al., 2018)

W1812S<br>De novo	C-terminal	Missense	DS	NR	(Depienne et al., 2008)
W1812X<br>De novo	C-terminal	Nonsense	GEFS+	NR	(Cetica et al., 2017)
W1812C (;)<br>1813–<br>1815EFK<br>De novo	C-terminal	In-frame deletion	DS	NR	(Nabbout et al., 2003)
E1813_F18<br>15del<br>De novo	C-terminal	In-frame deletion	SME	NR	(Depienne et al., 2008)
Q1815K<br>De novo	C-terminal	Missense	EE	Febrile and afebrile seizures<br>Generalized tonic clonic seizures<br>Generalized spikes and waves (EEG)	(Mercimek-Mahmutoglu et al., 2015)
A1819S	C-terminal	Missense	GEFS+	NR	(Cetica et al., 2017)
Q1821X<br>De novo	C-terminal	Nonsense	SME	NR	(Djémié et al., 2016)
M1823R<br>(De novo)	C-terminal	Missense	DS	NR	(Ishii et al., 2017)

E1824X	C-terminal	Nonsense	SME	NR	(Xu et al., 2014)
L1828S	C-terminal	Missense	Neurodevelopmental disorder, epilepsy-related	NR	(Stosser et al., 2018)
Q1830X<br>De novo	C-terminal	Nonsense	DS	NR	(Zuberi et al., 2011)
F1831S<br>De novo	C-terminal	Missense	DS	Generalized tonic clonic seizures<br>Ataxia<br>Severe mental decline<br>Small current density reduction	(Sugawara et al., 2002; Fujiwara, 2003; Le Gal et al., 2014)
F1831LfX27	C-terminal	Frameshift	DS	NR	(Depienne et al., 2008)
A1832P	C-terminal	Missense	DS	NR	(Zuberi et al., 2011)
L1835F<br>De novo	C-terminal	Missense	DS	NR	(Depienne et al., 2008)
L1835_P1837del	C-terminal	In-frame deletion	Ep and/or NDD	NR	(Lindy et al., 2018)

E1836GfsX 25	C-terminal	Frameshift	DS	NR	(Depienne et al., 2008)
Q1837X	C-terminal	Nonsense	DS	NR	(Wang et al., 2012)
L1839V	C-terminal	Missense	SME	NR	(Xu et al., 2014)
L1839P<br>De novo	C-terminal	Missense	DS	Generalized tonic-clonic seizures<br>Hemiclonic and focal seizures	(Wu et al., 2015)
L1841P	C-terminal	Missense	DS	Focal seizures	(Kumar et al., 2018)
M1845Hfs X5	C-terminal	Frameshift	DS	NR	(Ko et al., 2018)
K1846S.fsX 11<br>De novo	C-terminal	Frameshift	DS<br>SMEB	Generalized tonic clonic seizures	(Wallace et al., 2003; Margherita Mancardi et al., 2006; Zucca et al., 2008; Nicita et al., 2010; Sun et al., 2010; Petrelli et al., 2012; Lee et al., 2014; Haginoya et al., 2018)
K1846X	C-terminal	Nonsense	DS	NR	(Sun et al., 2010)
Q1848X	C-terminal	Nonsense	DS	NR	(Ishii et al., 2017)

M1852K	C-terminal	Missense	DS	NR	(Zuberi et al., 2011)
M1852T	C-terminal	Missense	DS<br>GEFS+	Reduced current density	(Xu et al., 2015)
M1852IfsX9	C-terminal	Frameshift	Intractable Epilepsy	NR	(Wang et al., 2012)
P1855S	C-terminal	Missense	Epilepsy	NR	(Butler et al., 2017a)
P1855L<br>De novo	C-terminal	Missense	DS	NR	(Zuberi et al., 2011)
M1856T	C-terminal	Missense	FS+<br>Epilepsy and/or NDD	NR	(Lindy et al., 2018)
M1856K	C-terminal	Missense	Ep and/or NDD	NR	(Lindy et al., 2018)
V1857L	C-terminal	Missense	GEFS+	Febrile seizures<br>Generalized tonic–clonic convulsion	(Nagao et al., 2005)
G1859WfsX2	C-terminal	Frameshift	DS	NR	(Depienne et al., 2008)

R1861W	C-terminal	Missense	Intractable Epilepsy	NR	(Wang et al., 2012)
R1861P	C-terminal	Missense	Ep and/or NDD	NR	(Lindy et al., 2018)
D1866Y	C-terminal	Missense	GEFS+	Shift steady state inactivation to more positive values<br>Enhanced persistent current	(Spampanato, 2004)
D1866G<br>(De novo)	C-terminal	Missense	DS	NR	(Ishii et al., 2017)
I1867NfsX78	C-terminal	Frameshift	DS	NR	(Depienne et al., 2008)
I1867T	C-terminal	Missense	GEFS+	Generalized tonic–clonic seizures	(Hindocha et al., 2008)
I1867TfsX10<br>De novo	C-terminal	Frameshift	DS	NR	(Depienne et al., 2008)
T1872fsX	C-terminal	Frameshift	DS	Secondary generalized tonic–clonic seizures<br>Focal tonic seizures	(Rilstone et al., 2012)

L1876P	C-terminal	Missense	DS<br><br>Epilepsy and/or NDD	NR	(Lindy et al., 2018)
D1876X	C-terminal	Frameshift	DS	NR	(Zuberi et al., 2011)
G1877V	C-terminal	Missense	GEFS+	Febrile convulsions with non-febrile seizures<br>Psychomotor delay<br>Generalize tonic clonic/complex seizures	(Kim and Lee, 2017)
S1879fsX31<br>De novo	C-terminal	Frameshift	SME	Febrile seizures<br>Generalized tonic–clonic seizures	(Verbeek et al., 2013)
G1880E	C-terminal	Missense	DS	NR	(Zuberi et al., 2011)
E1881K	C-terminal	Missense	DS	Focal seizures	(Petrelli et al., 2012)
E1881X<br>De novo	C-terminal	Nonsense	DS<br>SMEB	Febrile and generalized seizures	(Villeneuve et al., 2014)
E1881D<br>De novo	C-terminal	Missense	DS<br>SME	NR	(Wallace et al., 2003; Rodda et al., 2012)

R1886X<br>De novo	C-terminal	Nonsense	DS	Febrile and hemiclonic seizures	(Margherita Mancardi et al., 2006; Villeneuve et al., 2014; Djémié et al., 2016)
R1886fsX1910<br>De novo	C-terminal	Frameshift	DS	NR	(Nabbout et al., 2003)
R1886Q	C-terminal	Missense	SME	Generalized tonic–clonic seizures<br>Atypic absences	(Moehring et al., 2013)
E1890fsX1910<br>De novo	C-terminal	Frameshift	DS	NR	(Nabbout et al., 2003)
F1893AfsX53	C-terminal	Frameshift	DS	Febrile and generalized seizures	(Villeneuve et al., 2014)
R1892X<br>De novo	C-terminal	Nonsense	DS	Reduced current density	(Sugawara et al., 2002; Nicita et al., 2010)
R1892G	C-terminal	Missense	Epilepsy	NR	(Afawi et al., 2016)
P1894L<br>De novo	C-terminal	Missense	EP+ASD	NR	(O'Roak et al., 2011)

P1898LfsX13<br>De novo	C-terminal	Frameshift	DS	Febrile and myoclonic seizures<br>Secondary generalized tonic-clonic seizures	(Zhou et al., 2018)
Q1904X	C-terminal	Nonsense	DS<br><br>Epilepsy and/or NDD	NR	(Lindy et al., 2018)
Q1904fsX42	C-terminal	Frameshift	DS	NR	(Sugawara et al., 2002)
T1907fsX4	C-terminal	Frameshift	SME	NR	(Xu et al., 2014)
T1909I	C-terminal	Missense	DS	Reduced current density<br>Enhanced persistent current	(Ohmori et al., 2006)
T1909del<br>De novo	C-terminal	In-frame deletion	DS	NR	(Zuberi et al., 2011)
R1912KfsX32<br>De novo	C-terminal	Frameshift	DS	NR	(Zuberi et al., 2011)
R1912X<br>De novo	C-terminal	Nonsense	DS	Generalized tonic–clonic seizures<br>Absense, myoclonic, partial and hemiclonic seizures	(Ozmen et al., 2011)

K1913X	C-terminal	Nonsense	Ep and/or NDD	NR	(Lindy et al., 2018)
p.1914_1914del<br>De novo	C-terminal	In-frame deletion	DS	Febrile seizures<br>Myoclonic seizures<br>Focal to bilateral tonic-clonic<br>Multifocal discharges (EEG)	(Liu et al., 2018)
Q1914del<br>De novo	C-terminal	In-frame deletion	Pediatric refractory epilepsy	NR	(Liu et al., 2018)
Q1914RfsX30<br>De novo	C-terminal	Frameshift	ICEGTC	NR	(Harkin et al., 2007)
E1915X	C-terminal	Frameshift	DS	Febrile and hemiclonic seizures	(Villeneuve et al., 2014)
E1915K	C-terminal	Missense	Ep and/or NDD	NR	(Lindy et al., 2018)
E1915G.fsX28<br>De novo	C-terminal	Frameshift	DS	NR	(Ream and Mikati, 2014)
E1916KfsX16	C-terminal	Frameshift	Ep and/or NDD	NR	(Lindy et al., 2018)

E1916X	C-terminal	Nonsense	Ep and/or NDD	NR	(Lindy et al., 2018)
S1918F	C-terminal	Missense	Ep and/or NDD	NR	(Lindy et al., 2018)
I1922T	C-terminal	Missense	DS	NR	(Harkin et al., 2007; Mulley et al., 2013; Lindy et al., 2018)
Q1923R	C-terminal	Missense	PEFS+	Secondarily generalized tonic-clonic and complex partial seizures<br>Focal spike-waves and generalized spike-wave (EEG)	(Shi et al., 2012)
R1924LfsX8<br>De novo	C-terminal	Frameshift	DS	NR	(Djémié et al., 2016)
A1925T	C-terminal	Missense	FS+	NR	(Møller et al., 2016)
Y1926X	C-terminal	Nonsense	DS	NR	(Zuberi et al., 2011)
R1927delinsIle,Ile,Gln<br>De novo	C-terminal	Frameshift	DS	NR	(Zuberi et al., 2011)
R1927T	C-terminal	Missense	DS	NR	(Xu et al., 2015)

R1928G	C-terminal	Missense	DS	NR	(Zucca et al., 2008)
L1930FfsX2<br>De novo	C-terminal	Frameshift	SMEB	NR	(Zuberi et al., 2011)
K1936E	C-terminal	Missense	Ep and/or NDD	NR	(Lindy et al., 2018)
A1951S	C-terminal	Missense	DS	NR	(Cetica et al., 2017)
E1957G	C-terminal	Missense	Infantile spasms<br>DS	NR	(Wallace et al., 2003; Cherepanova et al., 2013)
M1977L	C-terminal	Missense	GEFS+	Generalized convulsions<br>Afebrile seizures<br>Bilateral frontal spike-waves (EEG)	(Saitoh et al., 2012)
R1988W	C-terminal	Missense	Epilepsy-aphasia with FS+	NR	(Carvill et al., 2013)
del SCN1A<br>De novo	5'UTR	Partial gene deletion<br>(73.8Kb)	DS	Generalized tonic clonic seizures<br>Myoclonic seizures<br>Atypical absences<br>Focal seizures<br>Status epilepticus	(Nakayama et al., 2010)

del SCN1A,7A, 9A	5'UTR	Partial gene deletion	SMEB	Generalized tonic clonic seizures<br>Focal seizures<br>Status epilepticus<br>Secondary generalization	(Nakayama et al., 2010)
del SCN1A	N-terminal	Partial gene deletion	DS	NR	(Zuberi et al., 2011)
del SCN1A	Chromosome	Partial gene deletion<br>(Exon 12-14)	DS	Febrile convulsions<br>Absence seizures<br>Multifocal spike-waves (EEG)	(Altarescu et al., 2012)
del SCN1A	Chromosome	Partial gene deletion<br>(Exon 1 a 20)	SMEB	NR	(Lim et al., 2011)
del SCN1A	Chromosome	Whole gene deletion<br>(0.2 Mb)	DS	Tonic-clonic seizure<br>Hemiclonic seizure	(Lim et al., 2015)
del SCN1A	Chromosome	Partial gene deletion<br>(Exons 2–4)	DS<br>FS+	Febrile seizures<br>Febrile seizures plus<br>Generalized clonic seizures<br>Generalized tonic–clonic seizures	Guerrini R.2010
del SCN1A	Chromosome	Partial gene deletion<br>(Exons 2–16)	SFE	Focal seizures<br>Status epilepticus<br>Secondary generalization	(Nakayama et al., 2010)
del SCN1A<br>De novo	Chromosome	Partial gene deletion<br>(exon 8-26)	DS	NR	(Wang et al., 2008)

del SCN1A	Chromosome	Partial gene deletion (Exons 7 - 9)	DS	NR	(Depienne et al., 2008)
del SCN1A	Chromosome	Partial gene deletion (Exon 9)	DS	NR	(Depienne et al., 2008)
del SCN1A	Chromosome	Partial gene deletion (Exons 14 – 20)	DS	NR	(Depienne et al., 2008)
del SCN1A	Chromosome	Partial gene deletion (Exons 22 - 26)	DS	NR	(Depienne et al., 2008)
del SCN1A	Chromosome	Partial gene deletion (Exons 24)	DS	NR	(Depienne et al., 2008)
dup SCN1A	Chromosome	Partial gene duplication (Exons 2-21)	DS	NR	(Depienne et al., 2008)
del SCN1A	Chromosome DI-DII	Partial gene deletion	SME	NR	(Hattori et al., 2008)
del SCN1A	Chromosome	Partial gene deletion	DS	Generalized tonic–clonic seizures Absense, myoclonic and partial seizures	(Ekici et al., 2011)

del<br>De novo	Chromosome	Partial gene deletion<br>(exons 3 and 17–26)	DS	Tonic clonic seizures<br>Myoclonic seizures<br>Atypical absences<br>Focal seizures<br>Status epilepticus<br>Seizures provoked by fever	(Nakayama et al., 2010)
del SCN1A	Chromosome	Partial gene deletion<br>(Exons 17-20)	DS	Focal myoclonus seizures<br>Generalized tonic–clonic seizures	(Skjei et al., 2015)
del SCN1A<br>De novo	Chromosome	Partial gene deletion<br>(Exons 14–16)	DS	Generalized tonic clonic seizures<br>Myoclonic seizures<br>Atypical absences<br>Focal seizures<br>Status epilepticus<br>Seizures provoked by fever	(Nakayama et al., 2010)
del SCN1A	Chromosome	Partial gene deletion<br>(Exon 20)	SMEB	Generalized tonicclonic seizures<br>Focal seizures<br>Status epilepticus<br>Secondary generalization	(Nakayama et al., 2010)
del SCN1A<br>De novo	Chromosome	Partial gene deletion<br>(Exons 17-26)	DS	NR	(Wang et al., 2008)
del SCN1A<br>De novo	Chromosome	Partial gene deletion<br>(Exons 20-26)	DS	NR	(Wang et al., 2008)

del SCN1A<br>De novo	Chromosome	Partial gene deletion	DS	NR	(Sun et al., 2010)
del SCN1A<br>De novo	Chromosome	Whole gene deletion<br>(607 kb)	DS	Febrile and afebrile myoclonic seizures<br>Generalized spike–waves (EEG)	(Madia et al., 2006)
del SCN1A	Chromosome	Whole gene deletion<br>(0.750 Mb)	DS	NR	(Wang et al., 2008)
del SCN1A	Chromosome	Whole gene deletion<br>(1.139 Mb)	DS	NR	(Wang et al., 2008)
del SCN1A<br>De novo	Chromosome	Whole gene deletion<br>(1.277 Mb)	DS	NR	(Wang et al., 2008)
del SCN1A	Chromosome	Whole gene deletion<br>(1.5 Mb)	MMPSI	Focal clonic seizure	(Lim et al., 2015)
del SCN1A	Chromosome	Whole gene deletion<br>(1.549 Mb)	DS	NR	(Wang et al., 2008)
del SCN1A<br>De novo	Chromosome	Whole gene deletion<br>(3.1 Mb)	DS	Unilateral febrile clonic seizure<br>Febrile and afebrile myoclonic seizures	(Madia et al., 2006)

				Generalized spike–waves (EEG)	
del SCN1A<br>De novo	Chromosome	Whole gene deletion<br>(4 Mb)	DS	Myoclonic seizure<br>Light sensitivity	(Suls et al., 2006)
del SCN1A<br>De novo	Chromosome	Whole gene deletion<br>(4.5 Mb)	DS	Generalized tonic-clonic seizure	(Suls et al., 2006)
del SCN1A<br>De novo	Chromosome	Whole gene deletion<br>(4.7 Mb)	DS	Myoclonic or hemiclonic seizures<br>Generalized spike–waves (EEG)	(Madia et al., 2006)
del SCN1A	Chromosome	Whole gene deletion<br>(5 Mb)	DS and Leigh syndrome	Generalized tonic-clonic seizure	(Suls et al., 2006)
Del SCN1A, SCN2A, SCN1A, SCN3A, SCN9A and SCN7A	Chromosome	Deletion<br>(10.29 - 10.58 Mb)<br>(De novo interstitial deletion)	Severe epilepsy	Focal and generalized seizures<br>Lack of eye contact<br>Stereotypic and repetitive hand movements<br>Slow background with high amplitude delta waves mixed with spikes and sharp waves on the<br>temporo-occipital areas (EEG)	(Pescucci et al., 2007)

Del SCN1A, SCN2A, SCN3A, SCN7A and SCN9A	Chromosome	Deletion (10.4-Mb)	Severe epilepsy	Epileptic seizure with pale, atonic periods followed by a spasm-like out-throwing of both arms<br><br>Predominantly right-sided epileptiform activity (EEG)	(Davidsson et al., 2008)
del SCN1A (De novo)	Chromosome	Whole gene deletion (11.06 Mb)	MMPSI	Multifocal seizures<br>Status epilepticus	(Carranza Rojo et al., 2011)
del SCN1A, 9A, ABCB11 and LRP2 (De novo)	Chromosome	partial gene deletion	DS	NR	(Ma et al., 2016)
del SCN1A, 9A	Chromosome	whole gene deletion (0.2 Mb)	DS	Tonic-clonic seizure<br>Hemiclonic seizure	(Lim et al., 2015)
del SCN1A, 2A, 3A, 7A, 9A	Chromosome	partial gene deletion (8.4 Mb)	MMPSI	Tonic—clonic seizure<br>Multifocal seizures	(Lim et al., 2015)
del SCN1A De novo	Chromosome	partial gene deletion (Exon 7)	DS	>10 seizures/month	(Do et al., 2017)
del SCN1A, 2A, 3A, 7A, 9A	Chromosome	whole gene deletion (4.3 Mb)	MMPSI	Multifocal seizures<br>Hemiclonic seizure	(Lim et al., 2015)

del SCN1A (De novo)	Chromosome	partial gene deletion (Exon 8 – 26)	DS	NR	(Ishii et al., 2017)
del SCN1A (De novo)	Chromosome	partial gene deletion (Exon 17 – 26)	DS	NR	(Ishii et al., 2017)
del SCN1A (De novo)	Chromosome	partial gene deletion (Exon 20 – 26)	DS	NR	(Ishii et al., 2017)
del SCN1A (De novo)	Chromosome	partial gene deletion (Exon 1)	DS	NR	(Ishii et al., 2017)
del SCN1A De novo	Chromosome	partial gene deletion (Exon 5-8)	DS	NR	(Esterhuizen et al., 2018)
del SCN1A (De novo)	Chromosome	partial gene deletion (Exon 2-3)	DS	NR	(Ishii et al., 2017)
del SCN1A (De novo)	Chromosome	partial gene deletion (Exon 1-26)	DS	NR	(Ishii et al., 2017)
del SCN1A (De novo)	Chromosome	partial gene deletion (Exon 7)	DS	NR	(Ishii et al., 2017)
del SCN1A	Chromosome	partial gene deletion (Exon 1 a 13)	DS	NR	(Cetica et al., 2017)

del SCN1A	Chromosome	partial gene deletion (Exon 2 a 4)	DS FS	NR	(Cetica et al., 2017)
del SCN1A	Chromosome	partial gene deletion (Exon 1 a 26)	DS	NR	(Cetica et al., 2017)
del SCN1A	Chromosome	partial gene deletion (Exon 17 e 18)	DS	NR	(Cetica et al., 2017)
del SCN1A	Chromosome	partial gene deletion (Exons 2-23)	DS	Generalized tonic clonic seizure Focal, complex partial and tonic seizures	(de Lange et al., 2018)
dup SCN1A	Chromosome	Partial gene duplication	SME	NR	(Xu et al., 2014)
dup SCN1A	Chromosome	Partial gene duplication (Exons 2–16)	DS	Tonic clonic seizures Myoclonic seizures Atypical absences Focal seizures Status epilepticus Seizures provoked by fever	(Nakayama et al., 2010)
dup SCN1A De novo	Chromosome	Partial gene duplication	DS	NR	(Sun et al., 2010)

dup SCN1A	Chromosome	Partial gene duplication	SME	Generalized tonic clonic seizures	(Petrelli et al., 2012)
dup SCN1A<br><br>De novo	Chromosome	Partial gene duplication<br>(Exon 17 a 20)	DS	Afebrile seizures<br>Generalized tonic clonic seizures	(Verbeek et al., 2013)
Del SCN1A and SCN2A	Chromosome	whole gene deletion	IEE	NR	(Kwong et al., 2015)
dup SCN1A<br>De novo	Chromosome	Whole gene duplication	Neonatal seizure	Abnormal back- ground activities with spiky transients (EEG)	(Okumura et al., 2011)
dup SCN1A<br>De novo	Chromosome	Whole gene duplication<br>(6.7 Mb)	Epilepsy	Focal tonic seizures	(Goeggel Simonetti et al., 2012)
dup SCN1A<br>De novo	Chromosome	Whole gene duplication<br>(10.1 Mb)	Epilepsy	Multifocal clonic seizures	(Goeggel Simonetti et al., 2012)
dup SCN1A	Chromosome	Whole gene duplication	NIE	Facial flushing<br>Focal tonic seizures<br>Generalized tonic clonic seizures	(Raymond et al., 2011; Goeggel Simonetti et al., 2012)
dup SCN1A<br>De novo	Chromosome	Whole gene duplication<br>(11.19 Mb)	Epilepsy with cognitive impairment /ASD	NR	(Nicholl et al., 2013)

dup SCN1A	Chromosome	Partial gene duplication	GEFS+	NR	(Cetica et al., 2017)
translocation<br>De novo	Chromosome	t(2;5)(q24.3,q34)	DS	Afebrile epileptic seizure<br>Generalized convulsive seizure	(Møller et al., 2008)
unbalanced translocation<br>De novo	Chromosome	t(2;15)	Epilepsy, apnoea, mental retardation & dysmorphic features	Epileptic seizures accompanied by severe central apnea	(Langer et al., 2006)

Epilepsy (Ep); Generalized epilepsy with febrile seizures plus (GEFS+); Febrile seizures (FS); Febrile seizures plus (FS+); Lennox-Gastaut syndrome (LGS); Dravet syndrome (DS); Epileptic encephalopathy (EE); Borderline severe myoclonic epilepsy (SMEB); Severe myoclonic epilepsy (SME); Familial hemiplegic migraine (FHM); Partial epilepsy with antecedent FS (PEFS+); neurodevelopmental disorders (NDD); Intractable childhood epilepsy with generalized tonic-clonic seizures (ICE-GTC); Malignant migrating partial seizures of infancy (MMPSI); Temporal lobe epilepsy (TLE); Mesial temporal sclerosis (MTS); Neonatal- infantile epilepsy (NIE); Symptomatic focal epilepsy (SFE); Autism spectrum disorders (ASD); Not Reported (NR); Electroencephalography (EEG); Magnetic resonance imaging (MRI).

Abou-Khalil, B., Ge, Q., Desai, R., Ryther, R., Bazyk, A., Bailey, R., et al. (2001). Partial and generalized epilepsy with febrile seizures plus and a novel SCN1A mutation. *Neurology* 57, 2265–2272. doi:10.1212/WNL.57.12.2265.

Afawi, Z., Oliver, K. L., Kivity, S., Mazarib, A., Blatt, I., Neufeld, M. Y., et al. (2016). Multiplex families with epilepsy: Success of clinical and molecular genetic characterization. *Neurology* 86, 713–722. doi:10.1212/WNL.0000000000002404.

Alekov, A. K., Masmudur Rahman, M. D., Mitrovic, N., Lehmann-Horn, F., and Lerche, H. (2001). Enhanced inactivation and acceleration of activation of the sodium channel associated with epilepsy in man. *Eur. J. Neurosci.* 13, 2171–2176. doi:10.1046/j.0953-816X.2001.01590.x.

Alekov, A. K., Rahman, M. M., Mitrovic, N., Lehmannhorn, F., and Lerche, H. (2000). A sodium channel mutation causing epilepsy in man exhibits subtle defects in fast inactivation and activation in vitro. *J. Physiol.* 529, 533–540. doi:10.1111/j.1469-7793.2000.00533.x.

Allen, A. S., Bellows, S. T., Berkovic, S. F., Bridgers, J., Burgess, R., Cavalleri, G., et al. (2017). Ultra-rare genetic variation in common epilepsies: a case-control sequencing study. *Lancet Neurol.* 16, 135–143. doi:10.1016/S1474-4422(16)30359-3.

Allen, A. S., Berkovic, S. F., Cossette, P., Delanty, N., Dlugos, D., Eichler, E. E., et al. (2013). De novo mutations in epileptic encephalopathies. *Nature* 501, 217–221. doi:10.1038/nature12439.

Allen, N. M., Conroy, J., Shahwan, A., Lynch, B., Correa, R. G., Pena, S. D. J., et al. (2016). Unexplained early onset epileptic encephalopathy: Exome screening and phenotype expansion. *Epilepsia* 57, e12–e17. doi:10.1111/epi.13250.

Altarescu, G., Beeri, R., Eldar-Geva, T., Varshaver, I., Margalioth, E. J., Levy-Lahad, E., et al. (2012). PGD for germline mosaicism. *Reprod. Biomed. Online* 25, 390–395. doi:10.1016/j.rbmo.2012.07.003.

Annesi, G., Gambardella, A., Carrideo, S., Incorpora, G., Labate, A., Pasqua, A. A., et al. (2003). Two Novel SCN1A Missense Mutations in Generalized Epilepsy with Febrile Seizures Plus. *Epilepsia* 44, 1257–1258. doi:10.1046/j.1528-1157.2003.22503.x.

Arafat, A., Jing, P., Ma, Y., Pu, M., Nan, G., Fang, H., et al. (2017). Unexplained Early Infantile Epileptic Encephalopathy in Han Chinese Children: Next-Generation Sequencing and Phenotype Enriching. *Sci. Rep.* 7, 46227. doi:10.1038/srep46227.

Arlier, Z., Bayri, Y., Kolb, L. E., Erturk, O., Ozturk, A. K., Bayrakli, F., et al. (2010). Four novel SCN1A mutations in turkish patients with severe myoclonic epilepsy of infancy (SMEI). *J. Child Neurol.* 25, 1265–1268. doi:10.1177/0883073809357241.

Azmanov, D. N., Zhelyazkova, S., Dimova, P. S., Radionova, M., Bojinova, V., Florez, L., et al. (2010). Mosaicism of a missense SCN1A mutation and Dravet syndrome in a Roma/Gypsy family. *Epileptic Disord.* 12, 117–124. doi:10.1684/epd.2010.0311.

Barba, C., Parrini, E., Coras, R., Galuppi, A., Craiu, D., Kluger, G., et al. (2014). Co-occurring malformations of cortical development and SCN1A gene mutations. *Epilepsia* 55, 1009–1019. doi:10.1111/epi.12658.

Barela, A. J., Waddy, S. P., Lickfett, J. G., Hunter, J., Anido, A., Helmers, S. L., et al. (2006). An epilepsy mutation in the sodium channel SCN1A that decreases channel excitability. *J. Neurosci.* 26, 2714–2723. doi:10.1523/JNEUROSCI.2977-05.2006.

Barros, J., Ferreira, A., Brandão, A. F., Lemos, C., Correia, F., Damásio, J., et al. (2014). Familial hemiplegic migraine due to L263V SCN1A mutation: Discordance for epilepsy between two kindreds from Douro Valley. *Cephalalgia* 34, 1015–1020. doi:10.1177/0333102414527015.

Bechi, G., Rusconi, R., Cestèle, S., Striano, P., Franceschetti, S., and Mantegazza, M. (2015). Rescuable folding defective NaV1.1 (SCN1A) mutants in epilepsy: Properties, occurrence, and novel rescuing strategy with peptides targeted to the endoplasmic reticulum. *Neurobiol. Dis.* 75, 100–114. doi:10.1016/j.nbd.2014.12.028.

Bennett, C. A., Petrovski, S., Oliver, K. L., and Berkovic, S. F. (2017). ExACtly zero or once. *Neurol. Genet.* 3, e163. doi:10.1212/NXG.0000000000000163.

Bertelli, S., Barbieri, R., Pusch, M., and Gavazzo, P. (2019). Gain of function of sporadic/familial hemiplegic migraine-causing SCN1A mutations: Use of an optimized cDNA. *Cephalalgia* 39, 477–488. doi:10.1177/0333102418788336.

Binini, N., Sancini, G., Villa, C., Dal Magro, R., Sansoni, V., Rusconi, R., et al. (2017). Identification of two mutations in cis in the SCN1A gene in a family showing genetic epilepsy with febrile seizures plus (GEFS+) and idiopathic generalized epilepsy (IGE). *Brain Res.* 1677, 26–32. doi:10.1016/j.brainres.2017.09.023.

Brunklaus, A., Ellis, R., Stewart, H., Aylett, S., Reavey, E., Jefferson, R., et al. (2015). Homozygous mutations in the SCN1A gene associated with genetic epilepsy with febrile seizures plus and Dravet syndrome in 2 families. *Eur. J. Paediatr. Neurol.* 19, 484–488. doi:10.1016/j.ejpn.2015.02.001.

Buoni, S., Orrico, A., Galli, L., Zannolli, R., Burroni, L., Hayek, J., et al. (2006). SCN1A (2528delG) novel truncating mutation with benign outcome of severe myoclonic epilepsy of infancy. *Neurology* 66, 606–607. doi:10.1212/01.WNL.0000198504.41315.B1.

Butler, K. M., da Silva, C., Alexander, J. J., Hegde, M., and Escayg, A. (2017a). Diagnostic Yield From 339 Epilepsy Patients Screened on a Clinical Gene Panel. *Pediatr. Neurol.* 77, 61–66. doi:10.1016/j.pediatrneurol.2017.09.003.

Butler, K. M., da Silva, C., Shafir, Y., Weisfeld-Adams, J. D., Alexander, J. J., Hegde, M., et al. (2017b). De novo and inherited SCN8A epilepsy mutations detected by gene panel analysis. *Epilepsy Res.* 129, 17–25. doi:10.1016/j.eplepsyres.2016.11.002.

Cantarín Extremera, V., García Peñas, J. J., González Gutiérrez-Solana, L., García Fernández, M., Ruiz-Falcó Rojas, M. L., Duat Rodríguez, A., et al. (2011). Características clínicas, electroencefalográficas y genómicas de los pacientes con epilepsia con crisis febriles plus. *Rev. Neurol.* 52, 404. doi:10.33588/rn.5207.2010693.

Carranza Rojo, D., Hamiwka, L., McMahon, J. M., Dibbens, L. M., Arsov, T., Suls, A., et al. (2011). De novo SCN1A mutations in migrating partial seizures of infancy. *Neurology* 77, 380–383. doi:10.1212/WNL.0b013e318227046d.

Carvill, G. L., Heavin, S. B., Yendle, S. C., McMahon, J. M., O'Roak, B. J., Cook, J., et al. (2013). Targeted resequencing in epileptic encephalopathies identifies de novo mutations in CHD2 and SYNGAP1. *Nat. Genet.* 45, 825–830. doi:10.1038/ng.2646.

Carvill, G. L., Weckhuysen, S., McMahon, J. M., Hartmann, C., Moller, R. S., Hjalgrim, H., et al. (2014). GABRA1 and STXBP1: Novel genetic causes of Dravet syndrome. *Neurology* 82, 1245–1253. doi:10.1212/WNL.0000000000000291.

Castro, M.-J., Stam, A., Lemos, C., de Vries, B., Vanmolkot, K., Barros, J., et al. (2009). First Mutation in the Voltage-Gated Na v 1.1 Subunit Gene SCN1A with Co-Occurring Familial Hemiplegic Migraine and Epilepsy. *Cephalalgia* 29, 308–313. doi:10.1111/j.1468-2982.2008.01721.x.

Catarino, C. B., Liu, J. Y. W., Liagkouras, I., Gibbons, V. S., Labrum, R. W., Ellis, R., et al. (2011). Dravet syndrome as epileptic encephalopathy: evidence from long-term course and neuropathology. *Brain* 134, 2982–3010. doi:10.1093/brain/awr129.

Cestèle, S., Labate, A., Rusconi, R., Tarantino, P., Mumoli, L., Franceschetti, S., et al. (2013).

Divergent effects of the T1174S SCN1A mutation associated with seizures and hemiplegic migraine. *Epilepsia* 54, 927–935. doi:10.1111/epi.12123.

Cetica, V., Chiari, S., Mei, D., Parrini, E., Grisotto, L., Marini, C., et al. (2017). Clinical and genetic factors predicting Dravet syndrome in infants with SCN1A mutations. *Neurology* 88, 1037–1044. doi:10.1212/WNL.0000000000003716.

Ceulemans, B. P. G. M., Claes, L. R. F., and Lagae, L. G. (2004). Clinical correlations of mutations in the SCN1A gene: from febrile seizures to severe myoclonic epilepsy in infancy. *Pediatr. Neurol.* 30, 236–243. doi:10.1016/j.pediatrneurol.2003.10.012.

Chastan, N., Lebas, A., Legoff, F., Parain, D., and Guyant-Marechal, L. (2016). Clinical and electroencephalographic abnormalities during the full duration of a sporadic hemiplegic migraine attack. *Neurophysiol. Clin. Neurophysiol.* 46, 307–311. doi:10.1016/j.neucli.2016.03.004.

Cherepanova, N. S., Leslie, E., Ferguson, P. J., Bamshad, M. J., and Bassuk, A. G. (2013). Presence of Epilepsy-Associated Variants in Large Exome Databases. *J. Neurogenet.* 27, 1–4. doi:10.3109/01677063.2013.772176.

Cho, M. J., Kwon, S. S., Ko, A., Lee, S.-T., Lee, Y. M., Kim, H. D., et al. (2018). Efficacy of Stiripentol in Dravet Syndrome with or without SCN1A Mutations. *J. Clin. Neurol.* 14, 22. doi:10.3988/jcn.2018.14.1.22.

Choi, K. D., Kim, J. S., Kim, H. J., Jung, I., Jeong, S. H., Lee, S. H., et al. (2017). Genetic Variants Associated with Episodic Ataxia in Korea. *Sci. Rep.* 7, 13855. doi:10.1038/s41598-017-14254-7.

Claes, L., Ceulemans, B., Audenaert, D., Smets, K., Löfgren, A., Del-Favero, J., et al. (2003). De novo SCN1A mutations are a major cause of severe myoclonic epilepsy of infancy. *Hum. Mutat.* 21, 615–621. doi:10.1002/humu.10217.

Claes, L., Del-Favero, J., Ceulemans, B., Lagae, L., Van Broeckhoven, C., and De Jonghe, P. (2001). De novo mutations in the sodium-channel gene SCN1A cause severe myoclonic epilepsy of infancy. *Am. J. Hum. Genet.* 68, 1327–1332. doi:10.1086/320609.

Claes, L. R. F., Deprez, L., Suls, A., Baets, J., Smets, K., Van Dyck, T., et al. (2009). The SCN1A variant database: a novel research and diagnostic tool. *Hum. Mutat.* 30, E904–E920. doi:10.1002/humu.21083.

Coll, M., Striano, P., Ferrer-Costa, C., Campuzano, O., Matés, J., del Olmo, B., et al. (2017). Targeted next-generation sequencing provides novel clues for associated epilepsy and cardiac conduction disorder/SUDEP. *PLoS One* 12, e0189618. doi:10.1371/journal.pone.0189618.

Colosimo, E., Gambardella, A., Mantegazza, M., Labate, A., Rusconi, R., Schiavon, E., et al. (2007). Electroclinical Features of a Family with Simple Febrile Seizures and Temporal Lobe Epilepsy Associated with SCN1A Loss-of-Function Mutation. *Epilepsia* 48, 1691–1696. doi:10.1111/j.1528-1167.2007.01153.x.

Combi, R., Grioni, D., Contri, M., Redaelli, S., Redaelli, F., Bassi, M. T., et al. (2009). Clinical and

genetic familial study of a large cohort of Italian children with idiopathic epilepsy. *Brain Res. Bull.* 79, 89–96. doi:10.1016/j.brainresbull.2009.01.008.

Cossette, P., Loukas, A., Lafrenière, R. G., Rochefort, D., Harvey-Girard, E., Ragsdale, D. S., et al. (2003). Functional characterization of the D188V mutation in neuronal voltage-gated sodium channel causing generalized epilepsy with febrile seizures plus (GEFS). *Epilepsy Res.* 53, 107–117. doi:10.1016/S0920-1211(02)00259-0.

Craig, A. K., de Menezes, M. S., and Saneto, R. P. (2012). Dravet syndrome: Patients with co-morbid SCN1A gene mutations and mitochondrial electron transport chain defects. *Seizure* 21, 17–20. doi:10.1016/j.seizure.2011.08.010.

Cui, X., Zeng, F., Liu, Y., Zhang, J., Archacki, S., Zhan, T., et al. (2011). A novel SCN1A missense mutation causes generalized epilepsy with febrile seizures plus in a Chinese family. *Neurosci. Lett.* 503, 27–30. doi:10.1016/j.neulet.2011.08.001.

D'Gama, A. M., Pochareddy, S., Li, M., Jamuar, S. S., Reiff, R. E., Lam, A.-T. N., et al. (2015). Targeted DNA Sequencing from Autism Spectrum Disorder Brains Implicates Multiple Genetic Mechanisms. *Neuron* 88, 910–917. doi:10.1016/j.neuron.2015.11.009.

Daoud, H., Luco, S. M., Li, R., Bareke, E., Beaulieu, C., Jarinova, O., et al. (2016). Next-generation sequencing for diagnosis of Rare diseases in the neonatal intensive care unit. *Cmaj* 188, E254–E260. doi:10.1503/cmaj.150823.

de Kovel, C. G. F., Brilstra, E. H., Van Kempen, M. J. A., Van't Slot, R., Nijman, I. J., Afawi, Z., et al. (2016). Targeted sequencing of 351 candidate genes for epileptic encephalopathy in a large cohort of patients. *Mol. Genet. Genomic Med.* 4, 568–580. doi:10.1002/mgg3.235.

de Lange, I. M., Koudijs, M. J., van 't Slot, R., Gunning, B., Sonsma, A. C. M., van Gemert, L. J. J. M., et al. (2018). Mosaicism of de novo pathogenic SCN1A variants in epilepsy is a frequent phenomenon that correlates with variable phenotypes. *Epilepsia* 59, 690–703. doi:10.1111/epi.14021.

Depienne, C., Trouillard, O., Gourfinkel-An, I., Saint-Martin, C., Bouteiller, D., Graber, D., et al. (2010). Mechanisms for variable expressivity of inherited SCN1A mutations causing Dravet syndrome. *J. Med. Genet.* 47, 404–410. doi:10.1136/jmg.2009.074328.

Depienne, C., Trouillard, O., Saint-Martin, C., Gourfinkel-An, I., Bouteiller, D., Carpentier, W., et al. (2008). Spectrum of SCN1A gene mutations associated with Dravet syndrome: analysis of 333 patients. *J. Med. Genet.* 46, 183–191. doi:10.1136/jmg.2008.062323.

Dhamija, R., Erickson, M. K., St Louis, E. K., Wirrell, E., and Kotagal, S. (2014). Sleep Abnormalities in Children With Dravet Syndrome. *Pediatr. Neurol.* 50, 474–478. doi:10.1016/j.pediatrneurol.2014.01.017.

Dhifallah, S., Lancaster, E., Merrill, S., Leroudier, N., Mantegazza, M., and Cestèle, S. (2018). Gain of function for the SCN1A/HNAv1.1-L1670W mutation responsible for familial hemiplegic migraine. *Front. Mol. Neurosci.* 11, 1–14. doi:10.3389/fnmol.2018.00232.

Dichgans, M., Freilinger, T., Eckstein, G., Babini, E., Lorenz-Depiereux, B., Biskup, S., et al. (2005). Mutation in the neuronal voltage-gated sodium channel SCN1A in familial hemiplegic migraine. *Lancet* 366, 371–377. doi:10.1016/S0140-6736(05)66786-4.

Dimova, P. S., Yordanova, I., Bojinova, V., Jordanova, A., and Kremenski, I. (2010). Generalized Epilepsy With Febrile Seizures plus: Novel SCN1A Mutation. *Pediatr. Neurol.* 42, 137–140. doi:10.1016/j.pediatrneurol.2009.09.007.

Djémié, T., Weckhuysen, S., von Spiczak, S., Carvill, G. L., Jaehn, J., Anttonen, A.-K., et al. (2016). Pitfalls in genetic testing: the story of missed SCN1A mutations. *Mol. Genet. Genomic Med.* 4, 457–464. doi:10.1002/mgg3.217.

Dlugos, D. J., Ferraro, T. N., and Buono, R. J. (2007). Novel De Novo Mutation of a Conserved SCN1A Amino-Acid Residue (R1596). *Pediatr. Neurol.* 37, 303–305. doi:10.1016/j.pediatrneurol.2007.06.008.

Do, T. T. H., Vu, D. M., Huynh, T. T. K., Le, T. K. Van, Sohn, E.-H., Le, T. M. T., et al. (2017). SCN1A Gene Mutation and Adaptive Functioning in 18 Vietnamese Children with Dravet Syndrome. *J. Clin. Neurol.* 13, 62. doi:10.3988/jcn.2017.13.1.62.

Doccini, S., Meschini, M. C., Mei, D., Guerrini, R., Sicca, F., and Santorelli, F. M. (2015). Mitochondrial respiratory chain defects in skin fibroblasts from patients with Dravet syndrome. *Neurol. Sci.* 36, 2151–2155. doi:10.1007/s10072-015-2324-9.

Ebach, K., Joos, H., Doose, H., Stephani, U., Kurlemann, G., Fiedler, B., et al. (2005). SCN1A mutation analysis in myoclonic astatic epilepsy and severe idiopathic generalized epilepsy of infancy with generalized tonic-clonic seizures. *Neuropediatrics* 36, 210–213. doi:10.1055/s-2005-865607.

Ebrahimi, A., Houshmand, M., Tonekaboni, S. H., Fallah Mahboob Passand, M. S., Zainali, S., and Moghadasi, M. (2010). Two Novel Mutations in SCN1A Gene in Iranian Patients with Epilepsy. *Arch. Med. Res.* 41, 207–214. doi:10.1016/j.arcmed.2010.04.007.

Ekici, B., Dilber, C., Aydinli, N., Özmen, M., Tatli, B., and Çaliskan, M. (2011). Severe myoclonic epilepsy of infancy (Dravet syndrome): Clinical and genetic features of nine Turkish patients. *Ann. Indian Acad. Neurol.* 14, 178. doi:10.4103/0972-2327.85879.

Escayg, A., Heils, A., MacDonald, B. T., Haug, K., Sander, T., and Meisler, M. H. (2001). A Novel SCN1A Mutation Associated with Generalized Epilepsy with Febrile Seizures Plus—and Prevalence of Variants in Patients with Epilepsy. *Am. J. Hum. Genet.* 68, 866–873. doi:10.1086/319524.

Escayg, A., MacDonald, B. T., Meisler, M. H., Baulac, S., Huberfeld, G., An-Gourfinkel, I., et al. (2000). Mutations of SCN1A, encoding a neuronal sodium channel, in two families with GEFS+2. *Nat. Genet.* 24, 343–345. doi:10.1038/74159.

Esterhuizen, A. I., Mefford, H. C., Ramesar, R. S., Wang, S., Carvill, G. L., and Wilmshurst, J. M. (2018). Dravet syndrome in South African infants: Tools for an early diagnosis. *Seizure* 62, 99–105. doi:10.1016/j.seizure.2018.09.010.

Fan, C., Wolking, S., Lehmann-Horn, F., Hedrich, U. B. S., Freilinger, T., Lerche, H., et al. (2016). Early-onset familial hemiplegic migraine due to a novel SCN1A mutation. *Cephalalgia* 36, 1238–1247. doi:10.1177/0333102415608360.

Farwell, K. D., Shahmirzadi, L., El-Khechen, D., Powis, Z., Chao, E. C., Tippin Davis, B., et al. (2015). Enhanced utility of family-centered diagnostic exome sequencing with inheritance model–based analysis: results from 500 unselected families with undiagnosed genetic conditions. *Genet. Med.* 17, 578–586. doi:10.1038/gim.2014.154.

Fendri-Kriaa, N., Kammoun, F., Rebai, A., Kolsi, D., Hadj Salem, I., Fakhfakh, F., et al. (2009). Genetic screening of two Tunisian families with generalized epilepsy with febrile seizures plus. *Eur. J. Neurol.* 16, 697–704. doi:10.1111/j.1468-1331.2009.02570.x.

Freilich, E. R., Jones, J. M., Gaillard, W. D., Conry, J. A., Tsuchida, T. N., Reyes, C., et al. (2011). Novel SCN1A mutation in a proband with malignant migrating partial seizures of infancy. *Arch. Neurol.* 68, 665–671. doi:10.1001/archneurol.2011.98.

Fry, A. E., Rees, E., Thompson, R., Mantripragada, K., Blake, P., Jones, G., et al. (2016). Pathogenic copy number variants and SCN1A mutations in patients with intellectual disability and childhood-onset epilepsy. *BMC Med. Genet.* 17, 34. doi:10.1186/s12881-016-0294-2.

Fujiwara, T. (2003). Mutations of sodium channel alpha subunit type 1 (SCN1A) in intractable childhood epilepsies with frequent generalized tonic-clonic seizures. *Brain* 126, 531–546. doi:10.1093/brain/awg053.

Fukuma, G., Oguni, H., Shirasaka, Y., Watanabe, K., Miyajima, T., Yasumoto, S., et al. (2004). Mutations of Neuronal Voltage-gated Na+ Channel alpha1 Subunit Gene SCN1A in Core Severe Myoclonic Epilepsy in Infancy (SMEI) and in Borderline SMEI (SMEB). *Epilepsia* 45, 140–148. doi:10.1111/j.0013-9580.2004.15103.x.

Gaily, E., Anttonen, A.-K., Valanne, L., Liukkonen, E., Träskelin, A.-L., Polvi, A., et al. (2013). Dravet syndrome: New potential genetic modifiers, imaging abnormalities, and ictal findings. *Epilepsia* 54, 1577–1585. doi:10.1111/epi.12256.

Gargus, J. J., and Tournay, A. (2007). Novel Mutation Confirms Seizure Locus SCN1A is Also Familial Hemiplegic Migraine Locus FHM3. *Pediatr. Neurol.* 37, 407–410. doi:10.1016/j.pediatrneurol.2007.06.016.

Gauthier, A. C., Manganas, L. N., and Mattson, R. H. (2017). A novel inherited SCN1A mutation associated with GEFS+ in benign and encephalopathic epilepsy. *J. Clin. Neurosci.* 40, 82–84. doi:10.1016/j.jocn.2017.02.011.

George, A. L. (2004). Molecular basis of inherited epilepsy. *Arch. Neurol.* 61, 473–478. doi:10.1001/archneur.61.4.473.

Goeggel Simonetti, B., Rieubland, C., Courage, C., Strozzi, S., Tschumi, S., Gallati, S., et al. (2012). Duplication of the sodium channel gene cluster on 2q24 in children with early onset epilepsy. *Epilepsia* 53, 2128–2134. doi:10.1111/j.1528-1167.2012.03676.x.

Gökben, S., Berdeli, A., and Serdarolu, G. (2009). An inherited nonsense R1645X mutation in neuronal sodium channel 1-subunit gene in a Turkish patient with severe myoclonic epilepsy of infancy. *Neuropediatrics* 40, 82–84. doi:10.1055/s-0029-1234083.

Gokben, S., Onay, H., Yilmaz, S., Atik, T., Serdaroglu, G., Tekin, H., et al. (2017). Targeted next generation sequencing: the diagnostic value in early-onset epileptic encephalopathy. *Acta Neurol. Belg.* 117, 131–138. doi:10.1007/s13760-016-0709-z.

Goldberg-Stern, H., Aharoni, S., Afawi, Z., Bennett, O., Appenzeller, S., Pendziwiat, M., et al. (2014). Broad Phenotypic Heterogeneity due to a Novel SCN1A Mutation in a Family With Genetic Epilepsy With Febrile Seizures Plus. *J. Child Neurol.* 29, 221–226. doi:10.1177/0883073813509016.

Grosso, S., Orrico, A., Galli, L., Di Bartolo, R., Sorrentino, V., and Balestri, P. (2007). SCN1A mutation associated with atypical panayiotopoulos syndrome. *Neurology* 69, 609–611. doi:10.1212/01.wnl.0000266666.10404.53.

Haginoya, K., Togashi, N., Kaneta, T., Hino-Fukuyo, N., Ishitobi, M., Kakisaka, Y., et al. (2018). [18F]fluorodeoxyglucose-positron emission tomography study of genetically confirmed patients with Dravet syndrome. *Epilepsy Res.* 147, 9–14. doi:10.1016/j.eplepsyres.2018.08.008.

Halvorsen, M., Petrovski, S., Shellhaas, R., Tang, Y., Crandall, L., Goldstein, D., et al. (2016). Mosaic mutations in early-onset genetic diseases. *Genet. Med.* 18, 746–749. doi:10.1038/gim.2015.155.

Hamdan, F. F., Myers, C. T., Cossette, P., Lemay, P., Spiegelman, D., Laporte, A. D., et al. (2017). High Rate of Recurrent De Novo Mutations in Developmental and Epileptic Encephalopathies. *Am. J. Hum. Genet.* 101, 664–685. doi:10.1016/j.ajhg.2017.09.008.

Harkin, L. A., McMahon, J. M., Iona, X., Dibbens, L., Pelekanos, J. T., Zuberi, S. M., et al. (2007). The spectrum of SCN1A-related infantile epileptic encephalopathies. *Brain* 130, 843–852. doi:10.1093/brain/awm002.

Hattori, J., Ouchida, M., Ono, J., Miyake, S., Maniwa, S., Mimaki, N., et al. (2008). A Screening test for the prediction of Dravet syndrome before one year of age. *Epilepsia* 49, 626–633. doi:10.1111/j.1528-1167.2007.01475.x.

Helbig, K. L., Farwell Hagman, K. D., Shinde, D. N., Mroske, C., Powis, Z., Li, S., et al. (2016). Diagnostic exome sequencing provides a molecular diagnosis for a significant proportion of patients with epilepsy. *Genet. Med.* 18, 898–905. doi:10.1038/gim.2015.186.

Herini, E. S., Gunadi, van Kempen, M. J. A., Yusoff, S., Sutaryo, Sunartini, et al. (2010). Novel SCN1A mutations in Indonesian patients with severe myoclonic epilepsy in infancy. *Pediatr. Int.* 52, 234–239. doi:10.1111/j.1442-200X.2009.02916.x.

Hernández Chávez, M., Mesa Latorre, T., Pedraza Herrera, M., and Troncoso Schifferli, M. (2014). ¿Crisis febriles complejas o síndrome de Dravet?: Descripción de 3 casos clínicos. *Rev. Chil. pediatría* 85, 588–593. doi:10.4067/S0370-41062014000500010.

Hesse, A. N., Bevilacqua, J., Shankar, K., and Reddi, H. V. (2018). Retrospective genotype-

phenotype analysis in a 305 patient cohort referred for testing of a targeted epilepsy panel. *Epilepsy Res.* 144, 53–61. doi:10.1016/j.eplepsyres.2018.05.004.

Hildebrand, M. S., Myers, C. T., Carvill, G. L., Regan, B. M., Damiano, J. A., Mullen, S. A., et al. (2016). A targeted resequencing gene panel for focal epilepsy. *Neurology* 86, 1605–1612. doi:10.1212/WNL.0000000000002608.

Hindocha, N., Nashef, L., Elmslie, F., Birch, R., Zuberi, S., Al-Chalabi, A., et al. (2008). Two cases of sudden unexpected death in epilepsy in a GEFS+ family with an SCN1A mutation. *Epilepsia* 49, 360–365. doi:10.1111/j.1528-1167.2007.01439_2.x.

Hoffman-Zacharska, D., Szczepanik, E., Terczynska, I., Goszczanska-Ciuchta, A., Zalewska-Miszkurka, Z., Tataj, R., et al. (2015). From focal epilepsy to dravet syndrome –heterogeneity of the phenotype due to SCN1A mutations of the p.Arg1596 amino acid residue in the nav1.1 subunit. *Neurol. Neurochir. Pol.* 49, 258–266. doi:10.1016/j.pjnns.2015.06.006.

Huang, A. Y., Xu, X., Ye, A. Y., Wu, Q., Yan, L., Zhao, B., et al. (2014). Postzygotic single-nucleotide mosaicisms in whole-genome sequences of clinically unremarkable individuals. *Cell Res.* 24, 1311–1327. doi:10.1038/cr.2014.131.

Iannetti, P., Parisi, P., Spalice, A., Ruggieri, M., and Zara, F. (2009). Addition of verapamil in the treatment of severe myoclonic epilepsy in infancy. *Epilepsy Res.* 85, 89–95. doi:10.1016/j.eplepsyres.2009.02.014.

Ishii, A., Watkins, J. C., Chen, D., Hirose, S., and Hammer, M. F. (2017). Clinical implications of SCN1A missense and truncation variants in a large Japanese cohort with Dravet syndrome. *Epilepsia* 58, 282–290. doi:10.1111/epi.13639.

Jiao, J., Yang, Y., Shi, Y., Chen, J., Gao, R., Fan, Y., et al. (2013). Modeling Dravet syndrome using induced pluripotent stem cells (iPSCs) and directly converted neurons. *Hum. Mol. Genet.* 22, 4241–4252. doi:10.1093/hmg/ddt275.

Jiménez-Arredondo, R. E., Brambila-Tapia, A. J. L., Mercado-Silva, F. M., Magaña-Torres, M. T., and Figuera, L. E. (2017). Determination of SCN1A genetic variants in Mexican patients with refractory epilepsy and Dravet syndrome. *Genet. Mol. Res.* 16, 1–5. doi:10.4238/gmr16029405.

Jingami, N., Matsumoto, R., Ito, H., Ishii, A., Ihara, Y., Hirose, S., et al. (2014). A novel SCN1A mutation in a cytoplasmic loop in intractable juvenile myoclonic epilepsy without febrile seizures. *Epileptic Disord.* 16, 227–231. doi:10.1684/epd.2014.0657.

Kahlig, K. M., Rhodes, T. H., Pusch, M., Freilinger, T., Pereira-Monteiro, J. M., Ferrari, M. D., et al. (2008). Divergent sodium channel defects in familial hemiplegic migraine. *Proc. Natl. Acad. Sci. U. S. A.* 105, 9799–9804. doi:10.1073/pnas.0711717105.

Kearney, J. A., Yang, Y., Beyer, B., Bergren, S. K., Claes, L., DeJonghe, P., et al. (2006). Severe epilepsy resulting from genetic interaction between Scn2a and Kcnq2. *Hum. Mol. Genet.* 15, 1043–1048. doi:10.1093/hmg/ddl019.

Kim, D. W., Lim, B. C., Kim, K. J., Chae, J. H., Lee, R., and Lee, S. K. (2013). Low incidence of

SCN1A genetic mutation in patients with hemiconvulsion–hemiplegia–epilepsy syndrome. *Epilepsy Res.* 106, 440–445. doi:10.1016/j.eplepsyres.2013.06.012.

Kim, J., and Lee, C. G. (2017). Coinheritance of Novel Mutations in SCN1A Causing GEFS+ and in KDM6A Causing Kabuki Syndrome in a Family. *Ann. Clin. Lab. Sci.* 47, 229–235. Available at: http://www.ncbi.nlm.nih.gov/pubmed/28442529.

Kim, Y. O., Bellows, S., Mcmahon, J. M., Iona, X., Damiano, J., Dibbens, L., et al. (2014). Atypical multifocal Dravet syndrome lacks generalized seizures and may show later cognitive decline. *Dev. Med. Child Neurol.* 56, 85–90. doi:10.1111/dmcn.12322.

Kimura, K., Sugawara, T., Mazaki-Miyazaki, E., Hoshino, K., Nomura, Y., Tateno, A., et al. (2005). A missense mutation in SCN1A in brothers with severe myoclonic epilepsy in infancy (SMEI) inherited from a father with febrile seizures. *Brain Dev.* 27, 424–430. doi:10.1016/j.braindev.2004.11.005.

Kivity, S., Oliver, K. L., Afawi, Z., Damiano, J. A., Arsov, T., Bahlo, M., et al. (2017). SCN1A clinical spectrum includes the self-limited focal epilepsies of childhood. *Epilepsy Res.* 131, 9–14. doi:10.1016/j.eplepsyres.2017.01.012.

Klassen, T., Davis, C., Goldman, A., Burgess, D., Chen, T., Wheeler, D., et al. (2011). Exome Sequencing of Ion Channel Genes Reveals Complex Profiles Confounding Personal Risk Assessment in Epilepsy. *Cell* 145, 1036–1048. doi:10.1016/j.cell.2011.05.025.

Ko, A., Youn, S. E., Kim, S. H., Lee, J. S., Kim, S., Choi, J. R., et al. (2018). Targeted gene panel and genotype-phenotype correlation in children with developmental and epileptic encephalopathy. *Epilepsy Res.* 141, 48–55. doi:10.1016/j.eplepsyres.2018.02.003.

Kodera, H., Kato, M., Nord, A. S., Walsh, T., Lee, M., Yamanaka, G., et al. (2013). Targeted capture and sequencing for detection of mutations causing early onset epileptic encephalopathy. *Epilepsia* 54, 1262–1269. doi:10.1111/epi.12203.

Kothur, K., Holman, K., Farnsworth, E., Ho, G., Lorentzos, M., Troedson, C., et al. (2018). Diagnostic yield of targeted massively parallel sequencing in children with epileptic encephalopathy. *Seizure* 59, 132–140. doi:10.1016/j.seizure.2018.05.005.

Kumar, A., Juhász, C., Luat, A., Govil-Dalela, T., Behen, M. E., Hicks, M. A., et al. (2018). Evolution of Brain Glucose Metabolic Abnormalities in Children With Epilepsy and SCN1A Gene Variants. *J. Child Neurol.* 33, 832–836. doi:10.1177/0883073818796373.

Kwong, A. K.-Y., Ho, A. C.-C., Fung, C.-W., and Wong, V. C.-N. (2015). Analysis of Mutations in 7 Genes Associated with Neuronal Excitability and Synaptic Transmission in a Cohort of Children with Non-Syndromic Infantile Epileptic Encephalopathy. *PLoS One* 10, e0126446. doi:10.1371/journal.pone.0126446.

Kwong, A. K. Y., Fung, C. W., Chan, S. Y., and Wong, V. C. N. (2012). Identification of SCN1A and PCDH19 mutations in Chinese children with Dravet syndrome. *PLoS One* 7, e41802. doi:10.1371/journal.pone.0041802.

Lal, D., Reinthaler, E. M., Dejanovic, B., May, P., Thiele, H., Lehesjoki, A.-E., et al. (2016).

Evaluation of Presumably Disease Causing SCN1A Variants in a Cohort of Common Epilepsy Syndromes. *PLoS One* 11, e0150426. doi:10.1371/journal.pone.0150426.

Lam, C. W., Law, C. Y., Leung, K. F., Lai, C. K., Pak-lam Chen, S., Chan, B., et al. (2015). NMR-based urinalysis for rapid diagnosis of β-ureidopropionase deficiency in a patient with Dravet syndrome. *Clin. Chim. Acta* 440, 201–204. doi:10.1016/j.cca.2014.10.030.

Langer, S., Geigl, J. B., Wagenstaller, J., Lederer, G., Hempel, M., Daumer-Haas, C., et al. (2006). Delineation of a 2q deletion in a girl with dysmorphic features and epilepsy. *Am. J. Med. Genet. Part A* 140A, 764–768. doi:10.1002/ajmg.a.31141.

Le Gal, F., Korff, C. M., Monso-Hinard, C., Mund, M. T., Morris, M., Malafosse, A., et al. (2010). A case of SUDEP in a patient with Dravet syndrome with SCN1A mutation. *Epilepsia* 51, 1915–1918. doi:10.1111/j.1528-1167.2010.02691.x.

Le Gal, F., Lebon, S., Ramelli, G. P., Datta, A. N., Mercati, D., Maier, O., et al. (2014). When is a child with status epilepticus likely to have Dravet syndrome? *Epilepsy Res.* 108, 740–747. doi:10.1016/j.eplepsyres.2014.02.019.

Lee, C. G., Lee, J., and Lee, M. (2018). Multi-gene panel testing in Korean patients with common genetic generalized epilepsy syndromes. *PLoS One* 13, e0199321. doi:10.1371/journal.pone.0199321.

Lee, H.-F., Chi, C.-S., Tsai, C.-R., Chen, C.-H., and Wang, C.-C. (2014). Electroencephalographic features of patients with SCN1A-positive Dravet syndrome. *Brain Dev.* 37, 599–611. doi:10.1016/j.braindev.2014.10.003.

Lee, J. S., Jung, N. Y., Jang, Y. K., Kim, H. J., Seo, S. W., Lee, J., et al. (2017). Prognosis of patients with behavioral variant frontotemporal dementia who have focal versus diffuse frontal atrophy. *J. Clin. Neurol.* 13, 234–242. doi:10.3988/jcn.2017.13.3.234.

Lemke, J. R., Riesch, E., Scheurenbrand, T., Schubach, M., Wilhelm, C., Steiner, I., et al. (2012). Targeted next generation sequencing as a diagnostic tool in epileptic disorders. *Epilepsia* 53, 1387–1398. doi:10.1111/j.1528-1167.2012.03516.x.

Liao, W.-P., Shi, Y.-W., Long, Y.-S., Zeng, Y., Li, T., Yu, M.-J., et al. (2010). Partial epilepsy with antecedent febrile seizures and seizure aggravation by antiepileptic drugs: Associated with loss of function of Nav1.1. *Epilepsia* 51, 1669–1678. doi:10.1111/j.1528-1167.2010.02645.x.

Lim, B. C., Hwang, H., Chae, J. H., Choi, J.-E., Hwang, Y. S., Kang, S.-H., et al. (2011). SCN1A mutational analysis in Korean patients with Dravet syndrome. *Seizure* 20, 789–794. doi:10.1016/j.seizure.2011.08.002.

Lim, B. C., Hwang, H., Kim, H., Chae, J.-H., Choi, J., Kim, K. J., et al. (2015). Epilepsy phenotype associated with a chromosome 2q24.3 deletion involving SCN1A: Migrating partial seizures of infancy or atypical Dravet syndrome? *Epilepsy Res.* 109, 34–39. doi:10.1016/j.eplepsyres.2014.10.008.

Lin, W.-D., Chang, K.-P., Wang, C.-H., Chen, S.-J., Fan, P.-C., Weng, W.-C., et al. (2013).

Molecular aspects of Dravet syndrome patients in Taiwan. *Clin. Chim. Acta* 421, 34–40. doi:10.1016/j.cca.2013.02.015.

Lin, W.-D., Chou, I.-C., and Tsai, F.-J. (2010). Novel human pathological mutations. Gene symbol: SCN1A. Disease: generalized epilepsy with febrile seizures plus. *Hum. Genet.* 127, 478. Available at: http://www.ncbi.nlm.nih.gov/pubmed/21488289.

Lindy, A. S., Stosser, M. B., Butler, E., Downtain-Pickersgill, C., Shanmugham, A., Retterer, K., et al. (2018). Diagnostic outcomes for genetic testing of 70 genes in 8565 patients with epilepsy and neurodevelopmental disorders. *Epilepsia* 59, 1062–1071. doi:10.1111/epi.14074.

Liu, J., Tong, L., Song, S., Niu, Y., Li, J., Wu, X., et al. (2018). Novel and de novo mutations in pediatric refractory epilepsy. *Mol. Brain* 11, 48. doi:10.1186/s13041-018-0392-5.

Liu, Y., Lopez-Santiago, L. F., Yuan, Y., Jones, J. M., Zhang, H., O'Malley, H. A., et al. (2013). Dravet syndrome patient-derived neurons suggest a novel epilepsy mechanism. *Ann. Neurol.* 74, 128–139. doi:10.1002/ana.23897.

Livingston, J. H., Cross, J. H., Mclellan, A., Birch, R., and Zuberi, S. M. (2009). A Novel Inherited Mutation in the Voltage Sensor Region of SCN1A Is Associated With Panayiotopoulos Syndrome in Siblings and Generalized Epilepsy With Febrile Seizures Plus. *J. Child Neurol.* 24, 503–508. doi:10.1177/0883073808324537.

Lossin, C. (2009). A catalog of SCN1A variants. *Brain Dev.* 31, 114–130. doi:10.1016/j.braindev.2008.07.011.

Lossin, C., Rhodes, T. H., Desai, R. R., Vanoye, C. G., Wang, D., Carniciu, S., et al. (2003). Epilepsy-Associated Dysfunction in the Voltage-Gated Neuronal Sodium Channel SCN1A. *J. Neurosci.* 23, 11289–11295. doi:10.1523/jneurosci.23-36-11289.2003.

Ma, Y., Chen, C., Wang, Y., Wu, L., He, F., Chen, C., et al. (2016). Analysis copy number variation of Chinese children in early-onset epileptic encephalopathies with unknown cause. *Clin. Genet.* 90, 428–436. doi:10.1111/cge.12768.

Madia, F., Striano, P., Gennaro, E., Malacarne, M., Paravidino, R., Biancheri, R., et al. (2006). Cryptic chromosome deletions involving SCN1A in severe myoclonic epilepsy of infancy. *Neurology* 67, 1230–1235. doi:10.1212/01.wnl.0000238513.70878.54.

Mahdieh, N., Mikaeeli, S., Badv, R. S., Shirazi, A. G., Maleki, M., and Rabbani, B. (2018). Pathogenic significance of SCN1A splicing variants causing Dravet syndrome: Improving diagnosis with targeted sequencing for variants by in silico analysis. *Clin. Neurol. Neurosurg.* 166, 80–90. doi:10.1016/j.clineuro.2018.01.030.

Mahoney, K., Moore, S. J., Buckley, D., Alam, M., Parfrey, P., Penney, S., et al. (2009). Variable neurologic phenotype in a GEFS+ family with a novel mutation in SCN1A. *Seizure* 18, 492–497. doi:10.1016/j.seizure.2009.04.009.

Mak, C. M., Chan, K. Y. W., Yau, E. K. C., Chen, S. P. L., Siu, W. K., Law, C. Y., et al. (2011). Genetic diagnosis of severe myoclonic epilepsy of infancy (Dravet syndrome) with SCN1A mutations in the Hong Kong Chinese patients. *Hong Kong Med. J. = Xianggang yi xue za zhi*

17, 500–2. Available at: http://www.ncbi.nlm.nih.gov/pubmed/22147323.

Maksemous, N., Smith, R. A., Haupt, L. M., and Griffiths, L. R. (2016). Targeted next generation sequencing identifies novel NOTCH3 gene mutations in CADASIL diagnostics patients. *Hum. Genomics* 10, 38. doi:10.1186/s40246-016-0093-z.

Mantegazza, M., Gambardella, A., Rusconi, R., Schiavon, E., Annesi, F., Cassulini, R. R., et al. (2005). Identification of an Nav1.1 sodium channel (SCN1A) loss-of-function mutation associated with familial simple febrile seizures. *Proc. Natl. Acad. Sci. U. S. A.* 102, 18177–18182. doi:10.1073/pnas.0506818102.

Margherita Mancardi, M., Striano, P., Gennaro, E., Madia, F., Paravidino, R., Scapolan, S., et al. (2006). Familial Occurrence of Febrile Seizures and Epilepsy in Severe Myoclonic Epilepsy of Infancy (SMEI) Patients with SCN1A Mutations. *Epilepsia* 47, 1629–1635. doi:10.1111/j.1528-1167.2006.00641.x.

Marini, C., Mei, D., Temudo, T., Ferrari, A. R., Buti, D., Dravet, C., et al. (2007). Idiopathic Epilepsies with Seizures Precipitated by Fever and SCN1A Abnormalities. *Epilepsia* 48, 1678–1685. doi:10.1111/j.1528-1167.2007.01122.x.

Martin, P., Rautenstrauβ, B., Abicht, A., Fahrbach, J., and Koster, S. (2010). Severe Myoclonic Epilepsy in Infancy – Adult Phenotype with Bradykinesia, Hypomimia, and Perseverative Behavior: Report of Five Cases. *Mol. Syndromol.* 1, 231–238. doi:10.1159/000326746.

McArdle, E. J., Kunic, J. D., and George, A. L. (2008). Novel SCN1A frameshift mutation with absence of truncated Na V 1.1 protein in severe myoclonic epilepsy of infancy. *Am. J. Med. Genet. Part A* 146A, 2421–2423. doi:10.1002/ajmg.a.32448.

Mercimek-Mahmutoglu, S., Patel, J., Cordeiro, D., Hewson, S., Callen, D., Donner, E. J., et al. (2015). Diagnostic yield of genetic testing in epileptic encephalopathy in childhood. *Epilepsia* 56, 707–716. doi:10.1111/epi.12954.

Mhanni, A. A., Hartley, J. N., Sanger, W. G., Chudley, A. E., and Spriggs, E. L. (2011). Variable expressivity of a novel mutation in the SCN1A gene leading to an autosomal dominant seizure disorder. *Seizure* 20, 711–712. doi:10.1016/j.seizure.2011.06.014.

Mina, E. Della, Ciccone, R., Brustia, F., Bayindir, B., Limongelli, I., Vetro, A., et al. (2015). Improving molecular diagnosis in epilepsy by a dedicated high-throughput sequencing platform. *Eur. J. Hum. Genet.* 23, 354–362. doi:10.1038/ejhg.2014.92.

Miyama, S., Goto, T., Inoue, Y., and Yamakawa, K. (2008). Monozygotic Twins With Severe Myoclonic Epilepsy in Infancy Discordant for Clinical Features. *Pediatr. Neurol.* 39, 120–122. doi:10.1016/j.pediatrneurol.2008.04.003.

Moehring, J., Von Spiczak, S., Moeller, F., Helbig, I., Wolff, S., Jansen, O., et al. (2013). Variability of EEG-fMRI findings in patients with SCN1A -positive Dravet syndrome. *Epilepsia* 54, 918–926. doi:10.1111/epi.12119.

Møller, R. S., Larsen, L. H. G., Johannesen, K. M., Talvik, I., Talvik, T., Vaher, U., et al. (2016).

Gene panel testing in epileptic encephalopathies and familial epilepsies. *Mol. Syndromol.* 7, 210–219. doi:10.1159/000448369.

Møller, R. S., Schneider, L. M., Hansen, C. P., Bugge, M., Ullmann, R., Tommerup, N., et al. (2008). Balanced translocation in a patient with severe myoclonic epilepsy of infancy disrupts the sodium channel gene SCN1A. *Epilepsia* 49, 1091–1094. doi:10.1111/j.1528-1167.2008.01550.x.

Morimoto, M., Mazaki, E., Nishimura, A., Chiyonobu, T., Sawai, Y., Murakami, A., et al. (2006). SCN1A Mutation Mosaicism in a Family with Severe Myoclonic Epilepsy in Infancy. *Epilepsia* 47, 1732–1736. doi:10.1111/j.1528-1167.2006.00645.x.

Mulley, J. C., Hodgson, B., McMahon, J. M., Iona, X., Bellows, S., Mullen, S. A., et al. (2013). Role of the sodium channel SCN9A in genetic epilepsy with febrile seizures plus and Dravet syndrome. *Epilepsia* 54, e122–e126. doi:10.1111/epi.12323.

Muona, M., Berkovic, S. F., Dibbens, L. M., Oliver, K. L., Maljevic, S., Bayly, M. A., et al. (2015). A recurrent de novo mutation in KCNC1 causes progressive myoclonus epilepsy. *Nat. Genet.* 47, 39–46. doi:10.1038/ng.3144.

Myers, K. A., Burgess, R., Afawi, Z., Damiano, J. A., Berkovic, S. F., Hildebrand, M. S., et al. (2017a). De novo SCN1A pathogenic variants in the GEFS+ spectrum: Not always a familial syndrome. *Epilepsia* 58, e26–e30. doi:10.1111/epi.13649.

Myers, K. A., McMahon, J. M., Mandelstam, S. A., Mackay, M. T., Kalnins, R. M., Leventer, R. J., et al. (2017b). Fatal Cerebral Edema With Status Epilepticus in Children With Dravet Syndrome: Report of 5 Cases. *Pediatrics* 139, e20161933. doi:10.1542/peds.2016-1933.

Nabbout, R., Gennaro, E., Dalla Bernardina, B., Dulac, O., Madia, F., Bertini, E., et al. (2003). Spectrum of SCN1A mutations in severe myoclonic epilepsy of infancy. *Neurology* 60, 1961–1967. doi:10.1212/01.WNL.0000069463.41870.2F.

Nagao, Y., Mazaki-Miyazaki, E., Okamura, N., Takagi, M., Igarashi, T., and Yamakawa, K. (2005). A family of generalized epilepsy with febrile seizures plus type 2—a new missense mutation of SCN1A found in the pedigree of several patients with complex febrile seizures. *Epilepsy Res.* 63, 151–156. doi:10.1016/j.eplepsyres.2004.11.005.

Nakayama, T., Ogiwara, I., Ito, K., Kaneda, M., Mazaki, E., Osaka, H., et al. (2010). Deletions of SCN1A 5′ genomic region with promoter activity in Dravet syndrome. *Hum. Mutat.* 31, 820–829. doi:10.1002/humu.21275.

Nicholl, J., Waters, W., Suwalski, S., Brown, S., Hull, Y., Harbord, M. G., et al. (2013). Epilepsy with cognitive deficit and autism spectrum disorders: Prospective diagnosis by array CGH. *Am. J. Med. Genet. Part B Neuropsychiatr. Genet.* 162, 24–35. doi:10.1002/ajmg.b.32114.

Nicita, F., Spalice, A., Papetti, L., Ursitti, F., Parisi, P., Gennaro, E., et al. (2010). Genotype-phenotype correlations in a group of 15 SCN1A-mutated italian patients with GEFS+ spectrum (seizures plus, classical and borderline severe myoclonic epilepsy of infancy). *J. Child Neurol.* 25, 1369–1376. doi:10.1177/0883073810365737.

Nishri, D., Blumkin, L., Lev, D., Leshinsky-Silver, E., Abu-Rashid, M., Birch, R., et al. (2010). Hepatic coma culminating in severe brain damage in a child with a SCN1A mutation. *Eur. J. Paediatr. Neurol.* 14, 456–459. doi:10.1016/j.ejpn.2010.03.002.

O'Roak, B. J., Deriziotis, P., Lee, C., Vives, L., Schwartz, J. J., Girirajan, S., et al. (2011). Exome sequencing in sporadic autism spectrum disorders identifies severe de novo mutations. *Nat. Genet.* 43, 585–589. doi:10.1038/ng.835.

Oates, S., Tang, S., Rosch, R., Lear, R., Hughes, E. F., Williams, R. E., et al. (2018). Incorporating epilepsy genetics into clinical practice: A 360°evaluation. *npj Genomic Med.* 3, 1–11. doi:10.1038/s41525-018-0052-9.

Ohashi, T., Akasaka, N., Kobayashi, Y., Magara, S., Kawashima, H., Matsumoto, N., et al. (2014). Infantile epileptic encephalopathy with a hyperkinetic movement disorder and hand stereotypies associated with a novel SCN1A mutation. *Epileptic Disord.* 16, 208–212. doi:10.1684/epd.2014.0649.

Ohmori, I., Kahlig, K. M., Rhodes, T. H., Wang, D. W., and George, A. L. (2006). Nonfunctional SCN1A Is Common in Severe Myoclonic Epilepsy of Infancy. *Epilepsia* 47, 1636–1642. doi:10.1111/j.1528-1167.2006.00643.x.

Ohmori, I., Ouchida, M., Kobayashi, K., Jitsumori, Y., Inoue, T., Shimizu, K., et al. (2008). Rasmussen encephalitis associated with SCN1A mutation. *Epilepsia* 49, 521–526. doi:10.1111/j.1528-1167.2007.01411.x.

Ohmori, I., Ouchida, M., Ohtsuka, Y., Oka, E., and Shimizu, K. (2002). Significant correlation of the SCN1A mutations and severe myoclonic epilepsy in infancy. *Biochem. Biophys. Res. Commun.* 295, 17–23. doi:10.1016/S0006-291X(02)00617-4.

Okumura, A., Uematsu, M., Imataka, G., Tanaka, M., Okanishi, T., Kubota, T., et al. (2012). Acute encephalopathy in children with Dravet syndrome. *Epilepsia* 53, 79–86. doi:10.1111/j.1528-1167.2011.03311.x.

Okumura, A., Yamamoto, T., Shimojima, K., Honda, Y., Abe, S., Ikeno, M., et al. (2011). Refractory neonatal epilepsy with a de novo duplication of chromosome 2q24.2q24.3. *Epilepsia* 52, e66–e69. doi:10.1111/j.1528-1167.2011.03139.x.

Orrico, A., Galli, L., Grosso, S., Buoni, S., Pianigiani, R., Balestri, P., et al. (2009). Mutational analysis of the SCN1A, SCN1B and GABRG2 genes in 150 Italian patients with idiopathic childhood epilepsies. *Clin. Genet.* 75, 579–581. doi:10.1111/j.1399-0004.2009.01155.x.

Osaka, H., Ogiwara, I., Mazaki, E., Okamura, N., Yamashita, S., Iai, M., et al. (2007). Patients with a sodium channel alpha 1 gene mutation show wide phenotypic variation. *Epilepsy Res.* 75, 46–51. doi:10.1016/j.eplepsyres.2007.03.018.

Ostrander, B. E. P., Butterfield, R. J., Pedersen, B. S., Farrell, A. J., Layer, R. M., Ward, A., et al. (2018). Whole-genome analysis for effective clinical diagnosis and gene discovery in early infantile epileptic encephalopathy. *npj Genomic Med.* 3, 22. doi:10.1038/s41525-018-0061-8.

Ozmen, M., Dilber, C., Tatlı, B., Aydınlı, N., Calışkan, M., and Ekici, B. (2011). Severe myoclonic epilepsy of infancy (Dravet syndrome): Clinical and genetic features of nine Turkish patients. *Ann. Indian Acad. Neurol.* 14, 178–81. doi:10.4103/0972-2327.85879.

Parrini, E., Marini, C., Mei, D., Galuppi, A., Cellini, E., Pucatti, D., et al. (2017). Diagnostic Targeted Resequencing in 349 Patients with Drug-Resistant Pediatric Epilepsies Identifies Causative Mutations in 30 Different Genes. *Hum. Mutat.* 38, 216–225. doi:10.1002/humu.23149.

Pérez Delgado, R., Lafuente Hidalgo, M., López Pisón, J., Sebastián Torres, B., Torres Claveras, S., García Jiménez, M. C., et al. (2010). Epilepsia de inicio entre el mes y los tres meses de vida: nuestra experiencia de 11 años. *Neurología* 25, 90–95. doi:10.1016/S0213-4853(10)70032-3.

Perucca, P., Scheffer, I. E., Harvey, A. S., James, P. A., Lunke, S., Thorne, N., et al. (2017). Real-world utility of whole exome sequencing with targeted gene analysis for focal epilepsy. *Epilepsy Res.* 131, 1–8. doi:10.1016/j.eplepsyres.2017.02.001.

Pescucci, C., Caselli, R., Grosso, S., Mencarelli, M. A., Mari, F., Farnetani, M. A., et al. (2007). 2q24–q31 Deletion: Report of a case and review of the literature. *Eur. J. Med. Genet.* 50, 21–32. doi:10.1016/j.ejmg.2006.09.001.

Peters, C., Rosch, R. E., Hughes, E., and Ruben, P. C. (2016). Temperature-dependent changes in neuronal dynamics in a patient with an SCN1A mutation and hyperthermia induced seizures. *Sci. Rep.* 6, 31879. doi:10.1038/srep31879.

Petrelli, C., Passamonti, C., Cesaroni, E., Mei, D., Guerrini, R., Zamponi, N., et al. (2012). Early clinical features in Dravet syndrome patients with and without SCN1A mutations. *Epilepsy Res.* 99, 21–27. doi:10.1016/j.eplepsyres.2011.10.010.

Pineda-Trujillo, N., Carrizosa, J., Cornejo, W., Arias, W., Franco, C., Cabrera, D., et al. (2005). A novel SCN1A mutation associated with severe GEFS+ in a large South American pedigree. *Seizure* 14, 123–128. doi:10.1016/j.seizure.2004.12.007.

Pippucci, T., Licchetta, L., Baldassari, S., Palombo, F., Menghi, V., D'Aurizio, R., et al. (2015). Epilepsy with auditory features. *Neurol. Genet.* 1, e5. doi:10.1212/NXG.0000000000000005.

Poryo, M., Clasen, O., Oehl-Jaschkowitz, B., Christmann, A., Gortner, L., and Meyer, S. (2017). Dravet syndrome: a new causative SCN1A mutation? *Clin. Case Reports* 5, 613–615. doi:10.1002/ccr3.787.

Posey, J. E., Harel, T., Liu, P., Rosenfeld, J. A., James, R. A., Coban Akdemir, Z. H., et al. (2017). Resolution of Disease Phenotypes Resulting from Multilocus Genomic Variation. *N. Engl. J. Med.* 376, 21–31. doi:10.1056/NEJMoa1516767.

Raymond, G., Wohler, E., Dinsmore, C., Cox, J., Johnston, M., Batista, D., et al. (2011). An interstitial duplication at 2q24.3 involving the SCN1A, SCN2A, SCN3A genes associated with infantile epilepsy. *Am. J. Med. Genet. Part A* 155, 920–923. doi:10.1002/ajmg.a.33929.

Ream, M. A., and Mikati, M. A. (2014). Clinical utility of genetic testing in pediatric drug-resistant epilepsy: A pilot study. *Epilepsy Behav.* 37, 241–248. doi:10.1016/j.yebeh.2014.06.018.

Retterer, K., Juusola, J., Cho, M. T., Vitazka, P., Millan, F., Gibellini, F., et al. (2016). Clinical application of whole-exome sequencing across clinical indications. *Genet. Med.* 18, 696–704. doi:10.1038/gim.2015.148.

Reyes, I. S., Hsieh, D. T., Laux, L. C., and Wilfong, A. A. (2011). Alleged Cases of Vaccine Encephalopathy Rediagnosed Years Later as Dravet Syndrome. *Pediatrics* 128. doi:10.1542/peds.2010-0887.

Rhodes, T. H., Lossin, C., Vanoye, C. G., Wang, D. W., and George, A. L. (2004). Noninactivating voltage-gated sodium channels in severe myoclonic epilepsy of infancy. *Proc. Natl. Acad. Sci. U. S. A.* 101, 11147–11152. doi:10.1073/pnas.0402482101.

Rhodes, T. H., Vanoye, C. G., Ohmori, I., Ogiwara, I., Yamakawa, K., and George, A. L. (2005). Sodium channel dysfunction in intractable childhood epilepsy with generalized tonic-clonic seizures. *J. Physiol.* 569, 433–445. doi:10.1113/jphysiol.2005.094326.

Rilstone, J. J., Coelho, F. M., Minassian, B. A., and Andrade, D. M. (2012). Dravet syndrome: Seizure control and gait in adults with different SCN1A mutations. *Epilepsia* 53, 1421–1428. doi:10.1111/j.1528-1167.2012.03583.x.

Riva, D., Vago, C., Pantaleoni, C., Bulgheroni, S., Mantegazza, M., and Franceschetti, S. (2009). Progressive neurocognitive decline in two children with Dravet syndrome, de novo SCN1A truncations and different epileptic phenotypes. *Am. J. Med. Genet. Part A* 149A, 2339–2345. doi:10.1002/ajmg.a.33029.

Rocca, F. E., De Marco, E. V., Annesi, F., Civitelli, D., Provenzano, G., Sproviero, W., et al. (2010). Novel human pathological mutations. *Hum. Genet.* 127, 463–490. doi:10.1007/s00439-010-0788-5.

Rodda, J. M., Scheffer, I. E., McMahon, J. M., Berkovic, S. F., and Graham, H. K. (2012). Progressive Gait Deterioration in Adolescents With Dravet Syndrome. *Arch. Neurol.* 69, 873–878. doi:10.1001/archneurol.2011.3275.

Saitoh, M., Ishii, A., Ihara, Y., Hoshino, A., Terashima, H., Kubota, M., et al. (2015a). Missense mutations in sodium channel SCN1A and SCN2A predispose children to encephalopathy with severe febrile seizures. *Epilepsy Res.* 117, 1–6. doi:10.1016/j.eplepsyres.2015.08.001.

Saitoh, M., Shinohara, M., Hoshino, H., Kubota, M., Amemiya, K., Takanashi, J., et al. (2012). Mutations of the SCN1A gene in acute encephalopathy. *Epilepsia* 53, 558–564. doi:10.1111/j.1528-1167.2011.03402.x.

Saitoh, M., Shinohara, M., Ishii, A., Ihara, Y., Hirose, S., Shiomi, M., et al. (2015b). Clinical and genetic features of acute encephalopathy in children taking theophylline. *Brain Dev.* 37, 463–470. doi:10.1016/j.braindev.2014.07.010.

Sanchez-Carpintero, R., Patiño-Garcia, A., and Urrestarazu, E. (2013). Musicogenic seizures in Dravet syndrome. *Dev. Med. Child Neurol.* 55, 668–670. doi:10.1111/dmcn.12138.

Selmer, K., Eriksson, A.-S., Brandal, K., Egeland, T., Tallaksen, C., and Undlien, D. (2009). Parental

SCN1A mutation mosaicism in familial Dravet syndrome. *Clin. Genet.* 76, 398–403. doi:10.1111/j.1399-0004.2009.01208.x.

Selmer, K. K., Egeland, T., Solaas, M. H., Nakken, K. O., Kjeldsen, M. J., Friis, M. L., et al. (2008). Genetic screening of Scandinavian families with febrile seizures and epilepsy or GEFS+. *Acta Neurol. Scand.* 117, 289–292. doi:10.1111/j.1600-0404.2007.00941.x.

Sharkia, R., Hengel, H., Schöls, L., Athamna, M., Bauer, P., and Mahajnah, M. (2016). Parental mosaicism in another case of Dravet syndrome caused by a novel SCN1A deletion: A case report. *J. Med. Case Rep.* 10, 67. doi:10.1186/s13256-016-0854-2.

Shi, X., Yasumoto, S., Nakagawa, E., Fukasawa, T., Uchiya, S., and Hirose, S. (2009). Missense mutation of the sodium channel gene SCN2A causes Dravet syndrome. *Brain Dev.* 31, 758–762. doi:10.1016/j.braindev.2009.08.009.

Shi, Y. W., Yu, M. J., Long, Y. S., Qin, B., He, N., Meng, H., et al. (2012). Mosaic SCN1A mutations in familial partial epilepsy with antecedent febrile seizures. *Genes, Brain Behav.* 11, 170–176. doi:10.1111/j.1601-183X.2011.00756.x.

Singh, N. A., Pappas, C., Dahle, E. J., Claes, L. R. F., Pruess, T. H., De Jonghe, P., et al. (2009). A Role of SCN9A in Human Epilepsies, As a Cause of Febrile Seizures and As a Potential Modifier of Dravet Syndrome. *PLoS Genet.* 5, e1000649. doi:10.1371/journal.pgen.1000649.

Skjei, K. L., Church, E. W., Harding, B. N., Santi, M., Holland-Bouley, K. D., Clancy, R. R., et al. (2015). Clinical and histopathological outcomes in patients with SCN1A mutations undergoing surgery for epilepsy. *J. Neurosurg. Pediatr.* 16, 668–674. doi:10.3171/2015.5.PEDS14551.

Sone, D., Sugawara, T., Sakakibara, E., Tomioka, Y., Taniguchi, G., Murata, Y., et al. (2012). A case of autosomal dominant nocturnal frontal lobe epilepsy (ADNFLE) coexisting with pervasive developmental disorder harboring SCN1A mutation in addition to CHRNB2 mutation. *Epilepsy Behav.* 25, 192–195. doi:10.1016/j.yebeh.2012.07.027.

Spampanato, J. (2004). A Novel Epilepsy Mutation in the Sodium Channel SCN1A Identifies a Cytoplasmic Domain for Subunit Interaction. *J. Neurosci.* 24, 10022–10034. doi:10.1523/JNEUROSCI.2034-04.2004.

Spampanato, J., Escayg, A., Meisler, M. H., and Goldin, A. L. (2001a). Functional effects of two voltage-gated sodium channel mutations that cause generalized epilepsy with febrile seizures plus type 2. *J. Neurosci.* 21, 7481–7490. doi:10.1523/jneurosci.21-19-07481.2001.

Spampanato, J., Escayg, A., Meisler, M. H., and Goldin, A. L. (2003). Generalized epilepsy with febrile seizures plus type 2 mutation W1204R alters voltage-dependent gating of Nav1.1 sodium channels. *Neuroscience* 116, 37–48. doi:10.1016/S0306-4522(02)00698-X.

Specchio, N., Trivisano, M., Balestri, M., Gennaro, E., Specchio, L. M., Fusco, L., et al. (2010). A novel de novo SCN1A missense mutation in Severe Myoclonic Epilepsy Borderland. *Acta Neurol. Belg.* 110, 281–3. Available at: http://www.ncbi.nlm.nih.gov/pubmed/21114141.

Staněk, D., Laššuthová, P., Štěrbová, K., Vlčková, M., Neupauerová, J., Krůtová, M., et al. (2018). Detection rate of causal variants in severe childhood epilepsy is highest in patients with seizure

onset within the first four weeks of life. *Orphanet J. Rare Dis.* 13, 71. doi:10.1186/s13023-018-0812-8.

Stefanaki, E., Aggelakou, V., Orfanou, M., Kokori, E., and Boutoufianakis, S. (2006). Epilepsy with a de novo missense mutation in the sodium channel a1 subunit: A case report. *Acta Paediatr.* 95, 1703–1706. doi:10.1080/08035250600778628.

Stosser, M. B., Lindy, A. S., Butler, E., Retterer, K., Piccirillo-Stosser, C. M., Richard, G., et al. (2018). High frequency of mosaic pathogenic variants in genes causing epilepsy-related neurodevelopmental disorders. *Genet. Med.* 20, 403–410. doi:10.1038/gim.2017.114.

Sugawara, T., Mazaki-Miyazaki, E., Fukushima, K., Shimomura, J., Fujiwara, T., Hamano, S., et al. (2002). Frequent mutations of SCN1A in severe myoclonic epilepsy in infancy. *Neurology* 58, 1122–1124. doi:10.1212/WNL.58.7.1122.

Sugawara, T., Mazaki-Miyazaki, E., Ito, M., Nagafuji, H., Fukuma, G., Mitsudome, A., et al. (2001). Nav1.1 mutations cause febrile seizures associated with afebrile partial seizures. *Neurology* 57, 703–705. doi:10.1212/WNL.57.4.703.

Sugawara, T., Tsurubuchi, Y., Fujiwara, T., Mazaki-Miyazaki, E., Nagata, K., Montal, M., et al. (2003). Nav1.1 channels with mutations of severe myoclonic epilepsy in infancy display attenuated currents. *Epilepsy Res.* 54, 201–207. doi:10.1016/S0920-1211(03)00084-6.

Sugiura, Y., Ogiwara, I., Hoshi, A., Yamakawa, K., and Ugawa, Y. (2012). Different degrees of loss of function between GEFS+ and SMEI Na v1.1 missense mutants at the same residue induced by rescuable folding defects. *Epilepsia* 53, 111–114. doi:10.1111/j.1528-1167.2012.03467.x.

Suls, A., Claeys, K. G., Goossens, D., Harding, B., Luijk, R. Van, Scheers, S., et al. (2006). Microdeletions involving theSCN1A gene may be common inSCN1A-mutation-negative SMEI patients. *Hum. Mutat.* 27, 914–920. doi:10.1002/humu.20350.

Sun, H., Zhang, Y., Liang, J., Liu, X., Ma, X., Qin, J., et al. (2008a). Seven novel SCN1A mutations in Chinese patients with severe myoclonic epilepsy of infancy. *Epilepsia* 49, 1104–1107. doi:10.1111/j.1528-1167.2008.01549_2.x.

Sun, H., Zhang, Y., Liang, J., Liu, X., Ma, X., Wu, H., et al. (2008b). SCN1A, SCN1B, and GABRG2 gene mutation analysis in Chinese families with generalized epilepsy with febrile seizures plus. *J. Hum. Genet.* 53, 769–774. doi:10.1007/s10038-008-0306-y.

Sun, H., Zhang, Y., Liu, X., Ma, X., Yang, Z., Qin, J., et al. (2010). Analysis of SCN1A mutation and parental origin in patients with Dravet syndrome. *J. Hum. Genet.* 55, 421–427. doi:10.1038/jhg.2010.39.

Sun, L., Gilligan, J., Staber, C., Schutte, R. J., Nguyen, V., O'Dowd, D. K., et al. (2012). A Knock-In Model of Human Epilepsy in Drosophila Reveals a Novel Cellular Mechanism Associated with Heat-Induced Seizure. *J. Neurosci.* 32, 14145–14155. doi:10.1523/JNEUROSCI.2932-12.2012.

Surovy, M., Soltysova, A., Kolnikova, M., Sykora, P., Ilencikova, D., Ficek, A., et al. (2016). Novel SCN1A variants in Dravet syndrome and evaluating a wide approach of patient selection. *Gen.*

Physiol. Biophys. 35, 333–342. doi:10.4149/gpb_2016002.

Takayanagi, M., Haginoya, K., Umehara, N., Kitamura, T., Numata, Y., Wakusawa, K., et al. (2010). Acute encephalopathy with a truncation mutation in the SCN1A gene: A case report. *Epilepsia* 51, 1886–1888. doi:10.1111/j.1528-1167.2010.02600.x.

Tan, E. H., Razak, S. A., Abdullah, J. M., and Mohamed Yusoff, A. A. (2012). De-novo mutations and genetic variation in the SCN1A gene in Malaysian patients with generalized epilepsy with febrile seizures plus (GEFS+). *Epilepsy Res.* 102, 210–215. doi:10.1016/j.eplepsyres.2012.08.004.

Tang, S., Lin, J. P., Hughes, E., Siddiqui, A., Lim, M., and Lascelles, K. (2011). Encephalopathy and SCN1A mutations. *Epilepsia* 52, e26–e30. doi:10.1111/j.1528-1167.2011.03019.x.

Tiefes, A. M., Hartlieb, T., Tacke, M., von Stülpnagel-Steinbeis, C., Larsen, L. H. G., Hao, Q., et al. (2019). Mesial Temporal Sclerosis in SCN1A -Related Epilepsy: Two Long-Term EEG Case Studies. *Clin. EEG Neurosci.* 50, 267–272. doi:10.1177/1550059418794347.

Tonekaboni, S. H., Ebrahimi, A., Bakhshandeh Bali, M. K., Taheri Otaghsara, S. M., Houshmand, M., Nasehi, M. M., et al. (2013). Sodium channel gene mutations in Children with GEFS+ and Dravet syndrome: A cross sectional study. *Iran. J. Child Neurol.* 7, 31–36. doi:10.22037/ijcn.v7i2.4074.

Trujillano, D., Bertoli-Avella, A. M., Kumar Kandaswamy, K., Weiss, M. E., Köster, J., Marais, A., et al. (2017). Clinical exome sequencing: results from 2819 samples reflecting 1000 families. *Eur. J. Hum. Genet.* 25, 176–182. doi:10.1038/ejhg.2016.146.

Trump, N., McTague, A., Brittain, H., Papandreou, A., Meyer, E., Ngoh, A., et al. (2016). Improving diagnosis and broadening the phenotypes in early-onset seizure and severe developmental delay disorders through gene panel analysis. *J. Med. Genet.* 53, 310–317. doi:10.1136/jmedgenet-2015-103263.

Tsai, M.-H., Chan, C.-K., Chang, Y.-C., Lin, C.-H., Liou, C.-W., Chang, W.-N., et al. (2018). Molecular Genetic Characterization of Patients With Focal Epilepsy Using a Customized Targeted Resequencing Gene Panel. *Front. Neurol.* 9. doi:10.3389/fneur.2018.00515.

Tsuji, M., Mazaki, E., Ogiwara, I., Wada, T., Iai, M., Okumura, A., et al. (2011). Acute Encephalopathy in a Patient with Dravet Syndrome. *Neuropediatrics* 42, 78–81. doi:10.1055/s-0031-1279725.

Tuncer, F. N., Gormez, Z., Calik, M., Altiokka Uzun, G., Sagiroglu, M. S., Yuceturk, B., et al. (2015). A clinical variant in SCN1A inherited from a mosaic father cosegregates with a novel variant to cause Dravet syndrome in a consanguineous family. *Epilepsy Res.* 113, 5–10. doi:10.1016/j.eplepsyres.2015.02.020.

Usluer, S., Salar, S., Arslan, M., Yiş, U., Kara, B., Tektürk, P., et al. (2016). SCN1A gene sequencing in 46 Turkish epilepsy patients disclosed 12 novel mutations. *Seizure* 39, 34–43. doi:10.1016/j.seizure.2016.05.008.

Vadlamudi, L., Dibbens, L. M., Lawrence, K. M., Iona, X., McMahon, J. M., Murrell, W., et al.

(2010). Timing of De Novo Mutagenesis — A Twin Study of Sodium-Channel Mutations. *N. Engl. J. Med.* 363, 1335–1340. doi:10.1056/NEJMoa0910752.

Vanmolkot, K. R. J., Babini, E., de Vries, B., Stam, A. H., Freilinger, T., Terwindt, G. M., et al. (2007). The novel p.L1649Q mutation in the SCN1A epilepsy gene is associated with familial hemiplegic migraine: genetic and functional studies. *Hum. Mutat.* 28, 522–522. doi:10.1002/humu.9486.

Veeramah, K. R., Johnstone, L., Karafet, T. M., Wolf, D., Sprissler, R., Salogiannis, J., et al. (2013). Exome sequencing reveals new causal mutations in children with epileptic encephalopathies. *Epilepsia* 54, 1270–1281. doi:10.1111/epi.12201.

Verbeek, N. E., van der Maas, N. A. T., Jansen, F. E., van Kempen, M. J. A., Lindhout, D., and Brilstra, E. H. (2013). Prevalence of SCN1A-Related Dravet Syndrome among Children Reported with Seizures following Vaccination: A Population-Based Ten-Year Cohort Study. *PLoS One* 8, e65758. doi:10.1371/journal.pone.0065758.

Verbeek, N. E., van Kempen, M., Gunning, W. B., Renier, W. O., Westland, B., Lindhout, D., et al. (2011). Adults with a history of possible Dravet syndrome: An illustration of the importance of analysis of the SCN1A gene. *Epilepsia* 52, e23–e25. doi:10.1111/j.1528-1167.2011.02982.x.

Villeneuve, N., Laguitton, V., Viellard, M., Lépine, A., Chabrol, B., Dravet, C., et al. (2014). Cognitive and adaptive evaluation of 21 consecutive patients with Dravet syndrome. *Epilepsy Behav.* 31, 143–148. doi:10.1016/j.yebeh.2013.11.021.

Volkers, L., Kahlig, K. M., Verbeek, N. E., Das, J. H. G., van Kempen, M. J. A., Stroink, H., et al. (2011). Na v1.1 dysfunction in genetic epilepsy with febrile seizures-plus or Dravet syndrome. *Eur. J. Neurosci.* 34, 1268–1275. doi:10.1111/j.1460-9568.2011.07826.x.

Wallace, R. H., Hodgson, B. L., Grinton, B. E., Gardiner, R. M., Robinson, R., Rodriguez-Casero, V., et al. (2003). Sodium channel 1-subunit mutations in severe myoclonic epilepsy of infancy and infantile spasms. *Neurology* 61, 765–769. doi:10.1212/01.WNL.0000086379.71183.78.

Wallace, R. H., Scheffer, I. E., Barnett, S., Richards, M., Dibbens, L., Desai, R. R., et al. (2001). Neuronal sodium-channel β1-subunit mutations in generalized epilepsy with febrile seizures plus. *Am. J. Hum. Genet.* 68, 859–865. doi:10.1086/319516.

Wang, J., Kurahashi, H., Ishii, A., Kojima, T., Ohfu, M., Inoue, T., et al. (2008). Microchromosomal deletions involving SCN1A and adjacent genes in severe myoclonic epilepsy in infancy. *Epilepsia* 49, 1528–1534. doi:10.1111/j.1528-1167.2008.01609.x.

Wang, J. W., Shi, X. Y., Kurahashi, H., Hwang, S. K., Ishii, A., Higurashi, N., et al. (2012). Prevalence of SCN1A mutations in children with suspected Dravet syndrome and intractable childhood epilepsy. *Epilepsy Res.* 102, 195–200. doi:10.1016/j.eplepsyres.2012.06.006.

Wang, Y., Du, X., Bin, R., Yu, S., Xia, Z., Zheng, G., et al. (2017). Genetic Variants Identified from Epilepsy of Unknown Etiology in Chinese Children by Targeted Exome Sequencing. *Sci. Rep.* 7, 40319. doi:10.1038/srep40319.

Wei, C.-M., Xia, G.-Z., and Ren, R.-N. (2018). [Gene mutations in unexplained infantile epileptic encephalopathy: an analysis of 47 cases]. *Zhongguo Dang Dai Er Ke Za Zhi* 20, 125–129. doi:10.7499/j.issn.1008-8830.2018.02.009.

Weiss, L. A., Escayg, A., Kearney, J. A., Trudeau, M., MacDonald, B. T., Mori, M., et al. (2003). Sodium channels SCN1A, SCN2A and SCN3A in familial autism. *Mol. Psychiatry* 8, 186–194. doi:10.1038/sj.mp.4001241.

Weller, C. M., Pelzer, N., de Vries, B., López, M. A., De Fàbregues, O., Pascual, J., et al. (2014). Two novel SCN1A mutations identified in families with familial hemiplegic migraine. *Cephalalgia* 34, 1062–1069. doi:10.1177/0333102414529195.

Willemsen, M. H., Rensen, J. H. M., van Schrojenstein-Lantman de Valk, H. M. J., Hamel, B. C. J., and Kleefstra, T. (2012). Adult Phenotypes in Angelman- and Rett-Like Syndromes. *Mol. Syndromol.* 2, 217–234. doi:10.1159/000335661.

Wu, Y. W., Sullivan, J., McDaniel, S. S., Meisler, M. H., Walsh, E. M., Li, S. X., et al. (2015). Incidence of dravet syndrome in a US population. *Pediatrics* 136, e1310–e1315. doi:10.1542/peds.2015-1807.

Xiong, H. Y., Alipanahi, B., Lee, L. J., Bretschneider, H., Merico, D., Yuen, R. K. C., et al. (2015). The human splicing code reveals new insights into the genetic determinants of disease. *Science (80-.).* 347, 1254806–1254806. doi:10.1126/science.1254806.

Xu, X., Yang, X., Wu, Q., Liu, A., Yang, X., Ye, A. Y., et al. (2015). Amplicon Resequencing Identified Parental Mosaicism for Approximately 10% of " de novo " SCN1A Mutations in Children with Dravet Syndrome. *Hum. Mutat.* 36, 861–872. doi:10.1002/humu.22819.

Xu, X., Zhang, Y., Sun, H., Liu, X., Yang, X., Xiong, H., et al. (2014). Early clinical features and diagnosis of Dravet syndrome in 138 Chinese patients with SCN1A mutations. *Brain Dev.* 36, 676–681. doi:10.1016/j.braindev.2013.10.004.

Yang, X., Liu, A., Xu, X., Yang, X., Zeng, Q., Ye, A. Y., et al. (2017). Genomic mosaicism in paternal sperm and multiple parental tissues in a Dravet syndrome cohort. *Sci. Rep.* 7, 15677. doi:10.1038/s41598-017-15814-7.

Yordanova, I., Todorov, T., Dimova, P., Hristova, D., Tincheva, R., Litvinenko, I., et al. (2011). One novel Dravet syndrome causing mutation and one recurrent MAE causing mutation in SCN1A gene. *Neurosci. Lett.* 494, 180–183. doi:10.1016/j.neulet.2011.03.008.

Yu, M.-J., Shi, Y.-W., Gao, M.-M., Deng, W.-Y., Liu, X.-R., Chen, L., et al. (2010). Milder phenotype with SCN1A truncation mutation other than SMEI. *Seizure* 19, 443–445. doi:10.1016/j.seizure.2010.06.010.

Zhang, Y., Chen, N., Zhou, M., Guo, J., Guo, J., and He, L. (2017). A novel SCN1A mutation identified in a Chinese family with familial hemiplegic migraine: A case report. *Cephalalgia* 37, 1294–1298. doi:10.1177/0333102416677049.

Zhang, Y., Kong, W., Gao, Y., Liu, X., Gao, K., Xie, H., et al. (2015). Gene Mutation Analysis in 253 Chinese Children with Unexplained Epilepsy and Intellectual/Developmental Disabilities.

PLoS One 10, e0141782. doi:10.1371/journal.pone.0141782.

Zhou, P., He, N., Zhang, J. W., Lin, Z. J., Wang, J., Yan, L. M., et al. (2018). Novel mutations and phenotypes of epilepsy-associated genes in epileptic encephalopathies. *Genes, Brain Behav.* 17, e12456. doi:10.1111/gbb.12456.

Zhu, X., Padmanabhan, R., Copeland, B., Bridgers, J., Ren, Z., Kamalakaran, S., et al. (2017). A case-control collapsing analysis identifies epilepsy genes implicated in trio sequencing studies focused on de novo mutations. *PLoS Genet.* 13, e1007104. doi:10.1371/journal.pgen.1007104.

Zuberi, S. M., Brunklaus, A., Birch, R., Reavey, E., Duncan, J., and Forbes, G. H. (2011). Genotype-phenotype associations in SCN1A-related epilepsies. *Neurology* 76, 594–600. doi:10.1212/WNL.0b013e31820c309b.

Zucca, C., Redaelli, F., Epifanio, R., Zanotta, N., Romeo, A., Lodi, M., et al. (2008). Cryptogenic Epileptic Syndromes Related to SCN1A. *Arch. Neurol.* 65, 489. doi:10.1001/archneur.65.4.489.
